# Effects of oils and solid fats on blood lipids: a systematic review and network meta-analysis[S]

**DOI:** 10.1194/jlr.P085522

**Published:** 2018-07-13

**Authors:** Lukas Schwingshackl, Berit Bogensberger, Aleksander Benčič, Sven Knüppel, Heiner Boeing, Georg Hoffmann

**Affiliations:** Department of Epidemiology,* German Institute of Human Nutrition Potsdam-Rehbruecke (DIfE), 14558 Nuthetal, Germany; NutriAct-Competence Cluster Nutrition Research Berlin-Potsdam,† Germany; Department of Nutritional Sciences,§ University of Vienna, 1090 Vienna, Austria

**Keywords:** fatty acids, cardiovascular disease, evidence synthesis

## Abstract

The aim of this network meta-analysis (NMA) is to compare the effects of different oils/solid fats on blood lipids. Literature searches were performed until March 2018. Inclusion criteria were as follows: *i*) randomized trial (≥3 weeks study length) comparing at least two of the following oils/solid fats: safflower, sunflower, rapeseed, hempseed, flaxseed, corn, olive, soybean, palm, and coconut oil, and lard, beef-fat, and butter; *ii*) outcomes LDL-cholesterol (LDL-C), total cholesterol (TC), HDL-cholesterol (HDL-C), and triacylglycerols (TGs). A random dose-response (per 10% isocaloric exchange) NMA was performed and surface under the cumulative ranking curve (SUCRA) was estimated. Fifty-four trials were included in the NMA. Safflower oil had the highest SUCRA value for LDL-C (82%) and TC (90%), followed by rapeseed oil (76% for LDL-C, 85% for TC); whereas, palm oil (74%) had the highest SUCRA value for TG, and coconut oil (88%) for HDL-C. Safflower, sunflower, rapeseed, flaxseed, corn, olive, soybean, palm, and coconut oil as well beef fat were more effective in reducing LDL-C (−0.42 to −0.23 mmol/l) as compared with butter. Despite limitations in these data, our NMA findings are in line with existing evidence on the metabolic effects of fat and support current recommendations to replace high saturated-fat food with unsaturated oils.

According to the most recent report from the Global Burden of Disease Study, CVD is the leading cause of death worldwide (1). Dyslipidemia is one of the most important modifiable risk factors for the development of CVD (2). The controversial association between dietary fatty acids, blood lipids, and CVD has been intensively studied for more than a half century (3). It is well-established that saturated fatty acids (SFAs), when replaced with either PUFAs or MUFAs, decrease LDL-cholesterol (LDL-C), a strong risk factor for CVD (4). Moreover, consuming PUFAs instead of SFAs reduced coronary heart disease (CHD) events in randomized controlled trials (RCTs) (5). Paradoxically, some studies reported that replacement of SFAs in the diet with linoleic acid reduced serum cholesterol levels, but did not lower risk of CHD mortality (6).

A major disadvantage when analyzing dietary fatty acids is the limited interpretation when compared with the more realistic analyses of specific oils and solid fats. Moreover, findings on dietary acids are more difficult to transfer into recommendations on primary prevention of noncommunicable chronic diseases (7). Pairwise meta-analyses showed that n-3- and n-6-rich plant oils were more effective in reducing LDL-C and total cholesterol (TC) compared with olive oil (8), whereas palm oil consumption increased LDL-C considerably more than vegetable oils low in SFAs (9). Although, the effects of oils on blood lipids can be predicted from their fatty acid composition (10), one question that still remains to be answered is: which type of oils/solid fats offers the greatest improvements on blood lipids, combining direct and indirect evidence? To address this issue in the present systematic review, we used the methodology of network meta-analysis (NMA), which enables a simultaneous comparison of intervention trials (11).

NMA combines direct (i.e., from trials comparing two interventions directly) and indirect (i.e., from a connected root via one or more intermediate comparators) evidence in a network of trials (**Fig. 1**). In this way, it enables inference about every possible comparison between a pair of interventions in the network, even when some comparisons have never been evaluated in a trial. In a theoretical example, none of the studies have compared B (butter) and C (palm oil), but each has been compared with a common comparator A (olive oil), then we assume an indirect comparison of B and C on the direct comparison of B and A and the direct comparison of C and A (Fig. 1).

**Fig. 1.**
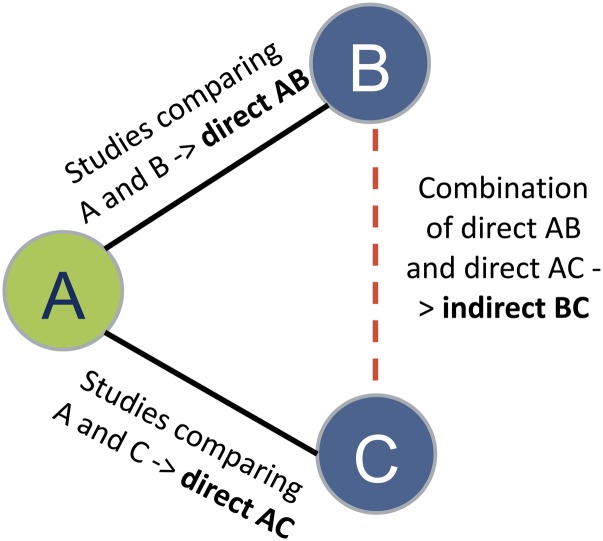
Example of indirect relative effects in a hypothetical triangle comparing three interventions (A: olive oil; B: butter; C: palm oil).

To the best of our knowledge, no study has been conducted to date that simultaneously compares different oils/solid fats on blood lipids.

In brief, the aim of the present study was to compare the effects of 13 different oils and solid fats across randomized trials on established blood lipid factors [TC, LDL-C, HDL-cholesterol (HDL-C), and triacylglycerols (TGs)] using NMA methodology.

## METHODS AND DESIGN

This review was registered in PROSPERO International Prospective Register of Systematic Reviews (https://www.crd.york.ac.uk/prospero/display_record.php?RecordID=56513). The present NMA was planned, conducted, and reported in adherence to standards of quality for reporting NMAs (12, 13).

### Search strategy

The literature search was performed using the electronic databases PubMed and Cochrane Central Register of Controlled Trials (CENTRAL) until March 2018 with no restriction of language and calendar date using a predefined search strategy (supplemental Appendix S1).

The reference lists from the identified articles were screened to search for additional relevant studies. Searches were conducted by two authors (B.B., L.S.) with disagreements being resolved by involvement of other authors (G.H., H.B.).

### Eligibility criteria

Studies were included in the NMA if they met all of the following criteria: *i*) randomized study (parallel or cross-over design) examining diets varying in composition of at least two of the following oils/solid fats: safflower, sunflower, rapeseed, hempseed, flaxseed, corn, olive, soybean, palm, and coconut oil, and lard, beef fat, and butter; *ii*) comparison of isocaloric exchange of the different oils/solid fats within a trial; *iii*) minimum intervention period of 3 weeks; *iv*) patients with a mean age ≥18 years; *v*) outcomes including: LDL-C (defined as primary outcome in the present NMA) and TC, HDL-C, and TGs (defined as secondary outcomes in the present NMA).

The following studies were excluded: *i*) RCTs including pregnant women, children and adolescents, and patients with cancer, hemodialysis, or type 1 diabetes; *ii*) RCTs of acute (single meal) postprandial effects only; *iii*) RCTs using mixed oils or using butter plus mixed oils in the intervention arms; *iv*) RCTs using encapsulated oil supplements; *v*) RCTs using fish oils or MCT oils, or omega-3 fatty enriched oils/solid fats; *vi*) RCTs implementing enrichment of oils/solid fats with plant sterols, plant stanols; *vii*) RCTs based on liquid/formula diets; *viii*) co-intervention (e.g., drug, diet, or exercise) not applied in all intervention arms.

### Data extraction

After determination of the study selection, one reviewer extracted the following characteristics: name of first author, year of publication, study origin (country), study design (RCT: parallel or cross-over), comparison of oils/solid fats, sample size (completers), disease status (i.e., healthy, obese, hypercholesterolemia, peripheral disease), mean age, mean BMI, percent type 2 diabetics, percent female, study length (weeks), specification of the intervention arms (type of oil/solid fat used and amount of intake; provided by investigators or simply advice), underlying type of diet (i.e., habitual, healthy; provided by investigators or simply advice; weight loss: yes vs. no), primary outcome of the study, outcomes extracted for the present NMA, and conflict of interest. Outcome data included postintervention values with corresponding standard deviations. The extracted information was verified by a second reviewer (A.B. or L.S.).

### Risk of bias assessment

Full copies of the studies were assessed by two authors (L.S., B.B.) for methodological quality using the risk of bias assessment tool from the Cochrane Collaboration (14). The following sources of bias were assessed: selection bias (random sequence generation and allocation concealment), performance bias (blinding of participants and personnel), detection bias (blinding of outcome assessment), attrition bias (incomplete outcome data), and reporting bias (selective reporting).

Studies were classified as being at either low risk of bias (if at least three out of a maximum of six items were rated as low risk and no item was rated with a high risk of bias), high risk of bias (if at least one item was rated with a high risk of bias), or moderate/unclear risk of bias (all other studies).

### Data synthesis

#### Description of the available data.

For all included trials, we present study and population characteristics describing the available data and important variables (e.g., age, BMI, length of follow-up, sample size, percent female, disease status, and specification of diet). We illustrated the available direct comparisons between different oils/solid fats using a network diagram for each outcome (15). The size of the nodes is proportional to the sample size of each dietary intervention and the thickness of the lines proportional to the number of studies available.

#### Assessment of transitivity.

To evaluate the assumption of transitivity, we compared the distribution of the potential effect modifiers across the available direct comparisons. We considered the following effect modifiers: age, BMI, study length, and sample size.

#### Statistical analysis.

For each outcome measure of interest, we performed random effects NMA in order to determine the pooled relative effect of each intervention against every other measure in terms of the postintervention values. We assessed the similarity of trials within each direct comparison. NMA was then used to synthesize the direct and indirect effects. The method of NMA is an extension of the standard pairwise meta-analysis that enables a simultaneous comparison of multiple interventions, forming a connected network while preserving the internal randomization of individual trials. We ran random effects dose-response NMA (intake of oils/solid fats was standardized and presented per 10% isocaloric exchange) for each outcome to estimate all possible pairwise relative effects and to obtain a clinically meaningful relative ranking of the different dietary interventions. We present the summary mean differences with their 95% CI in a league table. The relative ranking of the different fats and oils for each outcome were estimated using the distribution of the ranking probabilities and the surface under the cumulative ranking curve (SUCRA) (16). The SUCRA ranges between 0% (i.e., the treatment always ranks last) and 100% (i.e., the treatment always ranks first).

We fitted all analyses described in a frequentist framework using Stata (17) [network package (18)] and produced presentation tools with the network graphs package (19).

#### Assessment of inconsistency.

To evaluate the presence of statistical inconsistency in the data (i.e., disagreement between the different sources of evidence), we employed both local and global approaches (20). Specifically, we used the loop-specific approach (21) to detect loops of evidence that might present important inconsistency as well as the side-splitting approach (22) to detect comparisons for which direct estimates disagree with indirect evidence from the entire network. Global methods investigate the presence of inconsistency jointly from all possible sources in the entire network simultaneously. For this purpose, we used the design-by-treatment interaction model (23, 24).

#### Sensitivity analyses.

Sensitivity analyses were conducted by including low risk of bias trials: trials where oils/solid fats were provided by investigators only and trials including healthy participants.

#### Small study effects and publication bias.

We produced the comparison-adjusted funnel plot (15) to assess the magnitude of funnel plot asymmetry for all outcomes.

### Credibility of the evidence

To make inferences about the credibility of evidence from the NMA, we used the GRADE system extended for NMA following the approach suggested by Salanti et al. (20) for all outcomes.

## RESULTS

Out of 4,901 records identified by the literature search, 241 full text articles were assessed in detail, as they reported on one or more of the types of oils/solid fats of interest in the title/abstract (supplemental Fig. S1).

Overall, 54 trials (55 reports) (25–79) with 2,065 participants published between 1984 and 2018 were included in the NMA.

The RCTs’ length ranged between 3 and 27 weeks; the mean age of the participants was between 22 and 84 years and their BMI between 20.2 and 31.1 kg/m^2^. Fourteen trials were conducted in North America, 2 trials in South America, 24 trials in Europe, 12 trials in Asia, and 1 trial in Australia and in Africa. Twenty-three trials were conducted in healthy participants; and in 50 trials, oils/solid fats were provided by investigators. Study and participant characteristics as well as postintervention means and standard deviations of the included trials according to study arms are summarized in supplemental Tables S1 and S2, respectively. The fatty acid composition of the different oils/solid fats are given in supplemental Table S3.

Thirty-three trials (61%) were judged to be low risk of bias, 3 trials to be high risk of bias, and 18 trials were classified as moderate/unclear risk of bias studies, mainly due to insufficient information available within the included trials regarding random sequence generation, allocation concealment, and blinding of participants/personnel and outcome assessment. With regard to the single risk of bias items, 28% of the included studies indicated a low risk of bias for random-sequence generation, 7% for allocation concealment, and 39% for blinding of participants and personnel (supplemental Fig. S2).

**Fig. 2.**
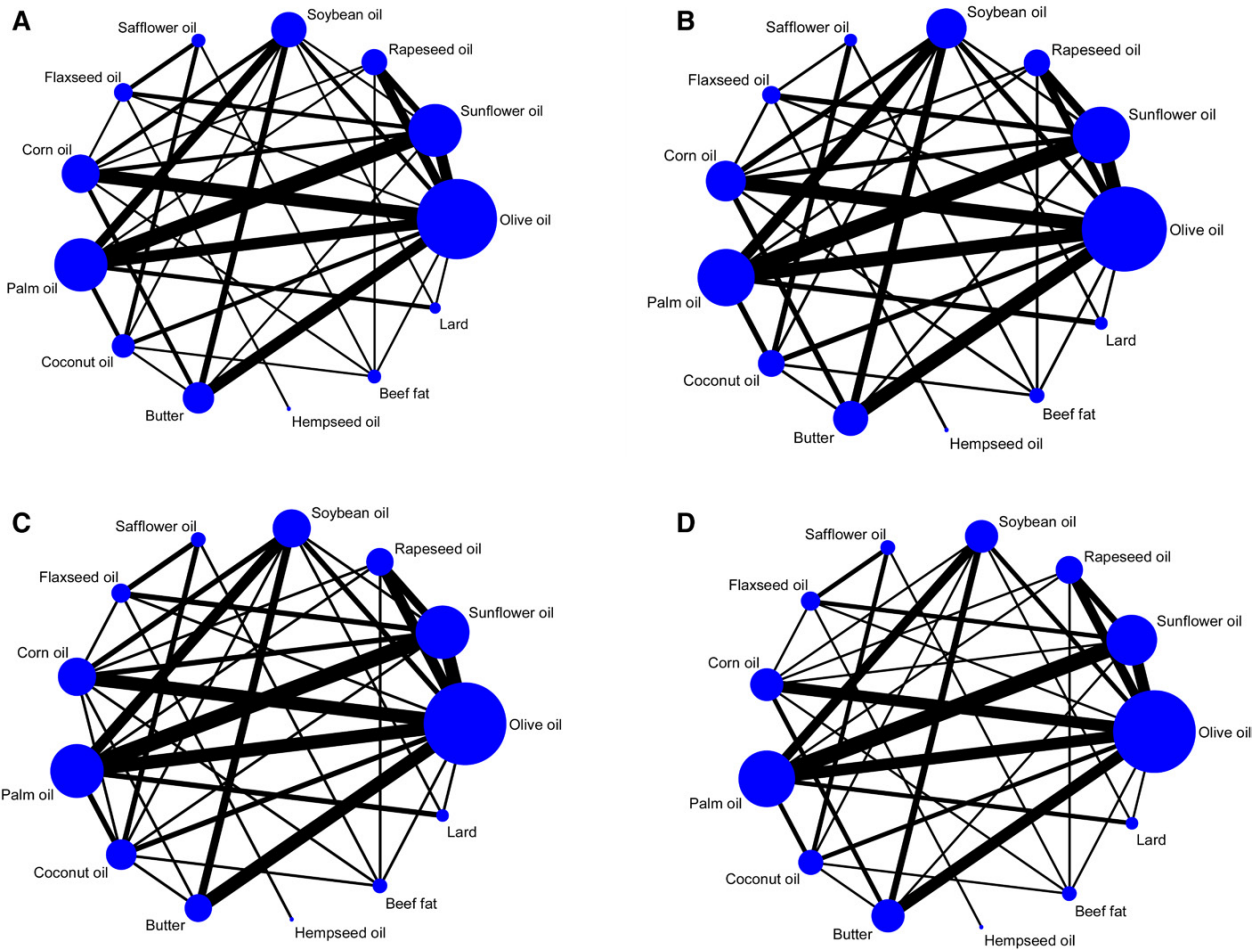
Network diagram for TC (A), LDL-C (B), HDL-C (C), and TGs (D). The size of the nodes is proportional to the total number of participants allocated to the intervention and the thickness of the lines is proportional to the number of studies evaluating each direct comparison.

**Figure 2** shows the network diagrams for TC (Fig. 2A), LDL-C (Fig. 2B), HDL-C (Fig. 2C), and TG (Fig. 2D) of direct comparison with the number of studies reflected by the size of the edges, and the number of participants reflected by the size of the nodes. The highest number of trials compared a sunflower oil arm to a palm oil arm (n = 7).

Supplemental Tables S4–S7 show the percentage of statistical contribution coming from direct and indirect comparisons for each fat or oil compared with each other for TC, LDL-C, HDL-C, and TG. It was shown that the contribution to the study effects came more often from indirect comparisons.

In general, there were some differences in the examined effect modifiers across comparisons for BMI, mean age, study length, and sample size. For several comparisons, we did not have enough studies and thus could not test transitivity appropriately (supplemental Figs. S3–S6).

The summary effect estimates for the comparison of different oils/solid fats on LDL-C, TG, TC, and HDL-C are shown in **Table 1** and **Table 2**.

**TABLE 1.** The summary effect estimates for the comparison of different oils/solid fats on LDL-C and TG

LDL-C (mmol/l)												
Safflower oil	−0.07 (−0.31, 0.16)	−0.05 (−0.30, 0.19)	−0.02 (−0.53, 0.49)	−0.05 (−0.35, 0.25)	−0.09 (−0.32, 0.15)	−0.16 (−0.39, 0.06)	−0.13 (−0.37, 0.10)	−0.18 (−0.41, 0.05)	−0.19 (−0.38, 0.01)	−0.14 (−0.35, 0.08)	**−0.33 (−0.62, −0.04)**	**−0.42 (−0.65, −0.18)**
−0.01 (−0.08, 0.06)	Sunflower oil	0.02 (−0.11, 0.15)	0.05 (−0.41, 0.52)	0.02 (−0.19, 0.23)	−0.01 (−0.12, 0.09)	**−0.09 (−0.17, −0.01)**	−0.06 (−0.16, 0.04)	**−0.10 (−0.19, −0.02)**	−0.11 (−0.27, 0.05)	−0.06 (−0.27, 0.15)	**−0.26 (−0.46, −0.06)**	**−0.34 (−0.46, −0.23)**
−0.01 (−0.09, 0.06)	−0.00 (−0.05, 0.05)	Rapeseed oil	0.03 (−0.45, 0.52)	0.00 (−0.24, 0.24)	−0.03 (−0.18, 0.11)	−0.11 (−0.24, 0.02)	−0.08 (−0.22, 0.06)	−0.12 (−0.26, 0.01)	−0.13 (−0.31, 0.05)	−0.08 (−0.31, 0.14)	**−0.28 (−0.50, −0.06)**	**−0.36 (−0.52, −0.21)**
0.01 (−0.17, 0.19)	0.02 (−0.16, 0.19)	0.02 (−0.16, 0.20)	Hempseed oil	−0.03 (−0.45, 0.38)	−0.07 (−0.54, 0.41)	−0.14 (−0.61, 0.33)	−0.11 (−0.59, 0.36)	−0.16 (−0.63, 0.31)	−0.17 (−0.65, 0.32)	−0.12 (−0.62, 0.39)	−0.31 (−0.82, 0.19)	−0.40 (−0.88, 0.08)
−0.01 (−0.11, 0.09)	−0.00 (−0.09, 0.08)	−0.00 (−0.10, 0.09)	−0.02 (−0.17, 0.13)	Flaxseed oil	−0.04 (−0.26, 0.19)	−0.11 (−0.33, 0.10)	−0.08 (−0.31, 0.14)	−0.12 (−0.35, 0.10)	−0.13 (−0.38, 0.12)	−0.08 (−0.37, 0.21)	−0.28 (−0.56, 0.00)	**−0.37 (−0.60, −0.13)**
−0.00 (−0.08, 0.07)	0.01 (−0.05, 0.06)	0.01 (−0.06, 0.07)	−0.01 (−0.19, 0.17)	0.01 (−0.09, 0.11)	Corn oil	−0.07 (−0.17, 0.02)	−0.04 (−0.15, 0.06)	−0.09 (−0.20, 0.02)	−0.10 (−0.26, 0.07)	−0.05 (−0.26, 0.17)	**−0.25 (−0.45, −0.04)**	**−0.33 (−0.45, −0.21)**
−0.04 (−0.10, 0.03)	−0.03 (−0.06, 0.00)	−0.02 (−0.07, 0.02)	−0.04 (−0.22, 0.13)	−0.02 (−0.11, 0.06)	−0.03 (−0.08, 0.02)	Olive oil	0.03 (−0.06, 0.12)	−0.01 (−0.10, 0.07)	−0.02 (−0.17, 0.13)	0.03 (−0.18, 0.23)	−0.17 (−0.36, 0.02)	**−0.25 (−0.36, −0.15)**
0.00 (−0.07, 0.07)	0.01 (−0.03, 0.05)	0.01 (−0.04, 0.06)	−0.01 (−0.18, 0.17)	0.01 (−0.08, 0.10)	0.00 (−0.05, 0.06)	0.04 (0.01, 0.06)	Soybean oil	−0.04 (−0.13, 0.05)	−0.05 (−0.21, 0.10)	−0.00 (−0.22, 0.21)	**−0.20 (−0.40, −0.01)**	**−0.29 (−0.39, −0.18)**
0.00 (−0.06, 0.07)	0.01 (−0.03, 0.05)	0.02 (−0.04, 0.07)	−0.00 (−0.18, 0.17)	0.02 (−0.07, 0.11)	0.01 (−0.05, 0.07)	0.04 (−0.00, 0.08)	0.00 (−0.04, 0.05)	Palm oil	−0.01 (−0.16, 0.15)	0.04 (−0.17, 0.25)	−0.16 (−0.34, 0.03)	**−0.24 (−0.36, −0.12)**
−0.04 (−0.10, 0.01)	−0.03 (−0.07, 0.01)	−0.03 (−0.08, 0.02)	−0.05 (−0.22, 0.13)	−0.03 (−0.12, 0.06)	−0.04 (−0.10, 0.02)	−0.00 (−0.04, 0.03)	−0.04 (−0.08, −0.00)	**−0.04 (−0.08, −0.01)**	Coconut oil	0.05 (−0.14, 0.24)	−0.15 (−0.38, 0.09)	**−0.23 (−0.40, −0.07)**
**−0.08 (−0.14, −0.03)**	**−0.07 (−0.14, −0.01)**	−0.07 (−0.14, −0.00)	−0.09 (−0.27, 0.09)	−0.07 (−0.17, 0.03)	**−0.08 (−0.15, −0.01)**	−0.05 (−0.10, 0.01)	**−0.08 (−0.15, −0.02)**	**−0.09 (−0.15, −0.03)**	−0.04 (−0.09, 0.01)	Beef fat	−0.20 (−0.47, 0.08)	**−0.28 (−0.50, −0.06)**
−0.02 (−0.13, 0.09)	−0.01 (−0.11, 0.09)	−0.01 (−0.11, 0.10)	−0.02 (−0.22, 0.17)	−0.00 (−0.13, 0.12)	−0.01 (−0.12, 0.09)	0.02 (−0.08, 0.11)	−0.02 (−0.11, 0.08)	−0.02 (−0.12, 0.07)	0.02 (−0.08, 0.12)	0.07 (−0.04, 0.17)	Lard	−0.08 (−0.29, 0.13)
−0.05 (−0.12, 0.01)	**−0.04 (−0.08, −0.01)**	−0.04 (−0.09, 0.01)	−0.06 (−0.24, 0.11)	−0.04 (−0.13, 0.05)	−0.05 (−0.10, 0.00)	−0.02 (−0.04, 0.01)	**−0.06 (−0.08, −0.03)**	**−0.06 (−0.10, −0.01)**	−0.01 (−0.05, 0.03)	0.03 (−0.03, 0.09)	−0.04 (−0.13, 0.06)	Butter
TGs (mmol/l)												

The values above the oils/solid fats correspond to the difference in mean (per 10% isocaloric exchange) (95% CI) in LDL-C (mmol/l) between the row and columns (e.g., the mean difference in average LDL-C between safflower oil and butter is −0.42 mmol/l). The values below the oils/solid fats correspond to the difference in mean in TGs (mmol/l) between the column and the row (e.g., the mean difference in average TGs between safflower oil and butter is −0.05 mmol/l). Bold indicates significant effects (95% confidence interval does not overlap zero).

**TABLE 2.** The summary effect estimates for the comparison of different oils/solid fats on HDL-C and TC

HDL-C (mmol/l)												
Safflower oil	**−0.06 (−0.11, −0.01)**	−0.06 (−0.11, 0.00)	−0.07 (−0.28, 0.13)	−0.05 (−0.12, 0.02)	−0.04 (−0.09, 0.01)	**−0.06 (−0.10, −0.01)**	−0.02 (−0.07, 0.03)	**−0.08 (−0.12, −0.03)**	**−0.09 (−0.13, −0.05)**	**−0.08 (−0.12, −0.03)**	−0.06 (−0.12, 0.00)	−0.05 (−0.10, 0.01)
−0.12 (−0.33, 0.10)	Sunflower oil	0.00 (−0.03, 0.04)	−0.02 (−0.22, 0.19)	0.01 (−0.04, 0.06)	0.02 (−0.01, 0.05)	0.00 (−0.02, 0.02)	**0.04 (0.01, 0.06)**	−0.02 (−0.04, 0.00)	−0.03 (−0.06, 0.00)	−0.02 (−0.06, 0.03)	−0.00 (−0.04, 0.04)	0.01 (−0.02, 0.04)
−0.06 (−0.29, 0.17)	0.06 (−0.08, 0.19)	Rapeseed oil	−0.02 (−0.23, 0.19)	0.01 (−0.06, 0.07)	0.02 (−0.02, 0.06)	0.00 (−0.03, 0.04)	0.03 (−0.00, 0.07)	−0.02 (−0.06, 0.02)	−0.03 (−0.07, 0.01)	−0.02 (−0.07, 0.03)	−0.00 (−0.05, 0.05)	0.01 (−0.03, 0.05)
−0.14 (−0.61, 0.33)	−0.02 (−0.45, 0.41)	−0.08 (−0.52, 0.37)	Hempseed oil	0.02 (−0.17, 0.22)	0.04 (−0.17, 0.24)	0.02 (−0.19, 0.22)	0.05 (−0.15, 0.26)	−0.00 (−0.21, 0.20)	−0.01 (−0.22, 0.19)	−0.00 (−0.21, 0.21)	0.02 (−0.19, 0.22)	0.03 (−0.18, 0.24)
−0.16 (−0.45, 0.13)	−0.04 (−0.27, 0.18)	−0.10 (−0.36, 0.16)	−0.02(−0.39, 0.34)	Flaxseed oil	0.01 (−0.05, 0.07)	−0.01 (−0.06, 0.05)	0.03 (−0.03, 0.08)	−0.03 (−0.08, 0.03)	−0.04 (−0.10, 0.02)	−0.03 (−0.09, 0.04)	−0.01 (−0.07, 0.06)	0.01 (−0.05, 0.06)
−0.12 (−0.34, 0.10)	−0.00 (−0.11, 0.11)	−0.06 (−0.21, 0.09)	0.02 (−0.42, 0.46)	0.04 (−0.20, 0.28)	Corn oil	−0.02 (−0.04, 0.01)	0.02 (−0.01, 0.04)	**−0.04 (−0.06, −0.01)**	**−0.05 (−0.08, −0.01)**	−0.04 (−0.08, 0.01)	−0.02 (−0.06, 0.02)	−0.01 (−0.03, 0.02)
**−0.21 (−0.42, −0.01)**	**−0.10 (−0.18, −0.01)**	**−0.15 (−0.28, −0.02)**	−0.08 (−0.51, 0.36)	−0.05 (−0.29, 0.18)	−0.09 (−0.19, 0.00)	Olive oil	**0.03 (0.01, 0.05)**	−0.02 (−0.04, −0.00)	−0.03 (−0.06, −0.00)	−0.02 (−0.07, 0.02)	−0.00 (−0.04, 0.04)	0.01 (−0.01, 0.04)
−0.16 (−0.38, 0.05)	−0.04 (−0.15, 0.06)	−0.10 (−0.25, 0.04)	−0.03 (−0.46, 0.41)	−0.00 (−0.24, 0.24)	−0.04 (−0.15, 0.06)	0.05 (−0.04, 0.15)	Soybean oil	**−0.05 (−0.07, −0.03)**	**−0.06 (−0.10, −0.03)**	**−0.05 (−0.10, −0.01)**	−0.04 (−0.08, 0.00)	−0.02 (−0.05, 0.00)
**−0.24 (−0.46, −0.03)**	**−0.13 (−0.21, −0.04)**	**−0.18 (−0.32, −0.05)**	−0.11 (−0.54, 0.33)	−0.08 (−0.32, 0.15)	**−0.13 (−0.24, −0.02)**	−0.03 (−0.12, 0.05)	−0.08 (−0.17, 0.01)	Palm oil	−0.01 (−0.04, 0.02)	−0.00 (−0.05, 0.05)	0.02 (−0.02, 0.05)	0.03 (0.00, 0.06)
**−0.31 (−0.48, −0.14)**	**−0.19 (−0.35, −0.04)**	**−0.25 (−0.43, −0.07)**	−0.17 (−0.62, 0.28)	−0.15 (−0.41, 0.11)	**−0.19 (−0.35, −0.03)**	−0.10 (−0.24, 0.05)	−0.15 (−0.30, 0.01)	−0.07 (−0.22, 0.09)	Coconut oil	0.01 (−0.03, 0.05)	0.03 (−0.02, 0.07)	**0.04 (0.01, 0.08)**
**−0.24 (−0.43, −0.04)**	−0.12 (−0.31, 0.08)	−0.18 (−0.39, 0.04)	−0.10 (−0.56, 0.37)	−0.07 (−0.36, 0.21)	−0.12 (−0.31, 0.08)	−0.02 (−0.21, 0.17)	−0.07 (−0.27, 0.13)	0.01 (−0.18, 0.20)	0.07 (−0.10, 0.25)	Beef fat	0.02 (−0.04, 0.07)	0.03 (−0.02, 0.08)
**−0.42 (−0.71, −0.12)**	**−0.30 (−0.52, −0.08)**	**−0.36 (−0.60, −0.11)**	−0.28 (−0.76, 0.20)	−0.25 (−0.56, 0.06)	**−0.30 (−0.52, −0.07)**	−0.20 (−0.42, 0.01)	**−0.25 (−0.47, −0.04)**	−0.17 (−0.38, 0.04)	−0.11 (−0.36, 0.15)	−0.18 (−0.46, 0.10)	Lard	0.01 (−0.03, 0.06)
**−0.49 (−0.71, −0.27)**	**−0.37 (−0.49, −0.25)**	**−0.43 (−0.59, −0.27)**	−0.35 (−0.80, 0.09)	**−0.33 (−0.58, −0.08)**	**−0.37 (−0.49, −0.25)**	**−0.28 (−0.38, −0.17)**	**−0.33 (−0.44, −0.22)**	**−0.25 (−0.36, −0.13)**	**−0.18 (−0.34, −0.02)**	**−0.26 (−0.46, −0.05)**	−0.08 (−0.31, 0.15)	Butter
TC (mmol/l)												

The values above the oils/solid fats correspond to the difference in mean (per 10% isocaloric exchange) (95% CI) in HDL-C (mmol/l) between the row and columns (e.g., the mean difference in average HDL-C between safflower oil and butter is −0.05 mmol/l). The values below the oils/solid fats correspond to the difference in mean in TC (mmol/l) between the column and the row (e.g., the mean difference in average TC between safflower oil and butter is −0.49 mmol/l). Bold ndicates significant effects (95% confidence interval does not overlap zero).

### Primary outcome

#### LDL-C.

**Fig. 3.**
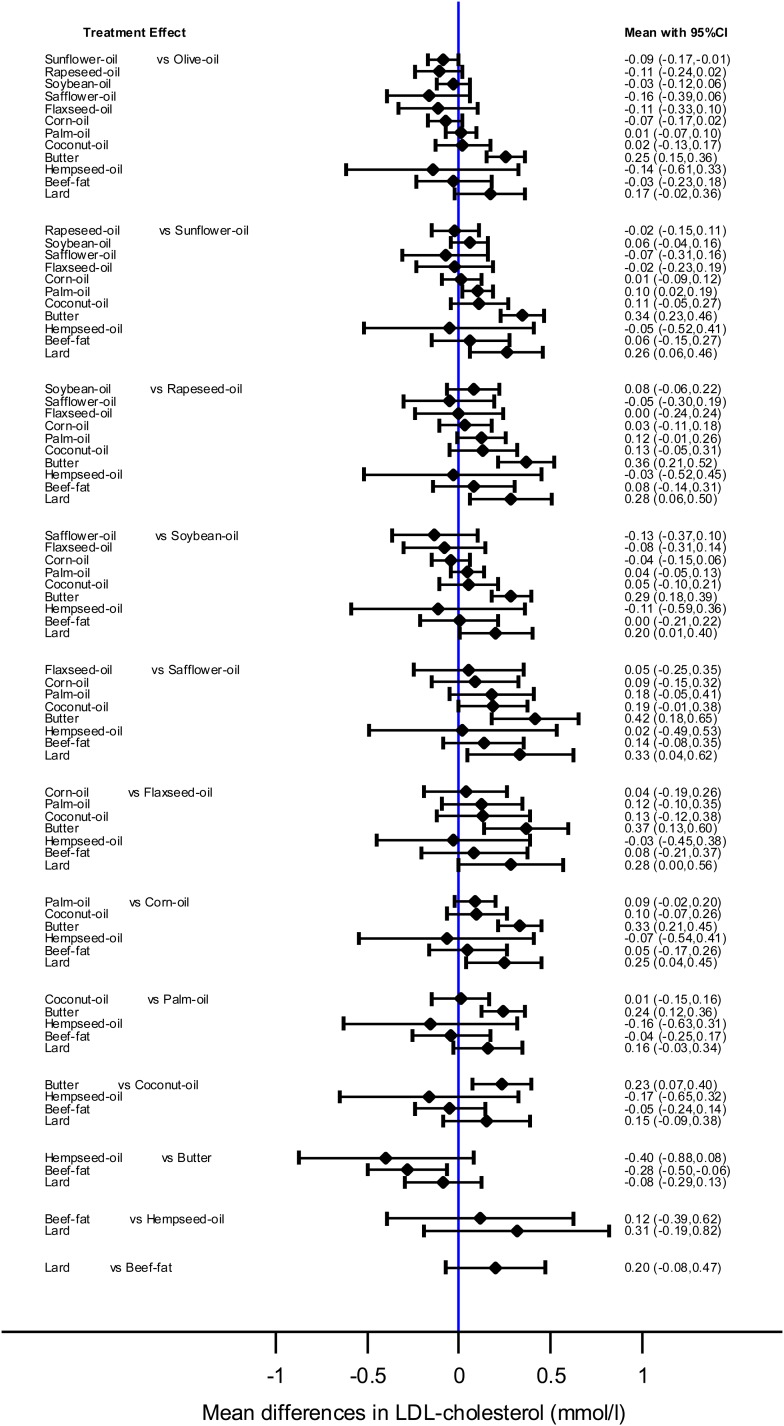
Interval-plot showing the mean differences (95% CI) for LDL-C as estimated from the NMA for every possible pair of interventions. Solid lines represent 95% CIs.

Each 10% of dietary energy from butter replaced with an equivalent amount of safflower, sunflower, rapeseed, flaxseed, corn, olive, soybean, palm, and coconut oil, and beef fat was more effective in reducing LDL-C (−0.42 to −0.23 mmol/l). Safflower, sunflower, rapeseed, corn, and soybean oil had a more pronounced effect on LDL-C when compared with lard (−0.33 to −0.20 mmol/l). Moreover, sunflower oil was more effective in reducing LDL-C than olive and palm oil (−0.10 to −0.09 mmol/l) (**Fig. 3**).

### Secondary outcomes

#### TC.

Likewise to LDL-C, each 10% of dietary energy from butter replaced with an equivalent amount of safflower, sunflower, rapeseed, flaxseed, corn, olive, soybean, palm, and coconut oil, and beef fat was more effective in reducing TC (−0.49 to −0.18 mmol/l). Safflower, sunflower, rapeseed, corn, and soybean oil were more potent to improve TC in comparison to lard (−0.42 to −0.25 mmol/l). In addition, safflower, sunflower, rapeseed, and corn oil resulted in stronger decreases in TC when compared with palm and coconut oil (−0.31 to −0.13 mmol/l), while safflower, sunflower, and rapeseed oil were more effective in reducing TC compared with olive oil (−0.21 to −0.10 mmol/l).

#### HDL-C.

Each 10% of dietary energy from safflower oil replaced with an equivalent amount of sunflower, olive, palm, and coconut oil increased HDL-C levels (0.06–0.09 mmol/l). Sunflower and olive oil were more effective compared with soybean oil (0.03–0.04 mmol/l). Beef fat was also more effective to improve HDL-C compared with safflower and soybean oil (0.05–0.08 mmol/l). In addition, interventions with coconut or palm oil resulted in significantly more elevated HDL-C values as compared with corn and soybean oil (0.04–0.06 mmol/l).

#### TGs.

Each 10% of dietary energy from butter replaced with an equivalent amount of sunflower, soybean, and palm oil were more effective in reducing TGs (−0.06 to −0.04 mmol/l), while safflower, sunflower, corn, soybean, and palm oil were more powerful to reduce TGs when compared with beef fat (−0.09 to −0.08 mmol/l), respectively.

#### SUCRA and rankings.

Safflower oil had the highest SUCRA value for decreases in LDL-C (82%) and TC (90%), followed by rapeseed oil (76% for LDL-C, 85% for TC), and sunflower oil (71% for LDL-C, 72% for TC); whereas, palm oil (74%) had the highest SUCRA value for TG, followed by soybean oil (72%) and safflower oil (68%). Regarding improvements in HDL-C, the following oils were ranked first, second, and third best: coconut oil (88%), palm oil (80%), and beef fat (74%), respectively (supplemental Tables S8–S11).

#### Inconsistency.

The side-splitting approach suggested significant inconsistency for TC in the comparisons of safflower oil versus flaxseed oil, for LDL-C in the comparisons of soybean oil versus corn and palm oil, and between butter and olive oil, and for HDL-C when comparing sunflower oil versus flaxseed oil and comparing soybean oil versus corn and palm oil. For all other comparisons, no significant inconsistency was observed in the side-splitting approach (supplemental Tables S12–S15). The loop-specific approach showed some important inconsistency in the loops formed by olive, safflower, and flaxseed oil, and beef fat for TC, as well as the loops formed by safflower, flaxseed, and corn oil, and beef fat for TC and LDL-C, and the loops formed by soybean, corn, and coconut oil, and beef fat for LDL-C. For HDL-C, inconsistency was observed for the loop formed by safflower, flaxseed, corn, and coconut oil (supplemental Figs. S7–S10). The design-by-treatment model showed no significant inconsistency for TC (*P* = 0.77), LDL-C (*P* = 0.88), HDL-C (*P* = 0.43), and TG (*P* = 0.99).

#### Sensitivity analysis.

In the sensitivity analyses including only low risk of bias trials (n = 33), trials where oils/solid fats were provided by investigators (n = 50), and trials with healthy participants (n = 23), the results of the primary analysis could be confirmed (supplemental Tables S16–S27). Due to missing comparisons, we were not able to conduct NMA sensitivity analyses in at-risk participants and the types of underlying diet (provided by investigators vs. simply advised).

#### Small study effects.

The comparison-adjusted funnel plots for all outcomes appear slightly and/or moderately asymmetric (supplemental Figs. S11–S14).

#### Quality of evidence.

The quality of evidence for TC was rated moderate for most of the comparisons summing up mixed evidence (direct and indirect evidence); whereas for indirect evidence comparisons, the credibility of evidence was mainly rated low (supplemental Table S28). The quality of evidence for LDL-C was rated low or moderate for most of the comparisons summing up mixed evidence; whereas for indirect evidence comparisons, the credibility of evidence was mainly rated low or very low (supplemental Table S29). The credibility of evidence for HDL-C was mainly judged as moderate (supplemental Table S30). The quality of evidence for TG was rated moderate or high for most of the comparisons summing up mixed evidence; whereas for indirect evidence comparisons, the credibility of evidence was mainly rated moderate (supplemental Table S31). Low quality of evidence judgments were mainly driven by the low number of trials, unclear risk of bias, imprecision, and inconsistency for several comparisons.

## DISCUSSION

This NMA synthesized the direct and indirect evidence on the effects of 13 oils and solid fats (safflower, sunflower, rapeseed, hempseed, flaxseed, corn, olive, soybean, palm, and coconut oil, and beef fat, lard, and butter) on blood lipids (TC, LDL-C, HDL-C, and TG).

In summary, safflower oil showed the highest SUCRA value for reduction in TC and LDL-C followed by rapeseed oil and sunflower oil; soybean oil was the most effective oil to reduce TG, followed by corn oil and palm oil; butter and lard were ranked worst for TC and LDL-C reduction; coconut oil was ranked best to improve HDL-C, followed by palm oil and beef fat. The NMA showed that all vegetable oils were more effective in reducing TC (−0.49 to −0.18 mmol/l) and LDL-C (−0.42 to −0.23 mmol/l) compared with butter. Most of the comparisons derived from mixed evidence were rated as moderate quality of evidence.

### Comparison with other studies

In line with findings from the present NMA, pairwise meta-analyses of intervention trials have shown that n-3- and n-6-rich oils were more effective in reducing TC and LDL-C compared with olive oil (8). Similar to our findings, in a meta-analysis of 28 studies, flaxseed oil was not more effective in reducing TC or LDL-C compared with different vegetable oils (olive, rapeseed, hempseed, safflower, or sunflower oil) (80). Findings from another meta-analysis showed that palm oil significantly increased TC by 0.35 mmol/l and LDL-C by 0.24 mmol/l compared with vegetable oils low in SFAs (9). In line with our findings, palm oil (ranked second best for HDL-C) was more effective to improve HDL-C compared with vegetable oils low in SFAs (9). Comparing palm oil with either MUFA- or PUFA-rich oils resulted in higher levels of LDL-C (by approximately 0.20–0.30 mmol/l) and increased levels of HDL-C (by 0.05 mmol/l) (81). Another comprehensive meta-analysis of 60 feeding trials showed that particular replacement of mixed fat constituting 10% of energy by rapeseed, soybean, or olive oil resulted in reductions of the TC:HDL-C ratio that were significantly more pronounced than the corresponding changes following replacement by butter (10).

Focusing on hard clinical endpoints, in prospective observational studies, dietary linoleic acid intake was inversely associated with CHD risk in a dose-response manner (82); whereas, evidence from a meta-analysis of observational studies suggested an inverse association comparing the top versus bottom third of olive oil intake and risk of CVD (83). Interestingly, a dose-response meta-analysis of 15 cohort studies observed a neutral association between one daily tablespoon (14 g/day) of butter and risk of CVD (84).

In addition to these epidemiological data, a number of intervention trials investigated the effects of variations in fat content/fat quality on cardiovascular risk. A beneficial effect of PUFA-rich vegetable oils was observed in men who already had a heart attack during the 5 year randomized Oslo Diet-Heart Study (85). The approach of the Women’s Health Initiative trial was a general replacement of dietary fat by carbohydrates. Incidence rates of both CHD and CVD were equally pronounced in low fat (20% fat) and control groups after the 5 year intervention as well as the 8 year follow-up period (86). The Lyon Heart Study recruited 605 men who had suffered from acute myocardial infarction. These patients were subjected to either a Mediterranean diet (MedD), including a rapeseed oil-based margarine, or a control diet. The MedD turned out to be protective with respect to cardiovascular mortality as well as nonfatal myocardial infarction (87). The PREDIMED (Prevencion con Dieta Mediterranea) trial investigated a MedD with an additional provision of either extra-virgin olive oil (50 g/day) or tree nuts (30 g/day). The incidence of combined cardiovascular events was lower among those assigned to a MedD supplemented with extra-virgin olive oil or nuts than among those assigned to a lower-fat diet (88).

### Possible explanations

With respect to potential mechanisms of action, the general cholesterol-lowering effects predominantly exerted by vegetable oils in the present study might be due to their fatty acid composition, specifically the contents of n-3 and n-6 PUFAs or MUFAs. In this regard, the findings of the present NMA are in line with the predictive equations of Mensink and colleagues based on fatty acid composition of the fats and oils (10, 89). Synthesizing data of 84 controlled trials in a meta-analysis, the authors observed a strong reduction of LDL-C, TC, and the TC:HDL-C ratio, as well as apolipoprotein B, when carbohydrates constituting 1% of energy were replaced in an isocaloric fashion by PUFAs and MUFAs, respectively. LDL-C predicted equations showed that each 10% of dietary energy from butter replaced by unsaturated fatty rich oils (−0.31 to −0.22 mmol/l) were in line with findings from the NMA (−0.42 to −0.20 mmol/l). Moreover, SFAs (higher in coconut oil or butter) raised HDL-C more than MUFA- or PUFA-rich oils (0.02–0.03 mmol/l), and MUFA resulted in slightly higher HDL than PUFA, suggesting that our findings confirm the knowledge based on fatty acid composition. In contrast to these benefits, lauric acid, myristic acid, and palmitic acid were shown to increase plasma levels of TC and LDL-C in the meta-regression analysis by Mensink (89).

Regarding hard clinical endpoints, the role of specific types of fatty acids is discussed controversially. Increasing consumption of PUFAs in place of SFAs resulted in a reduction in CHD in a meta-analysis of intervention trials by Mozaffarian, Micha, and Wallace (5). However, contradicting results have been reported by Ramsden et al. (6), observing that replacement of SFAs by linoleic acid did not affect CHD mortality. In addition, a multivariable meta-regression analysis comparing MUFAs, PUFAs, and SFAs showed no significant effects on CVD risk (90). Despite several analyses with deviating results, there is overall consensus that replacement of (foods rich in) SFAs by (foods rich in) unsaturated fatty acids lowers the risk of CHD (91).

LDL-C is an established risk factor for the development of CVD. For instance, a meta-analysis of 26 trials reported that every 1 mmol/l reduction in LDL-C plasma levels was associated with a corresponding 20% risk reduction in CHD mortality (92). Thus, the significantly more pronounced decrease in LDL-C following intake of vegetable oils as compared with butter demonstrated in the present systematic review seems to be important in regard to the prevention of cardiovascular events. Concerning HDL-C, the evidence is not clear. Epidemiological studies provide evidence for an inverse association between plasma levels of HDL-C and risk of CVD (93). However, Mendelian randomization studies showed that genetically decreased HDL-C was not associated with an increased risk of myocardial infarction, thereby calling into question a causal association between HDL-C and CVD (94). Against this background, effects of diet on HDL-C concentrations should be interpreted with caution. Still, as a marker of cardiovascular health, changes in HDL-C concentrations need to be included when discussing the effect of oils/solid fats in the diet.

### Strength and limitations

Our systematic review and meta-analysis has several strengths and limitations that need to be addressed. Among the strengths are the application of the NMA methodology, the high number of included studies on oils/solid fats (n = 54 trials), the inclusion of 13 different oils/solid fats, the inclusion of four outcomes (TC, LDL-C, HDL-C, and TG), the a priori published protocol, the risk of bias assessment, inconsistency testing, transitivity analyses, sensitivity analyses, and the quality of evidence judgment.

However, several limitations should also be considered when interpreting the findings of the present NMA. First, most of the evidence came from indirect comparisons, showing important heterogeneity and inconsistency, and wide 95% CIs (imprecision) for several comparisons. The similarity across the included trials was only modest (age of participants, BMI, country), and this could be an important factor for the observed inconsistency for some comparisons. Moreover, only 33 out of 54 trials were rated with a low risk of bias. In accordance with this, the credibility of evidence was rated mainly low for comparisons deriving from indirect evidence and moderate for mixed evidence comparisons. This implicates that further research will provide important evidence on the confidence and likely change the effect estimate. However, sensitivity analyses, *i*) including low risk of bias trials, *ii*) excluding high-risk participants, or *iii*) excluding trials where the oils/solid fats were not provided by investigators, confirmed the findings of the main analysis. Second, the present NMA takes only intermediate biomarkers for CVD risk into account, and, as shown for HDL-C, a causal link should be interpreted cautiously.

## CONCLUSIONS

Unsaturated fatty rich oils like safflower, sunflower, rapeseed, flaxseed, corn, olive, soybean, palm, and coconut oil were more effective in reducing LDL-C (−0.42 to −0.20 mmol/l) as compared with SFA-rich food like butter or lard. LDL-C predicted differences based on their fatty acid composition showed that each 10% of dietary energy from butter replaced by unsaturated fatty rich oils (−0.31 to -0.22 mmol/l) were in line with findings from the NMA. Despite the limitations of the NMA approach and the overall low quality of evidence judgements, the NMA findings are in line with existing evidence on the metabolic effects of fat, and support current recommendations to replace high saturated-fat food with unsaturated oils.

## Supplementary Material

Supplemental Data
