# Supplementary material for: Effects of oils and solid fats on blood lipids: a systematic review and network meta-analysis
Source: J Lipid Res. 2018 Jul 13;59(9):1771–82. doi: 10.1194/jlr.P085522 (PMC6121943; doi:10.1194/jlr.P085522)
Supplement: Supplemental Data [file supp_59_9_1771__index.html]

Effects of oils and solid fats on blood lipids: a systematic review and network meta-analysis — Effects of oils and solid fats on blood lipids: a systematic review and network meta-analysis — Supplemental Data 

# Effects of oils and solid fats on blood lipids: a systematic review and network meta-analysis

## Supplemental Data

- Supplementa Data R2 (.pdf, 2.0 MB) - Supplementa Data R2
