# Supplementary material for: Effects of oils and solid fats on blood lipids: a systematic review and network meta-analysis
Source: J Lipid Res. 2018 Jul 13;59(9):1771–82. doi: 10.1194/jlr.P085522 (PMC6121943; doi:10.1194/jlr.P085522)
Supplement: Supplemental Data [file 10.1194_P085522_jlr.P085522-1.pdf]

Supplemental Data

Effects of oils and solid fats on blood lipids: a systematic review and network meta-analysis

Lukas Schwingshackl[1,2], Berit Bogensberger[3], Aleksander Benčič[1], Sven Knüppel[1], Heiner Boeing[1,2], Georg Hoffmann[3]

[1] Department of Epidemiology, German Institute of Human Nutrition Potsdam-Rehbruecke (DIfE), Arthur-Scheunert-Allee 114-116, 14558 Nuthetal, Germany

[2] NutriAct – Competence Cluster Nutrition Research Berlin-Potsdam

[3] Department of Nutritional Sciences, University of Vienna, Althanstrasse 14, 1090 Vienna, Austria

Corresponding author: Lukas Schwingshackl, PhD

Arthur-Scheunert-Allee 114-116; 14558 Nuthetal, Germany

T: +49 (0)33200 88-2723

lukas.schwingshackl@dife.de

Email: a1108642@unet.univie.ac.at

aleksander.bencic@gmail.com

sven.knueppel@dife.de

boeing@dife.de

georg.hoffmann@univie.ac.at

**Content**	**Page**
Supplemental Appendix S1: Search Strategy	3
Supplemental Figure S1: Flow diagram	4
Supplemental Figure S2: Risk of bias	5
Supplemental Table S1: Study characteristics	6-18
Supplemental Table S2: Post-intervention values	19-23
Supplemental Table S3: Fatty acid composition	24
Supplemental Table S4-S7: Contribution matrix	25-28
Supplemental Figure S3-S6: Transitivity analysis	29-32
Supplemental Table S8-S11: SUCRA	33-34
Supplemental Figure S7-S10: Loop-specific approach	35-38
Supplemental Table S12-S15: Side-splitting approach	39-42
Supplemental Table S16-S27: Sensitivity analyses	43-54
Supplemental Figure S11-S14: Comparison-adjusted funnel plots	55-58
Supplemental Table S28-S31: GRADE quality of evidence	59-70

Supplemental Appendix S1:

Full search strategy PubMed:

("Fatty Acids, Omega-6"[MeSH] OR "Fatty Acids, Omega-3"[MeSH] OR "Fatty Acids, Unsaturated"[MeSH] OR "Fatty Acids, Monounsaturated"[MeSH] OR "Trans Fatty Acids"[MeSH] OR "monounsaturated"[tiab] OR "mono-unsaturated"[tiab] OR "unsaturated"[tiab] OR "polyunsaturated"[tiab] OR "saturated"[tiab] OR "trans-unsaturated"[tiab] OR "trans-fatty"[tiab] OR "trans fatty"[tiab] OR "trans unsaturated"[tiab] OR "trans fat"[tiab] OR "omega-6"[tiab] OR "omega-3"[tiab] OR "n-6"[tiab] OR "n-3"[tiab] OR "low-fat"[tiab] OR "high-fat"[tiab] OR "high-carbohydrate"[tiab] OR "low-carbohydrate"[tiab] OR "high-protein"[tiab] OR "low-protein"[tiab] OR "isocaloric"[tiab] OR "palmitic"[tiab] OR "palmitate"[tiab] OR "stearic"[tiab] OR "stearate"[tiab] OR "myristic"[tiab] OR "myristate"[tiab] OR "lauric"[tiab] OR "laurate"[tiab] OR "SFA"[tiab] OR "oleic"[tiab] OR "oleate"[tiab] OR "palmitoleic"[tiab] OR "palmitoleate"[tiab] OR "MUFA"[tiab] OR "linoleic"[tiab] OR "linoleate"[tiab] OR "octadecadienoic acid"[tiab] OR "PUFA"[tiab] OR "vaccenic acid"[tiab] OR "vaccenate"[tiab] OR "conjugated linoleic"[tiab] OR "CLA"[tiab] OR "TFA"[tiab] OR "coconut oil"[tiab] OR "butter"[tiab] OR "lard"[tiab] OR "seed oil"[tiab] OR "safflower oil"[tiab] OR "sunflower oil"[tiab] OR "hempseed oil"[tiab] OR "corn oil"[tiab] OR "sesame oil"[tiab] OR "soybean oil"[tiab] OR "soyabean oil"[tiab] OR "rapeseed oil"[tiab] OR "canola oil"[tiab] OR "olive oil"[tiab] OR "nut oil"[tiab] OR "linseed oil"[tiab] OR "flaxseed oil"[tiab] OR "grapeseed oil"[tiab] OR "peanut oil"[tiab] OR "avocado oil"[tiab] OR "palm oil"[tiab] OR "vegetable oil"[tiab] OR "margarine"[tiab] OR "hydrogenated oil"[tiab]) AND ("cholesterol*"[MeSH] OR "lipid*"[MeSH] OR "lipoprotein*"[tiab] OR "high density"[tiab] OR "low density"[tiab] OR "triacylglycerol*"[tiab] OR "LDL"[tiab] OR "HDL"[tiab]) AND ("randomized"[tiab] OR "intervention"[tiab] OR "ward"[tiab] OR "feeding"[tiab] OR "trials"[tiab] OR "trial"[tiab] OR "supplements"[tiab] OR "supplement"[tiab] OR "supplementation"[tiab]) NOT ("Case-Control Studies"[MeSH] OR "Cohort Studies"[MeSH] OR "case-control"[tiab] OR "cohort"[tiab] OR "case-report"[tiab] OR "ad libitum"[tiab] OR "adolescents"[All Fields] OR "children"[All Fields] OR "gestational"[tiab] OR "pregnant"[tiab] OR "pregnancy"[tiab]) NOT ("rats"[tiab] OR "monkeys"[tiab] OR "primates"[tiab] OR "rabbits"[tiab] OR "cats"[tiab] OR "dogs"[tiab] OR "mice"[tiab] OR "pigs"[tiab] OR "cows"[tiab]) AND ((Randomized Controlled Trial[ptyp] OR Clinical Trial[ptyp]) AND "humans"[MeSH Terms] AND "adult"[MeSH Terms])

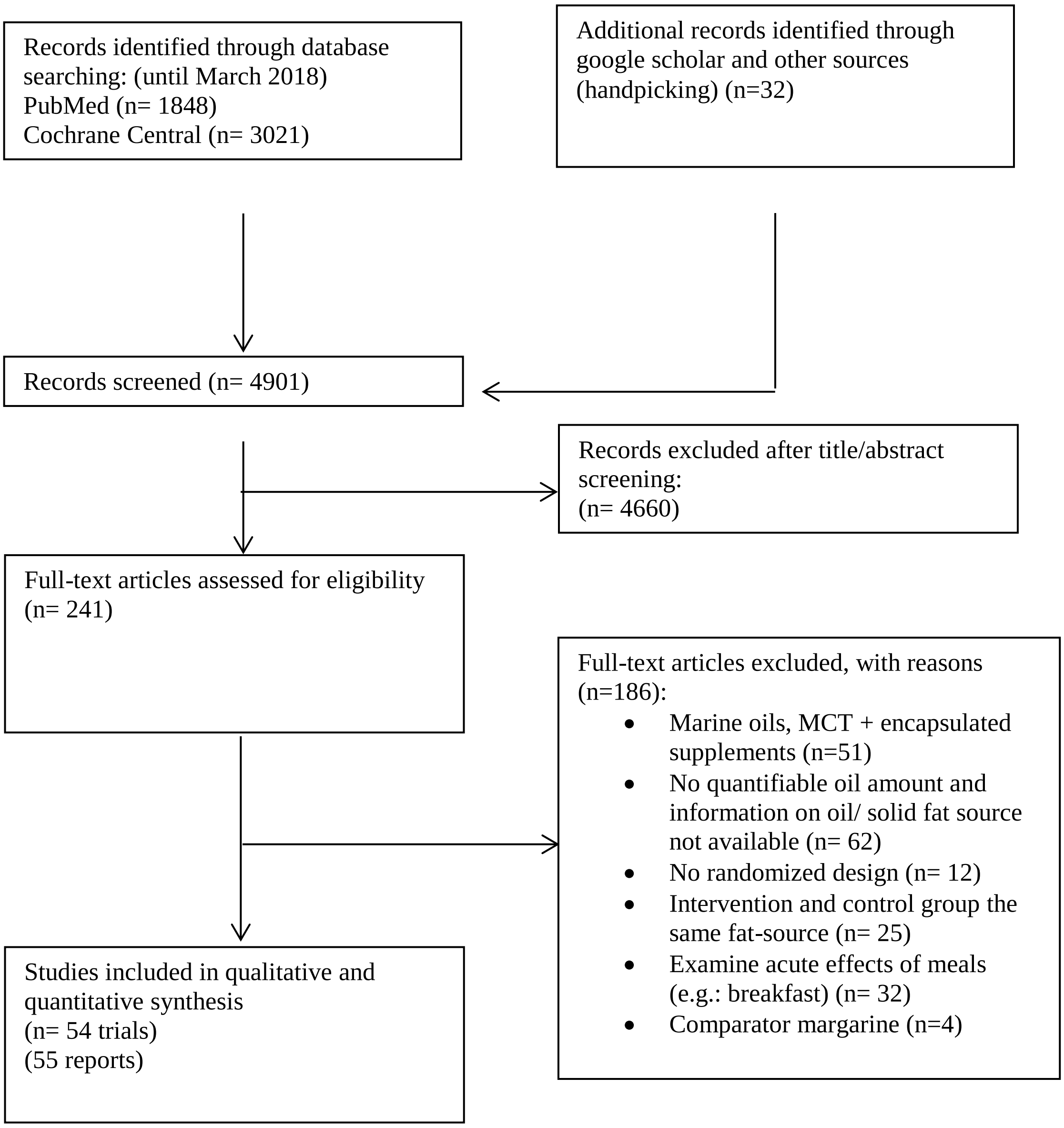

Supplemental Figure S1: Flow diagram of study selection; MCT = medium-chain triglycerides

Supplemental Figure S2: Risk of bias as percentages across all included studies.

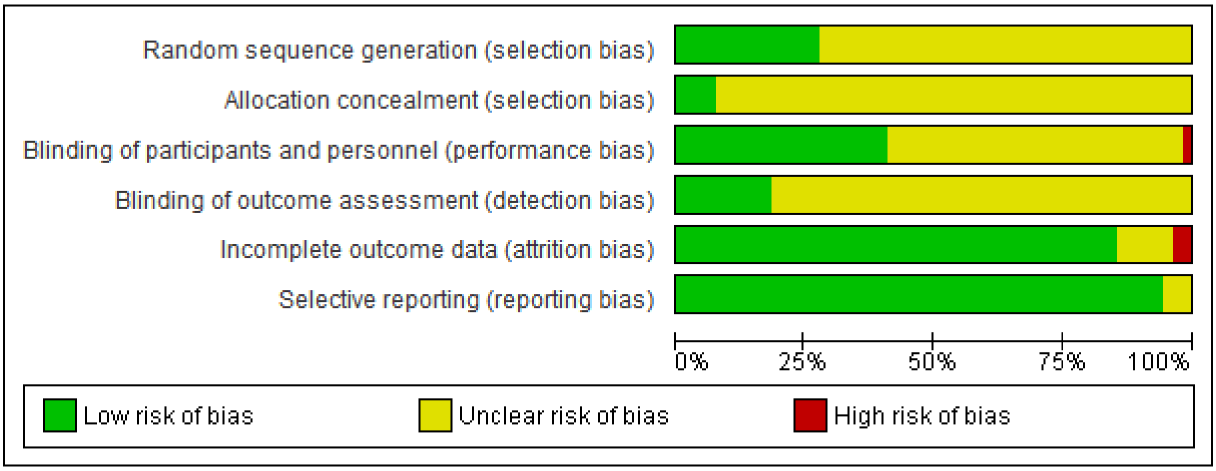

Reference	Country	Study Design	Comparison Oils/Solid fat	Sample size (completers) Disease status	Mean age	Mean baseline BMI	Type 2 diabetes (%)	Female (%)	Duration, weeks	Arm 1	Arm 2	Arm 3	Type of diet	Primary outcome of the study	Outcomes	Conflict of interest
Aguilera, 2004	Spain	RCT, parallel	Olive oil vs. Sunflower oil	20 peripheral vascular disease	65	26.7	0	0	16	Olive oil (8 g/d)	Sunflower oil (8 g/d)	NR	Healthy diet advice<br><br>Oils were provided by investigators<br><br>Weight loss: none	LDL-oxidation susceptibilit y	TC, HDL-C, LDL-C, TG	Ministerio de Education y Ciencia. Instituto Danone
Akrami, 2017	Iran	RCT, parallel	Flaxseed oil vs. Sunflower oil	52 Metabolic syndrome	48.5	NR	0	37	7	Flaxseed oil (25 ml/d)	Sunflower oil (25 ml/d)	NR	Healthy diet advice<br><br>Oils were advised<br><br>Weight loss: yes, equally pronounced in both arms	NR	TC, HDL-C, LDL-C, TG	No conflict of interest
Assuncao, 2009	Brazil	RCT, parallel	Coconut oil vs. Soybean oil	40 abdominal obesity	28.5	31.1	0	100	12	Coconut oil (30 ml/d)	Soybean oil (30 ml/d)	NR	Healthy diet advice<br><br>Oils were provided by investigators<br><br>Weight loss: yes, in both arms	NR	TC, HDL-C, LDL-C, TG	Not reported
Baudet, 1984	France	RCT, crossover	Palm oil vs. Sunflower oil	24 50% hyper-cholesterol-emia	45.9	NR	0	100	20	Palm oil (45 g/d)	Sunflower oil (45 g/d)	NR	Healthy diet advice<br><br>Oils were advised<br><br>Weight loss: NR	NR	TC, TG	International Olive Council
Binkoski, 2005	USA	RCT, crossover	Olive oil vs. Sunflower oil	31 moderate hyper-cholesterol-emia	46.2	26.1	0	61	4	Olive oil (15% of total energy)	Sunflower oil (15% of total energy)	NR	Healthy diet by investigators<br><br>Oils were	"Main outcome measures": lipid and lipoprotein	TC, HDL-C, LDL-C, TG	Grant from the National Sunflower Association

													provided by investigators<br><br>Weight loss: none	levels and measures of oxidative stress		
Brassard, 2017	Canada	RCT, crossover	Butter vs. Corn oil vs. Olive oil	92 abdominal obesity and relatively low HDL-cholesterol	38	30.5	0	53	4	Butter (48.9 g/2500 kcal)	Corn oil (48.9 g/2500 kcal)	Olive oil (48.9 g/2500 kcal)	Healthy diet by investigators<br><br>Oils and solid fats were provided by investigators<br><br>Weight loss: none	HDL-C	TC, HDL-C, LDL-C, TG	Dairy Research Cluster Initiative (Agriculture and Agri-Food Canada, Dairy Farmers of Canada, the Canadian Dairy Network, and the Canadian Dairy Commission
Candido, 2017	Brazil	RCT, parallel	Olive oil vs. Soybean oil	41 excess body fat	27	30.1	0	100	9	Olive oil (25 ml/d)	Soybean oil (25 ml/d)	NR	Habitual diet advice<br><br>Oils were provided by investigators<br><br>Weight loss: yes, equally pronounced in both arms	NR	TC, HDL-C, LDL-C, TG	No conflict of interest
Cater, 1997	USA	RCT, crossover	Palm oil vs. Sunflower oil	9 mild hyper-cholesterol-emia	66	27	NR	0	3	Palm oil (43% of total energy)	Sunflower oil (43% of total energy)	NR	High-fat diet by investigators<br><br>Oils were provided by investigators<br><br>Weight loss: none	NR	TC, HDL-C, LDL-C, TG	NIH, NIH-NHLBI
Cater, 2001	USA	RCT, crossover	Palm oil vs. Sunflower oil	7 mild hyper-cholesterol-emia	66	27	NR	0	3	Palm oil (43% of total energy)	Sunflower oil (43% of total energy)	NR	High-fat diet by investigators<br><br>Oils were	NR	TC, HDL-C, LDL-C, TG	NIH, NIH-NHLBI

													provided by investigators<br><br>Weight loss: none			
Chang, 2017	Malaysia	RCT, crossover	Palm oil vs. Sunflower oil	47 abdominal obesity	32.8	28.7	0	75	6	Palm oil (49 g/d)	Sunflower oil (49 g/d)	NR	Habitual diet by investigators<br><br>Oils and solid fats were provided by investigators<br><br>Weight loss: none	Postprandial changes from fasting value in C-peptide	TC, HDL-C, LDL-C, TG	Malaysian Palm Oil Board
Choudhury, 1995	Australia	RCT, crossover	Palm oil vs. Olive oil	21 healthy	27	24	0	52	4	Palm oil (17% of total energy)	Olive oil (17% of total energy)	NR	Healthy diet advice<br><br>Oils were provided by investigators<br><br>Weight loss: none	NR	TC, HDL-C, LDL-C, TG	Malaysian Palm Oil Board
Cicero, 2009	Italy	RCT, parallel	Corn oil vs. Olive oil	22 moderate hyper-cholesterol-emia	50	25.7	0	50	6.5	Corn oil (provided 1 l cans)	Olive oil (provided 1 l cans)	NR	Healthy diet advice<br><br>Oils were advised<br><br>Weight loss: none	NR	TC	Unrestricted grant from Bonomelli Srl
Dittrich, 2015	Germany	RCT,	Flaxseed oil vs. Sunflower oil	59 moderate hyper-triacylgly-ceridemic	61	NR	NR	NR	10	Flaxseed oil (20 g/d)	Sunflower oil (20 g/d)	NR	NR<br><br>Foods were provided by investigators<br><br>Weight loss: none (weight gain in sunflower oil arm)	TG	TC, HDL-C, LDL-C, TG	No conflict of interest
Engel, 2015	Denmark	RCT, crossover	Butter vs.	47 healthy	40.4	23.5	0	70	5	Butter (17 g/d)	Olive oil (13 g/d)	NR	Habitual diet advice	NR	TC, HDL-C, LDL-C,	Danish Dairy Research

			Olive oil										Oils and solid fats were provided by investigators<br><br>Weight loss: none		TG	Foundation. Butter was provided by Arla Foods, Denmark, and olive oil was provided by Aarhus Karlshamn, Denmark A/S
Filippou, 2014	United Kingdom	RCT, crossover	Palm oil vs. Sunflower oil	41 healthy	29.1	23	0	76	6	Palm oil (45 g/d)	Sunflower oil (45 g/d)	NR	Healthy diet by investigators<br><br>Oils were provided by investigators<br><br>Weight loss: none	Change in the postprandial increment in C-peptide	TC, HDL-C, LDL-C, TG	Malaysian Palm Oil Board
Han, 2002	USA	RCT, crossover	Butter vs. Soybean oil	19 moderately elevated LDL cholesterol levels	64.7	28.8	0	58	4.5	Butter (20% of total energy)	Soybean oil (20% of total energy)	NR	Healthy diet (soybean arm) by investigators<br><br>Oils and fats were provided by investigators<br><br>Weight loss: NR	NR	TC, HDL-C, LDL-C, TG	Not reported
Harris, 2017	USA	RCT, crossover	Coconut oil vs. Safflower oil	12 healthy	58.8	26.4	0	100	4	Coconut oil (30 ml/d)	Safflower oil (30 ml/d)	NR	NR<br><br>Oils were provided by investigators<br><br>Weight loss: none	NR	TC, HDL-C, LDL-C, TG	No conflict of interest
Iggman, 2015	Sweden	RCT, parallel	Palm oil vs. Sunflower oil	39 healthy	26.9	20.2	0	31	7	Palm oil (12% of total energy)	Sunflower oil (12% of total energy)	NR	Habitual diet advice<br><br>Foods were provided by investigators	Liver fat content and body composition , measured with	TC, HDL-C, LDL-C	No conflict of interest

													Weight loss: none	magnetic resonance imaging		
Insull, 1994	USA	RCT, crossover	Corn oil vs. Soybean oil vs. Sunflower oil	61 healthy	34.2	NA	NR	57	5	Corn oil (22-26 % of total energy)	Soybean oil (22-26 % of total energy)	Sunflower oil (22-26 % of total energy)	Habitual diet advice<br><br>Oils were provided by investigators<br><br>Weight loss: none	NR	TC, HDL-C, LDL-C	No conflict of interest
Karvonen, 2002	Finnland	RCT, parallel	Olive oil vs. Rapeseed oil	45 mild hyper-cholesterol-emia	51.3	25.7	0	60	6	Olive oil (30 g/d)	Rapeseed oil (30 g/d)	NR	Habitual diet advice<br><br>Oils were provided by investigators<br><br>Weight loss: none	NR	TC, HDL-C, LDL-C, TG	Camelina Ltd
Kawakami, 2015	Japan	RCT, crossover	Corn oil vs. Flaxseed oil	15 healthy	44.5	25.1	0	0	12	Corn oil (10 g/d)	Flaxseed oil (10 g/d)	NR	Habitual diet advice<br><br>Oils were advised<br><br>Weight loss: none	NR	TC, HDL-C, LDL-C, TG	Supported by Sadamitsu food industry limited
Khaw, 2018	United Kingdom	RCT, parallel	Butter vs. Coconut oil vs. Olive oil	91 healthy	59.9	25.1	0	69	4	Butter (50 g/d)	Coconut oil (50 g/d)	Olive oil (50 g/d)	Habitual diet advice<br><br>Oils were provided by investigators<br><br>Weight loss: none	Change in serum LDL-C	TC, HDL-C, LDL-C, TG	No conflict of interest
Kontogianni, 2013	Greece	RCT, crossover	Flaxseed oil vs. Olive oil	37 healthy	25.6	21.9	0	78	6	Flaxseed oil (15 ml/d)	Olive oil (15 ml/d)	NR	Habitual diet advice<br><br>Oils were provided by investigators<br><br>Weight loss:	"Sample size calculation was performed based on expected changes in	TC, HDL-C, LDL-C, TG	Minerva S.A. for the supply of the extra virgin olive oil

													none	adiponectin, HDL cholesterol and triglycerides levels after the flaxseed intervention ”		
Kris-Etherton, 1993	USA	RCT, crossover	Butter vs. Olive oil vs. Sunflower oil	18 healthy	26	23	0	0	4	Butter (30% of total energy)	Olive oil (30% of total energy)	Soybean oil (30% of total energy)	High-fat diet by investigators<br><br>Foods were provided by investigators<br><br>Weight loss: NR	NR	TC, HDL-C, LDL-C, TG	American Cocoa Research Institute
Kruse, 2015	Germany	RCT, parallel	Olive oil vs. Rapeseed oil	18 obese	55	29.5	0	0	4	Olive oil (50 g/d)	Rapeseed oil (50 g/d)	NR	Habitual diet advice<br><br>Oils were provided by investigators<br><br>Weight loss: none	Body composition, serum lipids, serum liver enzymes, and inflammatory gene expression in subcutaneous (s. c.) adipose tissue	TC, HDL-C, LDL-C, TG	Union zur Förderung von Öl- und Proteinpflanzen
Lichtenstein, 1993<br>Schwab, 1998	USA	RCT, crossover	Beef tallow vs. Corn oil vs. Olive oil vs. Rapeseed oil	15 elevated LDL cholesterol levels	61	27.4	0	53	4	Beef tallow (20% of total energy)	Corn oil (20% of total energy)	Olive oil (20% of total energy)<br>Rape-seed oil (20% of total energy)	Healthy diet by investigators<br><br>Oils were provided by investigators<br><br>Weight loss: none	NR	TC, HDL-C, LDL-C, TG	Grant from Uncle Bens
Lichtenstein, 1999	USA	RCT, crossover	Butter vs.	36 elevated	63	27.4	0	50	5	Butter (20% of	Soybean oil (20% of	NR	Healthy diet (soybean arm)	NR	TC, HDL-C, LDL-C,	Not reported

			Soybean oil	LDL cholesterol levels						total energy)	total energy)		by investigators<br><br>Oils and solid fats were provided by investigators<br><br>Weight loss: none		TG	
Lucci, 2016	Italy	RCT, parallel	Olive oil vs. Palm oil	145 hyper-cholesterol-emia	63.5	28.3	NR	92	12	Olive oil (25 ml/d)	Palm oil (25 ml/d)	NR	Healthy diet advice<br><br>Oils were provided by investigators<br><br>Weight loss: none (measured as BMI)	NR	TC, HDL-C, LDL-C, TG	Not reported
Maki, 2015	USA	RCT, crossover	Corn oil vs. Olive oil	54 hyper-cholesterol-emia	53.8	28.2	0	65	3	Corn oil (54 g/d)	Olive oil (54 g/d)	NR	Habitual diet by investigators<br><br>Oils and foods were provided by investigators<br><br>Weight loss: none	Change in LDL-C	TC, HDL-C, LDL-C, TG	funded by ACH Food Companies
Morillas-Ruiz, 2014	Spain	RCT, crossover	Butter vs. Olive oil	53 hyper-cholesterol-emia	63.5	27.8	13	100	4	Butter (20 g/d)	Olive oil (20 g/d)	NR	Habitual diet advice<br><br>Oils and solid fats were provided by investigators<br><br>Weight loss: none (measured as BMI)	NR	TC, HDL-C, LDL-C, TG	No conflict of interest
Ng, 1992	Malaysia	RCT, crossover	Olive oil vs.	33 healthy	28.7	22	NR	39	6	Olive oil (23% of	Palm oil (23% of	NR	Habitual diet by	NR	TC, HDL-C, LDL-C,	Palm Oil Research Institute

			Palm oil							total energy)	total energy)		investigators<br><br>Oils were provided by investigators<br><br>Weight loss: none		TG	of Malaysia
Nigam, 2014	India	RCT, parallel	Olive oil vs. Rapeseed oil	93 Non-alcoholic fatty live disease	37.1	27.3	0	0	27	Olive oil (20 g/d)	Rapeseed oil (20 g/d)	NR	Healthy diet advice<br><br>Oils were provided by investigators<br><br>Weight loss: yes, only in Olive oil arm	NR	HDL-C, TG	No conflict of interest
Paschos, 2007	Greece	RCT, parallel	Flaxseed oil vs. Safflower oil	35 hyper-cholesterol-emia	51.4	28	0	0	12	Flaxseed oil (15 ml/d)	Safflower oil (15 ml/d)	NR	Habitual diet advice<br><br>Oils were provided by investigators<br><br>Weight loss: none	NR	TC, HDL-C, TG	Not reported
Pedersen, 2000	Denmark	RCT, crossover	Olive oil vs. Rapeseed oil vs. Sunflower oil	18 healthy	24	23	0	0	3	Olive oil (18% of total energy)	Rapeseed oil (18% of total energy)	Sunflower oil (18% of total energy)	Habitual diet by investigators<br><br>Oils were provided by investigators<br><br>Weight loss: none	TC, TG for power calculation	TC, HDL-C, LDL-C, TG	Danish Food Technology Research Program
Perez-Jimenez, 1995	Spain	RCT, crossover	Olive oil vs. Sunflower oil	21 healthy	23	24.7	0	0	4	Olive oil (40% fat of total energy)	Sunflower oil (40% fat of total energy)	NR	High-fat diet by investigators<br><br>Oils were provided by investigators<br><br>Weight loss:	NR	TC, HDL-C, LDL-C, TG	Not reported

													none			
Perona, 2004	Spain	RCT, crossover	Olive oil vs. Sunflower oil	62 hyper-tensive	84	28.8	0	0	4	Olive oil (60 g/d)	Sunflower oil (60 g/d)	NR	Habitual diet by investigators<br><br>Oils were provided by investigators<br><br>Weight loss: NR	NR	TC, HDL-C, LDL-C, TG	Not reported
Rallidis, 2003	Greece	RCT, parallel	Flaxseed oil vs. Safflower oil	76 hyper-cholesterol-emia	51	28.3	0	0	12	Flaxseed oil (15 ml/d)	Safflower oil (15 ml/d)	NR	Habitual diet advice<br><br>Oils were provided by investigators<br><br>Weight loss: none (measured as BMI)	NR	TC, HDL-C, LDL-C, TG	Not reported
Reiser, 1985	USA	RCT, crossover	Beef fat vs. Coconut oil vs. Safflower oil	19 healthy	25.6	NR	0	0	5	Beef fat (21% of total energy)	Coconut oil (21% of total energy)	Safflower oil (21% of total energy)	Test diet by investigators<br><br>Oils and solid fats were provided by investigators<br><br>Weight loss: none	NR	TC, HDL-C, LDL-C, TG	National Live Stock and Meat Board, the Texas Cattle Feeders Association, and the Standard Meat Co of Fort Worth, Texas.
Salar, 2016	Iran	RCT, parallel	Rapeseed oil vs. Sunflower oil	47 type 2 diabetes	51.4	29.9	100	100	8	Rapeseed oil (30 g/d)	Sunflower oil (30 g/d)	NR	Healthy diet advice<br><br>Oils were provided by investigators<br><br>Weight loss: none	NR	TC, HDL-C, LDL-C, TG	No conflict of interest
Scholtz, 2004	South Africa	RCT, parallel	Palm oil vs. Sunflower	38 hyper-fibrino-	48.3	28.5	0	36	4	Palm oil (12% of total	Palm oil (12% of total	NR	Habitual diet advice	Fibrinogen and serum lipids	TC, HDL-C, LDL-C, TG	Palm Oil Research Institute of Malaysia for

			oil	genemic						energy)	energy)		Oils were provided by investigators<br><br>Weight loss: none			sponsoring the study
Schwab, 1995	Finland	RCT, crossover	Coconut oil vs. Palm oil	15 healthy	23.9	21.4	100	0	4	Coconut oil (4% of total energy)	Palm oil (4% of total energy)	NR	Healthy diet advice<br><br>Oils were provided by investigators<br><br>Weight loss: none	NR	TC, HDL-C, LDL-C, TG	Not reported
Schwab, 2006	Finland	RCT, crossover	Flaxseed oil vs. Hempseed oil	14 healthy	45	24.5	0	43	4	Flaxseed oil (30 ml/d)	Hempseed oil (30 ml/d)	NR	Healthy diet advice<br><br>Oils were provided by investigators<br><br>Weight loss: none	NR	TC, HDL-C, LDL-C, TG	Not reported
Sirtori, 1986	Italy	RCT, crossover	Corn oil vs. Olive oil	23 high athero-sclerotic risk	47.4	NR	50	NR	8	Corn oil (45 g/d)	Olive oil (45 g/d)	NR	Habitual diet advice<br><br>Oils were provided by investigators<br><br>Weight loss: none	NR	TC, HDL-C, LDL-C, TG	Grant from the European Economic Community
Sirtori, 1992	Italy	RCT, crossover	Corn oil vs. Olive oil	12 hyper-cholesterol-emia	NA	NR	NR	NR	6	Corn oil (27-30% fat of total energy)	Olive oil (27-30% fat of total energy)	NR	Habitual diet advice<br><br>Oils were provided by investigators<br><br>Weight loss: none	NR	TC, HDL-C, LDL-C, TG	Grant from the European Economic Community
Stricker, 2008	Switzer-land	RCT, parallel	Rapeseed oil vs. Sunflower oil	40 peripheral artery disease	65.2	NR	33	13	8	Rapeseed oil (2 tablespoons/	Sunflower oil (2 tablespoons/	NR	Habitual diet advice<br><br>Oils were	NR	TC, HDL-C, LDL-C, TG	partly funded by the Fondo Balli

										d)	d)		provided by investigators<br><br>Weight loss: NR			
Sundram, 1995	Malaysia	RCT, crossover	Palm oil vs. Rapeseed oil	23 healthy	22	21.3	NR	0	4	Palm oil (20% energy intake)	Rapeseed oil (20% energy intake)	NR	Habitual diet by investigators<br><br>Oils were provided by investigators<br><br>Weight loss: none (measured as BMI)	NR	TC, HDL-C, LDL-C, TG	Palm Oil Research Institute of Malaysia
Sundram, 2007	Malaysia	RCT, crossover	Palm oil vs. Soybean oil	30 healthy	30	22	65.6	0	4	Palm oil (22% energy intake)	Soybean oil (22% energy intake)	NR	Habitual diet by investigators<br><br>Oils were provided by investigators<br><br>Weight loss: none	NR	TC, HDL-C, LDL-C, TG	Palm Oil Research Institute of Malaysia
Teng, 2010	Malaysia	RCT, crossover	Palm oil vs. Soybean oil	41 healthy	28.8	21.9	80	0	5	Palm oil (54 g/d)	Soybean oil (54 g/d)	NR	Habitual diet by investigators<br><br>Oils were provided by investigators<br><br>Weight loss: none	LDL-C for power calculation	TC, HDL-C, LDL-C, TG	Malaysian Palm Oil Board
Tholstrup, 2011	Denmark	RCT, crossover	Lard vs. Palm oil vs. Olive oil	43 healthy	29.6	22.9	0	0	3	Lard (17% of total energy)	Palm oil (17% of total energy)	Olive oil (17% of total energy)	Habitual diet advice<br><br>Oils and fats were provided by investigators<br><br>Weight loss:	NR	TC, HDL-C, LDL-C, TG	Supported by Malaysian Palm Oil Board

													none			
Utarwuthipong , 2009	Thailand	RCT, crossover	Palm oil vs. Soybean oil	16 hyper-cholesterol-emia	44-67	<25	100	0	10	Palm oil (20% of total energy)	Soybean oil (20% of total energy)	NR	Healthy diet advice<br><br>NR<br><br>Weight loss: none	NR	TC, HDL-C, LDL-C, TG	No conflict of interest
Vega-López, 2009	USA	RCT, crossover	Corn oil vs. Soybean oil	30 elevated LDL cholesterol levels	64.2	25.6	100	0	5	Corn oil (17% of total energy)	Soybean oil (17% of total energy)	NR	Habitual diet by investigators<br><br>Oils were provided by investigators<br><br>Weight loss: none	Fasting and non-fasting lipid, lipoprotein, apolipoprotein, and fasting high sensitivity C-reactive protein (hsCRP) concentrations	TC, HDL-C, LDL-C, TG	No conflict of interest
Voon, 2011	Malaysia	RCT, crossover	Coconut oil vs. Olive oil vs. Palm oil	45 healthy	30.1	23.1	80	0	5	Coconut oil (20% of total energy)	Olive oil (20% of total energy)	Palm oil (20% of total energy)	Habitual diet by investigators<br><br>Oils were provided by investigators<br><br>Weight loss: none	Change in total homocysteine	TC, HDL-C, LDL-C, TG	Malaysian Palm Oil Board
Wardlaw, 1990	USA	RCT, crossover	Butter vs. Corn oil vs. Sunflower oil	20 healthy	34.7	NR	0	0	5	Butter (40% fat of total energy)	Corn oil (40% fat of total energy)	Sunflower oil (40% fat of total energy)	High-fat diet by investigators<br><br>Oils were provided by investigators<br><br>Weight loss: none	NR	TC, HDL-C, LDL-C, TG	SVO Enterprises, Inc. Wickliffe, OH.
Zhang, 1997	China	RCT, parallel	Lard Vs. Palm oil	60 healthy	18-25	NR	0	0	6	Lard (30% fat of total	Palm oil (30% fat of total	Soybean oil (30% fat of total	Habitual diet by investigators	NR	TC, HDL-C, LDL-C, TG	Malaysian Palm Oil Promotion Council

			vs.<br>Soybean oil							energy)	energy)	energy)	Oils were provided by investigators<br><br>Weight loss: NR			

Supplemental Table S1: General and specific study characteristics of the included trials investigating the effects of different oils and solid fats on blood lipids; NR, not reported.

Reference	Comparison	TC ± SD (mmol/l)	LDL-C ± SD (mmol/l)	HDL-C ± SD (mmol/l)	TG ± SD(mmol/l)
Aguilera, 2004	Sunflower oil	6.05 ± 0.93	3.83 ± 0.97	1.19 ± 0.54	2.24 ± 0.46
	Olive oil	6.30 ± 1.23	4.42 ± 1.37	1.21 ± 0.37	1.45 ± 0.30
Akrami, 2017	Flaxseed oil	5.10 ± 0.96	3.13 ± 0.70	1.09 ± 0.27	1.77 ± 0.74
	Sunflower oil	4.93 ± 0.86	3.05 ± 0.72	1.13 ± 0.22	1.61 ± 0.85
Assuncao, 2009	Coconut oil	5.13 ± 1.01	3.02 ± 0.95	1.26 ± 0.06	2.03 ± 1.06
	Soybean oil	5.42 ± 0.74	3.47 ± 0.74	1.17 ± 0.15	1.67 ± 0.73
Baudet, 1984	Palm oil	5.12 ± 0.88	NR	NR	0.89 ± 0.58
	Sunflower oil	4.54 ± 0.59	NR	NR	1.23 ± 1.12
Binkoski, 2005	Sunflower oil	5.47 ± 0.78	3.54 ± 0.61	1.32 ± 0.33	1.34 ± 0.61
	Olive oil	5.67 ± 0.78	3.72 ± 0.61	1.34 ± 0.33	1.28 ± 0.61
Brassard, 2017	Butter	5.10 ± 0.95	3.30 ± 0.84	1.11 ± 0.21	1.36 ± 0.73
	Corn oil	4.60 ± 0.81	2.84 ± 0.69	1.10 ± 0.20	1.30 ± 0.62
	Olive oil	4.82 ± 0.89	3.03 ± 0.78	1.10 ± 0.19	1.38 ± 0.67
Candido, 2017	Soybean oil	4.12 ± 0.85	2.36 ± 0.67	1.12 ± 0.27	0.90 ± 0.54
	Olive oil	4.25 ± 0.92	2.48 ± 0.69	1.28 ± 0.32	0.89 ± 0.54
Cater, 1997	Palm oil	5.79 ± 0.72	4.37 ± 0.70	0.91 ± 0.16	1.35 ± 0.42
	Sunflower oil	5.22 ± 0.52	3.72 ± 0.47	0.93 ± 0.26	1.45 ± 0.89
Cater, 2001	Palm oil	5.84 ± 0.70	4.42 ± 0.70	0.88 ± 0.20	1.39 ± 0.50
	Sunflower oil	5.12 ± 0.50	3.70 ± 0.60	0.91 ± 0.20	1.35 ± 0.60
Chang, 2017	Palm oil	4.89 ± 0.73	3.05 ± 0.56	1.23 ± 0.22	1.31 ± 0.73
	Sunflower oil	4.71 ± 0.82	2.89 ± 0.64	1.21 ± 0.23	1.29 ± 0.73
Choudhury, 1995	Palm oil	4.65 ± 1.26	3.33 ± 1.13	0.91 ± 0.33	0.97 ± 0.56
	Olive oil	4.63 ± 0.99	3.41 ± 0.96	0.80 ± 0.19	0.95 ± 0.41
Cicero, 2009	Corn oil	6.35 ± 0.76	NR	NR	NR
	Olive oil	6.00 ± 0.55	NR	NR	NR
Dittrich, 2015	Flaxseed oil	5.38 ± 1.21	3.45 ± 1.09	1.20 ± 0.28	1.87 ± 0.89
	Sunflower oil	5.83 ± 1.37	3.77 ± 1.15	1.29 ± 0.48	1.91 ± 1.06
Engel, 2015	Butter	5.50 ± 0.98	3.04 ± 0.86	1.75 ± 0.41	1.00 ± 0.35

	Olive oil	5.27 ± 0.95	2.87 ± 0.86	1.73 ± 0.39	0.93 ± 0.38
Filippou, 2014	Palm oil	4.99 ± 0.70	3.02 ± 0.65	1.44 ± 0.30	0.96 ± 0.36
	Sunflower oil	4.62 ± 0.64	2.72 ± 0.61	1.39 ± 0.26	0.91 ± 0.36
Han, 2002	Butter	6.66 ± 1.13	4.58 ± 1.00	1.24 ± 0.22	1.73 ± 0.74
	Soybean oil	5.88 ± 0.92	3.89 ± 0.78	1.17 ± 0.22	1.79 ± 0.78
Harris, 2017	Coconut oil	6.16 ± 0.62	3.56 ± 0.70	1.83 ± 0.49	1.21 ± 0.91
	Safflower oil	5.68 ± 0.59	3.28 ± 0.67	1.63 ± 0.38	1.34 ± 1.27
Iggman, 2015	Palm oil	4.20 ± 1.03	2.40 ± 0.80	1.40 ± 0.57	0.56 ± 0.27
	Sunflower oil	4.20 ± 0.87	2.00 ± 1.22	1.40 ± 0.56	0.65 ± 0.37
Insull, 1994	Corn oil	3.93 ± 0.66	2.22 ± 0.58	1.30 ± 0.28	NR
	Soybean oil	4.00 ± 0.68	2.20 ± 0.58	1.36 ± 0.30	NR
	Sunflower oil	3.91 ± 0.73	2.17 ± 0.59	1.32 ± 0.33	NR
Karvonen, 2002	Rapeseed oil	5.70 ± 0.80	NR	NR	NR
	Olive oil	6.00 ± 0.80	NR	NR	NR
Kawakami, 2015	Corn oil	5.62 ± 0.77	3.57 ± 0.77	1.42 ± 0.31	1.44 ± 0.74
	Flaxseed oil	5.24 ± 0.74	3.31 ± 0.72	1.27 ± 0.27	1.28 ± 0.39
Khaw, 2018	Butter	6.32 ± 1.00	3.83 ± 0.90	1.99 ± 0.50	0.92 ± 0.96
	Coconut oil	6.12 ± 1.00	3.41 ± 0.90	2.28 ± 0.50	0.96 ± 0.96
	Olive oil	6.03 ± 0.90	3.64 ± 1.00	1.90 ± 0.50	0.91 ± 0.96
Kontogianni, 2013	Flaxseed oil	4.40 ± 0.70	2.51 ± 0.60	1.57 ± 0.30	0.68 ± 0.20
	Olive oil	4.51 ± 0.70	2.59 ± 0.60	1.55 ± 0.30	0.76 ± 0.40
Kris-Etherton, 1993	Butter	4.56 ± 0.34	2.93 ± 0.34	1.17 ± 0.22	0.99 ± 0.13
	Soybean oil	3.60 ± 0.34	2.15 ± 0.34	1.17 ± 0.22	0.82 ± 0.13
	Olive oil	3.94 ± 0.34	2.38 ± 0.34	1.24 ± 0.22	0.95 ± 0.13
Kruse, 2015	Rapeseed oil	4.69 ± 0.64	2.94 ± 0.53	1.09 ± 0.20	1.46 ± 0.69
	Olive oil	4.98 ± 0.78	3.18 ± 0.68	1.19 ± 0.31	1.35 ± 0.72
Lichtenstein, 1993 Schwab, 1998	Beef tallow	5.63 ± 0.79	3.62 ± 0.70	1.15 ± 0.24	1.31 ± 0.38
	Corn oil	5.02 ± 0.49	3.24 ± 0.49	1.14 ± 0.23	1.22 ± 0.35
	Rapeseed oil	5.02 ± 0.52	3.26 ± 0.44	1.14 ± 0.26	1.23 ± 0.29

	Olive oil	5.31 ± 0.49	3.42 ± 0.49	1.19 ± 0.23	1.26 ± 0.33
Lichtenstein, 1999	Butter	6.50 ± 0.93	4.58 ± 0.83	1.17 ± 0.26	1.64 ± 0.64
	Soybean oil	5.83 ± 0.83	3.99 ± 0.73	1.11 ± 0.23	1.61 ± 0.72
Lucci, 2016	Palm oil	5.02 ± 0.82	2.78 ± 0.94	1.15 ± 0.46	2.38 ± 1.54
	Olive oil	4.81 ± 0.74	2.48 ± 0.88	1.12 ± 0.43	2.77 ± 1.39
Maki, 2015	Corn oil	5.36 ± 0.76	3.52 ± 0.63	1.18 ± 0.29	1.56 ± 0.79
	Olive oil	5.72 ± 0.76	3.81 ± 0.65	1.20 ± 0.30	1.43 ± 0.72
Morillas-Ruiz, 2014	Butter	5.39 ± 0.88	3.13 ± 0.76	1.77 ± 0.35	1.07 ± 0.47
	Olive oil	5.07 ± 0.98	2.87 ± 0.90	1.75 ± 0.35	1.00 ± 0.50
Ng, 1992	Palm oil	4.98 ± 0.68	3.40 ± 0.67	1.05 ± 0.23	1.23 ± 0.80
	Olive oil	5.01 ± 0.80	3.40 ± 0.79	1.05 ± 0.22	1.20 ± 0.64
Nigam, 2014	Rapeseed oil	NR	NR	1.05 ± 0.15	1.75 ± 0.55
	Olive oil	NR	NR	1.07 ± 0.12	1.92 ± 0.66
Paschos, 2007	Flaxseed oil	6.16 ± 0.85	NR	1.00 ± 0.19	1.89 ± 0.96
	Safflower oil	5.23 ± 1.06	NR	0.90 ± 0.16	1.65 ± 0.96
Pedersen, 2000	Rapeseed oil	3.67 ± 0.81	1.73 ± 0.59	0.98 ± 0.25	0.73 ± 0.17
	Sunflower oil	3.74 ± 0.68	1.89 ± 0.47	0.90 ± 0.21	0.72 ± 0.21
	Olive oil	4.15 ± 0.76	2.16 ± 0.59	0.97 ± 0.21	0.86 ± 0.30
Perez-Jimenez, 1995	Sunflower oil	3.95 ± 0.60	2.40 ± 0.50	1.37 ± 0.23	0.82 ± 0.23
	Olive oil	4.26 ± 0.73	2.64 ± 0.69	1.41 ± 0.05	0.85 ± 0.18
Perona, 2004	Sunflower oil	4.80 ± 1.01	2.92 ± 0.82	1.45 ± 0.47	0.97 ± 0.37
	Olive oil	4.48 ± 1.07	2.66 ± 0.80	1.29 ± 0.42	1.09 ± 0.48
Rallidis, 2003	Flaxseed oil	5.85 ± 0.93	3.94 ± 0.93	1.06 ± 0.26	1.84 ± 0.98
	Safflower oil	5.65 ± 1.19	3.86 ± 1.14	0.99 ± 0.21	1.76 ± 0.97
Reiser, 1985	Beef fat	4.01 ± 0.32	2.54 ± 0.48	1.19 ± 0.12	0.99 ± 0.16
	Coconut oil	4.35 ± 0.32	2.85 ± 0.44	1.19 ± 0.12	0.88 ± 0.17
	Safflower oil	3.65 ± 0.32	2.33 ± 0.49	1.04 ± 0.12	0.81 ± 0.17
Salar, 2016	Rapeseed oil	4.07 ± 0.70	2.50 ± 1.06	1.08 ± 0.18	2.10 ± 0.46
	Sunflower oil	4.21 ± 1.02	2.23 ± 0.93	1.04 ± 0.20	2.10 ± 0.59

Scholtz, 2004	Palm oil	5.57 ± 0.89	3.59 ± 0.91	1.07 ± 0.32	1.60 ± 1.59
	Sunflower oil	4.97 ± 1.06	3.27 ± 0.99	0.97 ± 0.64	1.25 ± 1.07
Schwab, 1995	Coconut oil	4.85 ± 0.62	2.85 ± 0.46	1.49 ± 0.27	0.87 ± 0.35
	Palm oil	4.91 ± 0.74	2.93 ± 0.50	1.52 ± 0.31	0.87 ± 0.31
Schwab, 2006	Hempseed oil	5.57 ± 0.60	3.58 ± 0.68	1.53 ± 0.30	1.03 ± 0.44
	Flaxseed oil	5.60 ± 0.42	3.62 ± 0.53	1.50 ± 0.35	1.07 ± 0.37
Sirtori, 1986	Corn oil	6.12 ± 1.52	4.47 ± 1.32	1.05 ± 0.21	1.67 ± 0.73
	Olive oil	6.37 ± 1.40	4.63 ± 1.17	1.08 ± 0.23	1.74 ± 0.64
Sirtori, 1992	Corn oil	6.94 ± 0.76	5.17 ± 0.69	1.31 ± 0.24	1.29 ± 0.42
	Olive oil	7.09 ± 0.80	5.19 ± 0.76	1.34 ± 0.28	1.51 ± 0.55
Stricker, 2008	Rapeseed oil	4.42 ± 0.89	2.42 ± 0.65	1.46 ± 0.40	1.29 ± 0.96
	Sunflower oil	4.87 ± 1.46	2.71 ± 1.31	1.63 ± 0.60	1.24 ± 0.96
Sundram, 1995	Palm oil	4.54 ± 0.62	2.56 ± 0.49	1.23 ± 0.28	0.85 ± 0.31
	Rapeseed oil	4.44 ± 0.67	2.44 ± 0.50	1.23 ± 0.31	0.94 ± 0.34
Sundram, 2007	Palm oil	4.93 ± 0.58	3.08 ± 0.54	1.43 ± 0.24	0.91 ± 0.34
	Soybean oil	5.03 ± 0.69	3.30 ± 0.63	1.32 ± 0.24	0.88 ± 0.35
Teng, 2010	Palm oil	4.48 ± 0.26	2.69 ± 0.32	1.63 ± 0.19	0.83 ± 0.64
	Soybean oil	4.72 ± 0.26	3.11 ± 0.32	1.42 ± 0.19	0.89 ± 0.64
Tholstrup, 2011	Lard	4.17 ± 1.08	2.31 ± 0.74	1.22 ± 0.29	0.83 ± 0.96
	Palm oil	4.17 ± 1.08	2.33 ± 0.74	1.23 ± 0.29	0.77 ± 0.96
	Olive oil	3.93 ± 1.08	2.11 ± 0.74	1.20 ± 0.29	0.87 ± 0.96
Utarwuthipong, 2009	Palm oil	6.85 ± 0.65	4.78 ± 0.63	1.54 ± 0.35	1.17 ± 0.38
	Soybean oil	5.95 ± 0.72	4.09 ± 0.61	1.34 ± 0.32	1.16 ± 0.42
Vega-López, 2009	Corn oil	5.91 ± 0.64	3.89 ± 0.52	1.47 ± 0.36	1.42 ± 0.63
	Soybean oil	5.52 ± 0.59	3.51 ± 0.48	1.50 ± 0.37	1.37 ± 0.59
Voon, 2011	Coconut oil	4.95 ± 0.69	3.30 ± 0.75	1.37 ± 0.30	0.90 ± 0.39
	Palm oil	4.81 ± 0.74	3.20 ± 0.71	1.31 ± 0.26	0.85 ± 0.31
	Olive oil	4.65 ± 0.71	3.06 ± 0.64	1.28 ± 0.23	0.84 ± 0.37
Wardlaw, 1990	Butter	6.30 ± 0.72	4.58 ± 0.72	1.12 ± 0.22	1.65 ± 0.63

	Corn oil	4.95 ± 0.72	3.41 ± 0.58	1.09 ± 0.22	1.30 ± 0.49
	Sunflower oil	5.26 ± 0.72	3.62 ± 0.72	1.12 ± 0.22	1.49 ± 0.45
Zhang, 1997	Lard	3.99 ± 1.24	2.85 ± 1.23	0.96 ± 0.16	1.21 ± 0.47
	Palm oil	3.36 ± 0.53	2.13 ± 0.53	1.00 ± 0.20	1.13 ± 0.51
	Soybean oil	3.36 ± 0.67	2.22 ± 0.62	0.90 ± 0.22	1.23 ± 0.45

Supplemental Table S2: Post-intervention values (and standard deviation) of the included trials according to study arms investigating the effects of different oils and solid fats on blood lipids.

	SFA (g/100g)	MUFA (g/100g)	PUFA (g/100g)
Safflower oil	7	79	14
Sunflower oil	10	20	66
Rapeseed oil	7	63	28
Hempseed oil	7	13	73
Flaxseed oil	9	18	68
Corn oil	13	28	55
Olive oil	14	73	11
Soybean oil	16	23	58
Palm oil	49	37	9
Coconut oil	82	6	2
Butter	51	23	3
Lard	39	45	11
Beef fat	50	42	4

Supplemental Table S3: Fatty acid composition (g/100) of the included oils and solid fats (USDA Food composition database: https://ndb.nal.usda.gov/ndb/)

**Total cholesterol**																								
Safflower oil	0	100	0	100	0	100	49	51	0	100	0	100	0	100	0	100	58	42	46	54	0	100	0	100
Sunflower oil			33	67	0	100	36	64	26	74	33	67	16	84	50	50	0	100	0	100	0	100	12	88
Rapeseed oil					0	100	0	100	19	81	45	55	0	100	18	82	0	100	26	74	0	100	0	100
Hempseed oil							100	0	0	100	31	69	0	100	0	100	0	100	0	100	0	100	0	100
Flaxseed oil									21	79	0	100	0	100	0	100	0	100	0	100	0	100	0	100
Corn oil											44	56	31	69	0	100	0	100	23	77	0	100	28	72
Olive oil													20	80	38	62	32	68	0	100	0	100	53	47
Soybean oil															46	54	14	86	0	100	27	73	37	63
Palm oil																	35	65	36	64	53	47	0	100
Coconut oil																			0	100	0	100	16	84
Beef fat																					0	100	21	79
Lard																							29	71
Butter																								

Supplemental Table S4: Percentage contribution of each direct estimate derived from direct (blue) and indirect (red) comparisons (the colour corresponds to the percentage of contribution). The values above the oils/solid fats correspond to the percentage contribution of direct and indirect comparisons between the row and columns for total-cholesterol (e.g., the percentage contribution of direct comparisons for total cholesterol between Sunflower oil and Butter is 12% and 88% for the indirect comparisons).

LDL-cholesterol

	Sunflower oil		Rapeseed oil		Hempseed oil		Flaxseed oil		Corn oil		Olive oil		Soybean oil		Palm oil		Coconut oil		Beef fat		Lard		Butter	
Safflower oil	0	100	0	100	0	100	36	64	0	100	0	100	0	100	0	100	59	41	45	55	0	100	0	100
Sunflower oil			36	64	0	100	42	58	27	73	36	64	16	84	44	56	0	100	0	100	0	100	12	88
Rapeseed oil					0	100	0	100	20	80	39	61	0	100	21	79	0	100	26	74	0	100	0	100
Hempseed oil							100	0	0	100	0	100	0	100	0	100	0	100	0	100	0	100	0	100
Flaxseed oil									21	79	33	67	0	100	0	100	0	100	0	100	0	100	0	100
Corn oil											41	59	32	68	38	62	0	100	23	77	0	100	29	71
Olive oil													21	79	0	100	31	69	21	79	33	67	53	47
Soybean oil															46	54	14	86	0	100	24	76	37	63
Palm oil																	38	62	0	100	53	47	0	100
Coconut oil																			35	65	0	100	18	82
Beef fat																					0	100	0	100
Lard																							0	100
Butter																								

Supplemental Table S5: Percentage contribution of each direct estimate derived from direct (blue) and indirect (red) comparisons (the colour corresponds to the percentage of contribution). The values above the oils/solid fats correspond to the percentage contribution of direct and indirect comparisons between the row and columns for LDL-cholesterol (LDL-C) (e.g., the percentage contribution of direct comparisons for LDL-C between Sunflower oil and Butter is 12% and 88% for the indirect comparisons).

**HDL-cholesterol**																									
Safflower oil		0	100	0	100	0	100	66	34	0	100	0	100	0	100	0	100	51	49	56	44	0	100	0	100
		Sunflower oil		35	65	0	100	37	63	26	74	32	68	16	84	41	59	13	87	0	100	0	100	0	100
				Rapeseed oil		0	100	0	100	9	91	63	37	0	100	9	91	0	100	13	87	0	100	0	100
						Hempseed oil		100	0	0	100	0	100	0	100	0	100	0	100	0	100	0	100	0	100
								Flaxseed oil		11	89	24	76	0	100	0	100	0	100	0	100	0	100	0	100
										Corn oil		52	48	23	77	0	100	13	87	12	88	0	100	41	59
												Olive oil		12	88	42	58	20	80	11	89	23	77	57	43
														Soybean oil		46	54	42	58	0	100	36	64	36	64
																Palm oil		22	78	0	100	56	44	0	100
																		Coconut oil		51	49	0	100	5	95
																				Beef fat		0	100	0	100
																						Lard		0	100
																								Butter	

Supplemental Table S6: Percentage contribution of each direct estimate derived from direct (blue) and indirect (red) comparisons (the colour corresponds to the percentage of contribution). The values above the oils/solid fats correspond to the percentage contribution of direct and indirect comparisons between the row and columns for HDL-cholesterol (HDL-C) (e.g., the percentage contribution of direct comparisons for HDL-C between Sunflower oil and Butter is 0% and 100% for the indirect comparisons).

**Triacylglycerols**																								
Safflower oil	0	100	0	100	0	100	18	82	0	100	0	100	0	100	0	100	60	40	63	37	0	100	0	100
	Sunflower oil		47	53	0	100	15	85	11	89	57	43	0	100	43	57	0	100	0	100	0	100	5	95
			Rapeseed oil		0	100	0	100	20	80	41	59	0	100	20	80	0	100	17	83	0	100	0	100
					Hempseed oil		100	0	0	100	0	100	0	100	0	100	0	100	0	100	0	100	0	100
							Flaxseed oil		11	89	71	29	0	100	0	100	0	100	0	100	0	100	0	100
									Corn oil		46	54	9	91	0	100	0	100	17	83	0	100	25	75
											Olive oil		47	53	31	69	34	66	12	88	14	86	66	34
													Soybean oil		38	62	3	97	0	100	42	58	55	45
															Palm oil		51	49	0	100	50	50	0	100
																	Coconut oil		56	44	0	100	4	96
																			Beef fat		0	100	0	100
																					Lard		0	100
																							Butter	

Supplemental Table S7: Percentage contribution of each direct estimate derived from direct (blue) and indirect (red) comparisons (the colour corresponds to the percentage of contribution). The values above the oils/solid fats correspond to the percentage contribution of direct and indirect comparisons between the row and columns for triacylglycerols (TG) (e.g., the percentage contribution of direct comparisons for TG between Sunflower oil and Butter is 5% and 95% for the indirect comparisons).

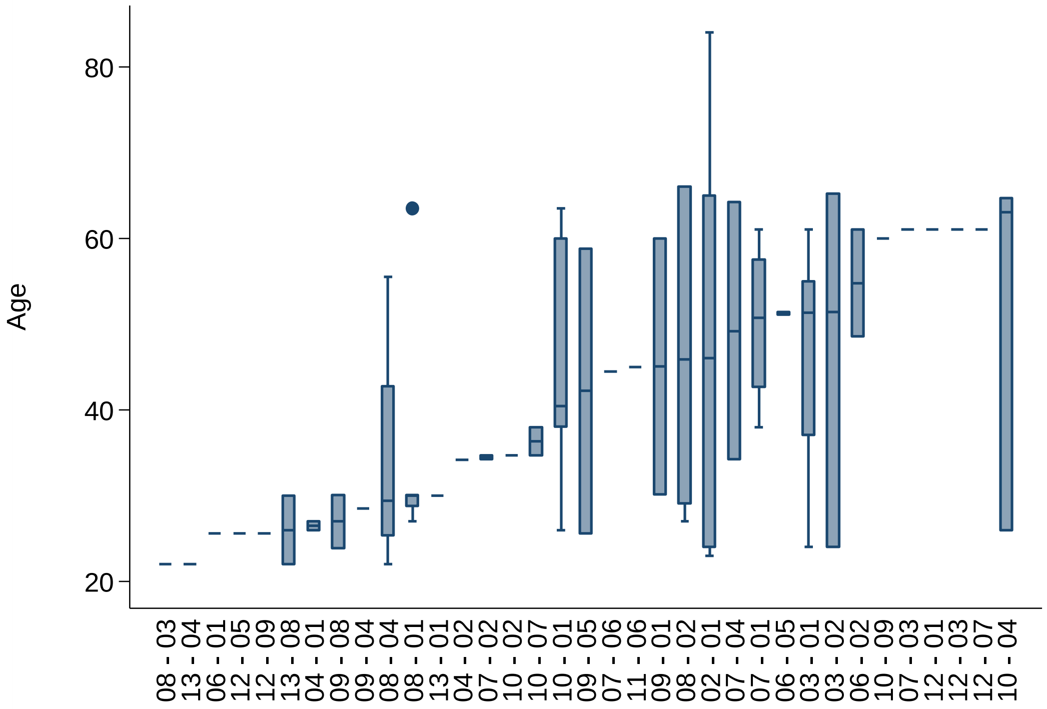

Supplemental Figure S3: Box plots showing the distribution of the mean age (years) of the trials across the available direct comparisons.

[1=Olive oil, 2=Sunflower oil, 3=Rapeseed oil, 4=Soybean oil, 5=Safflower oil, 6=Flaxseed oil, 7=Corn oil, 8=Palm oil, 9=Coconut oil, 10=Butter, 11=Hempseed oil, 12=Beef fat, 13=Lard].

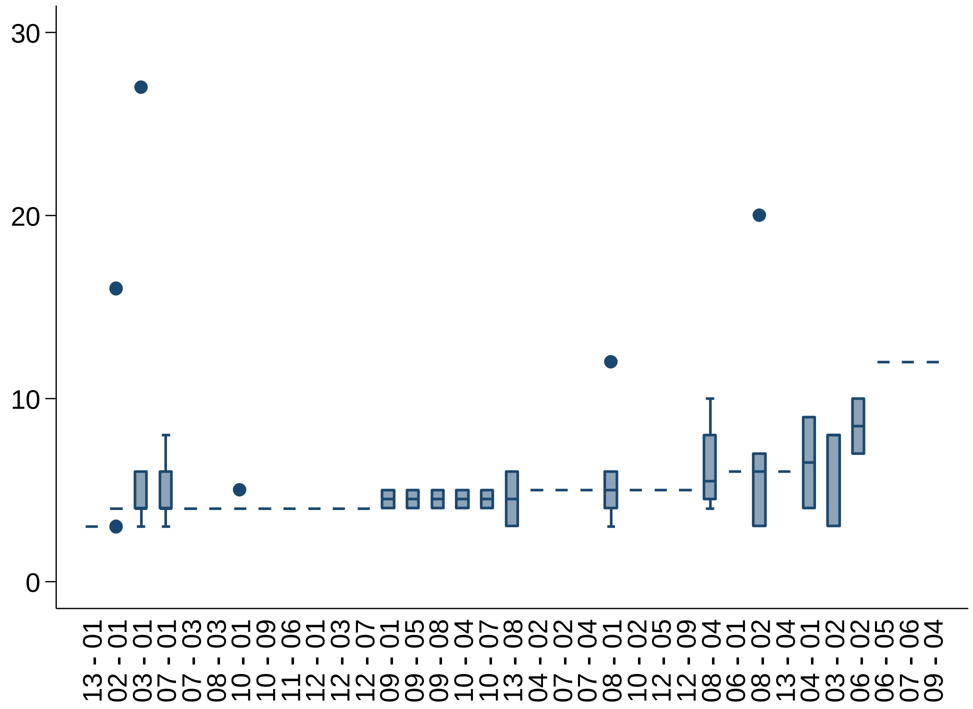

Supplemental Figure S4: Box plots showing the distribution of the study duration (weeks) across the available direct comparisons.

[1=Olive oil, 2=Sunflower oil, 3=Rapeseed oil, 4=Soybean oil, 5=Safflower oil, 6=Flaxseed oil, 7=Corn oil, 8=Palm oil, 9=Coconut oil, 10=Butter, 11=Hempseed oil, 12=Beef fat, 13=Lard].

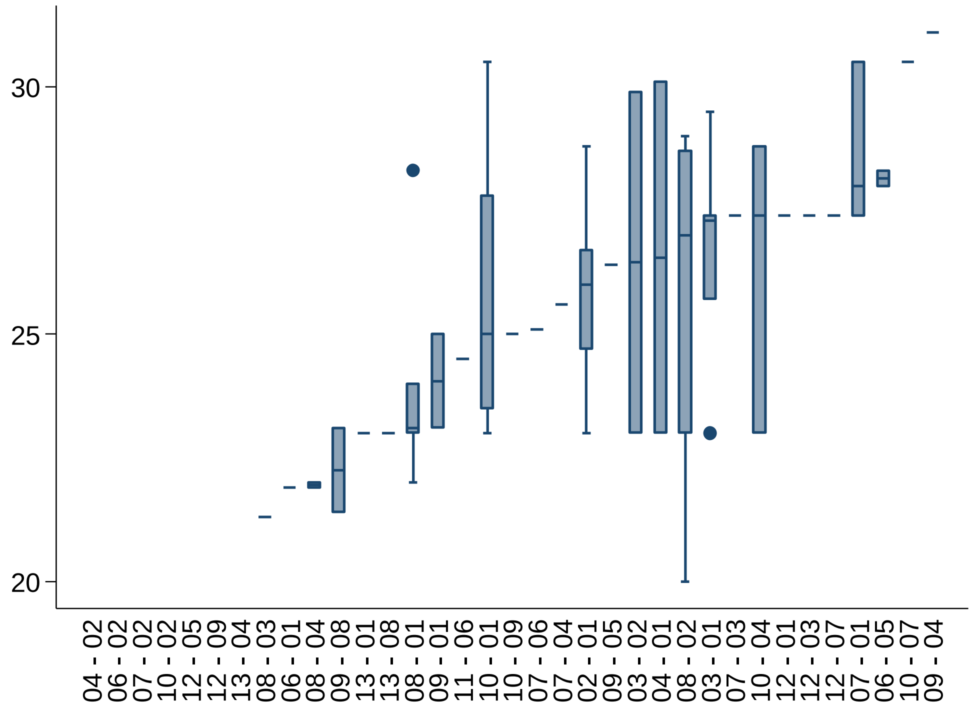

Supplemental Figure S5: Box plots showing the distribution of Body Mass Index (kg/m^2) across the available direct comparisons.

For the comparison 04/06/07/10 vs. 02, 12 vs. 05/09, and 13 vs. 04, BMI was not reported across the included trials.

[1=Olive oil, 2=Sunflower oil, 3=Rapeseed oil, 4=Soybean oil, 5=Safflower oil, 6=Flaxseed oil, 7=Corn oil, 8=Palm oil, 9=Coconut oil, 10=Butter, 11=Hempseed oil, 12=Beef fat, 13=Lard].

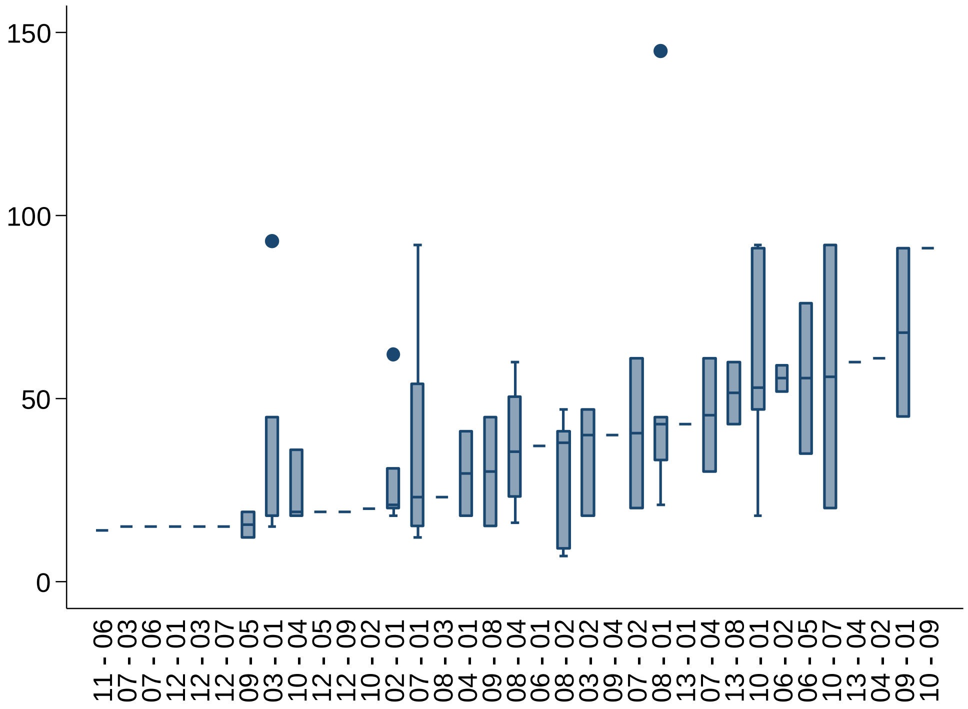

Supplemental Figure S6: Box plots showing the distribution of sample size across the available direct comparisons.

[1=Olive oil, 2=Sunflower oil, 3=Rapeseed oil, 4=Soybean oil, 5=Safflower oil, 6=Flaxseed oil, 7=Corn oil, 8=Palm oil, 9=Coconut oil, 10=Butter, 11=Hempseed oil, 12=Beef fat, 13=Lard].

**Treatment**	**SUCRA**
Olive oil	43
**Sunflower oil**	**72**
**Rapeseed**	**85**
Soybean oil	59
**Safflower oil**	**90**
Flaxseed oil	59
Hempseed oil	61
Corn oil	72
Palm oil	34
Coconut oil	22
Beef fat	41
Lard	11
Butter	3

Supplemental Table S8: Rankogram for total cholesterol

**Treatment**	**SUCRA**
Olive oil	37
**Sunflower oil**	**71**
**Rapeseed**	**76**
Soybean oil	50
**Safflower oil**	**82**
**Flaxseed oil**	**71**
Hempseed oil	69
Corn oil	66
Palm oil	33
Coconut oil	33
Beef fat	50
Lard	10
Butter	2

Supplemental Table S9: Rankogram for LDL-cholesterol

**Treatment**	**SUCRA**
Olive oil	52
Sunflower oil	57
Rapeseed	53
Soybean oil	13
Safflower oil	6
Flaxseed oil	47
Hempseed oil	59
Corn oil	29
**Palm oil**	**80**
**Coconut oil**	**88**
**Beef fat**	**74**
Lard	55
Butter	37

Supplemental Table S10: Rankogram for HDL-cholesterol

**Treatment**	**SUCRA**
Olive oil	32
Sunflower oil	61
Rapeseed	58
**Soybean oil**	**72**
**Safflower oil**	**68**
Flaxseed oil	56
Hempseed oil	63
Corn oil	66
**Palm oil**	**74**
Coconut oil	29
Beef fat	6
Lard	50
Butter	17

Supplemental Table S11: Rankogram for triacyglycerols

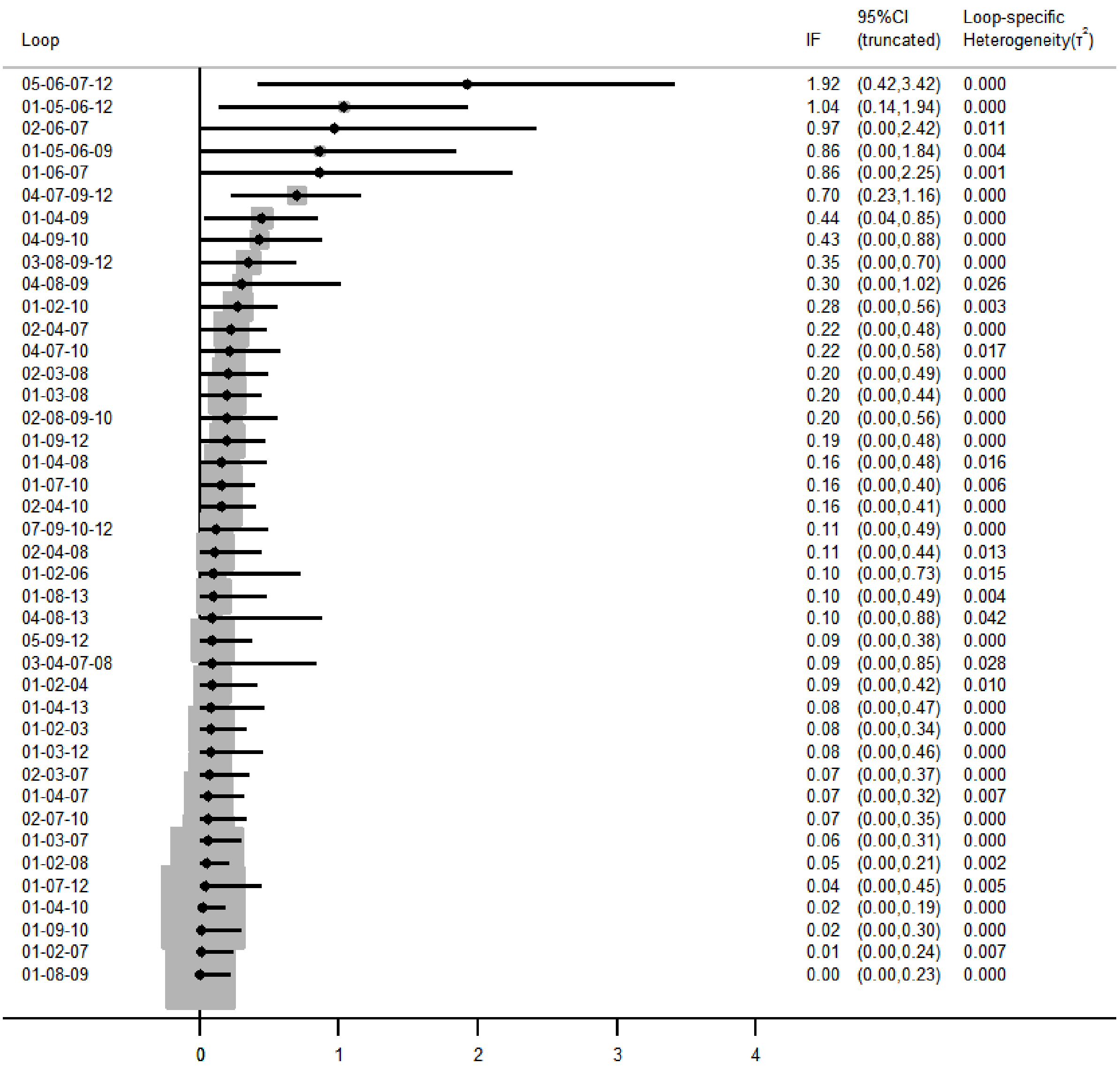

Supplemental Figure S7: Loop-specific approach for inconsistency for total cholesterol

[1=Olive oil, 2=Sunflower oil, 3=Rapeseed oil, 4=Soybean oil, 5=Safflower oil, 6=Flaxseed oil, 7=Corn oil, 8=Palm oil, 9=Coconut oil, 10=Butter, 11=Hempseed oil, 12=Beef fat, 13=Lard].

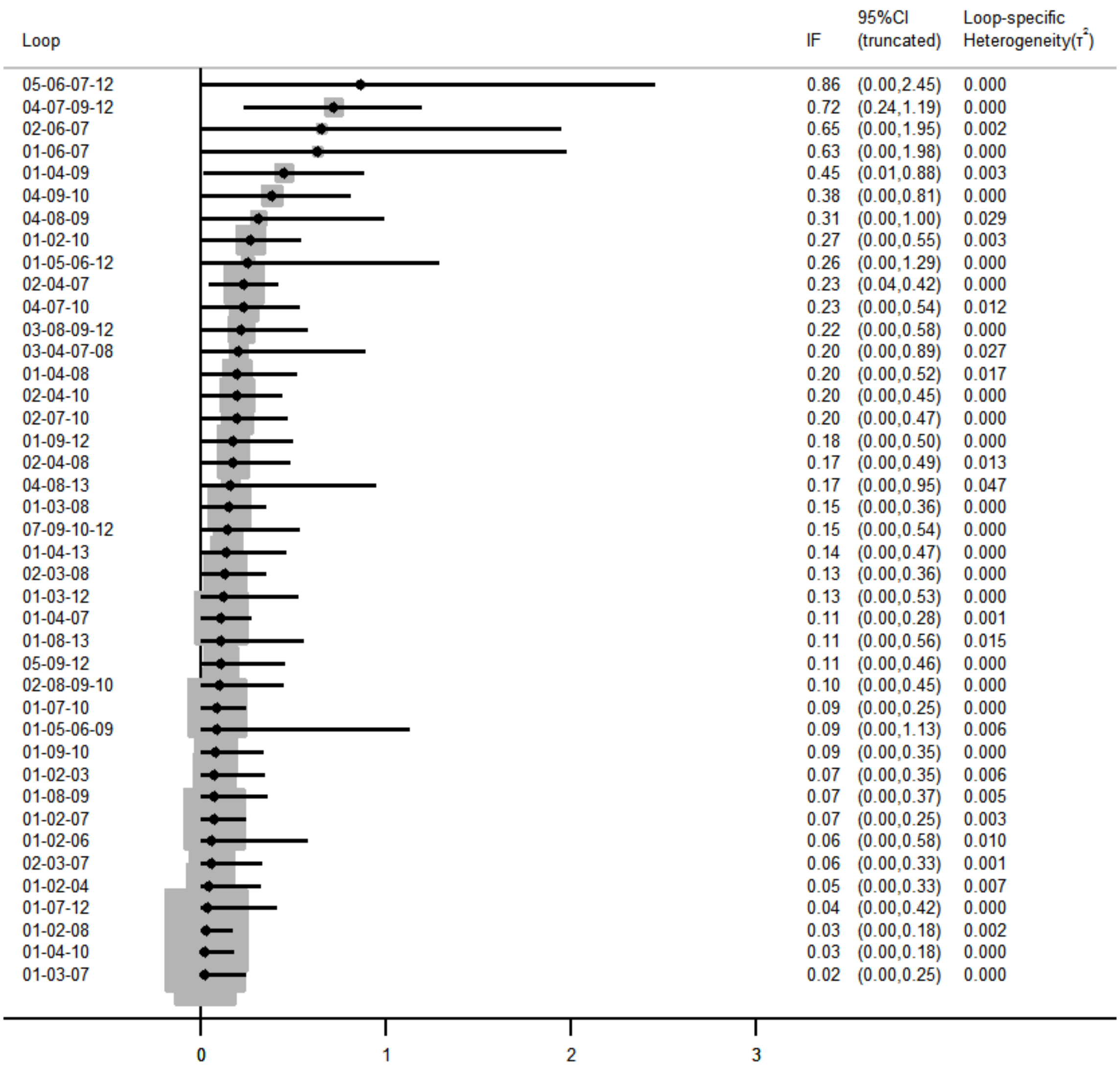

Supplemental Figure S8: Loop-specific approach for inconsistency for LDL-cholesterol

[1=Olive oil, 2=Sunflower oil, 3=Rapeseed oil, 4=Soybean oil, 5=Safflower oil, 6=Flaxseed oil, 7=Corn oil, 8=Palm oil, 9=Coconut oil, 10=Butter, 11=Hempseed oil, 12=Beef fat, 13=Lard].

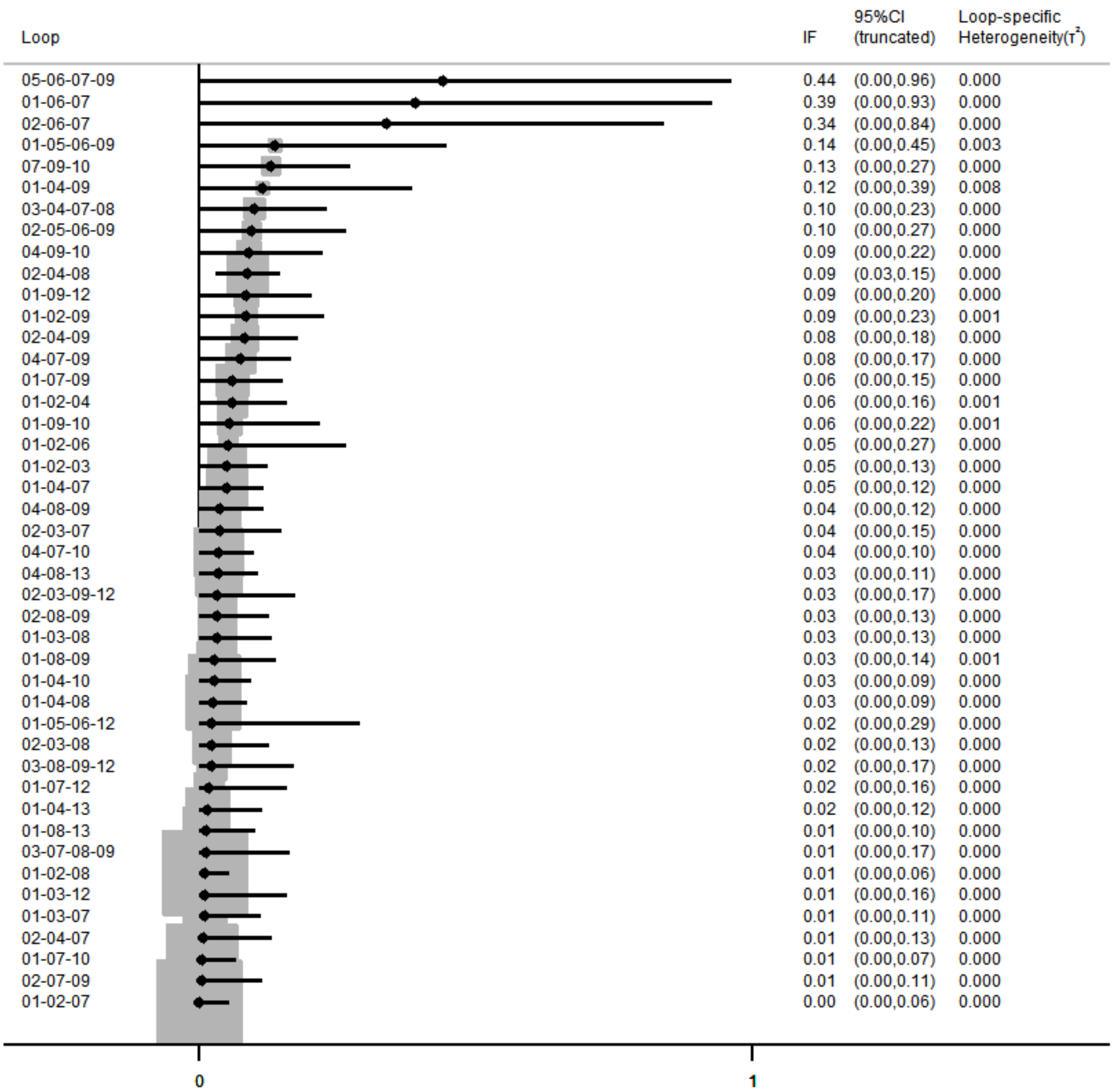

Supplemental Figure S9: Loop-specific approach for inconsistency for HDL-cholesterol

[1=Olive oil, 2=Sunflower oil, 3=Rapeseed oil, 4=Soybean oil, 5=Safflower oil, 6=Flaxseed oil, 7=Corn oil, 8=Palm oil, 9=Coconut oil, 10=Butter, 11=Hempseed oil, 12=Beef fat, 13=Lard].

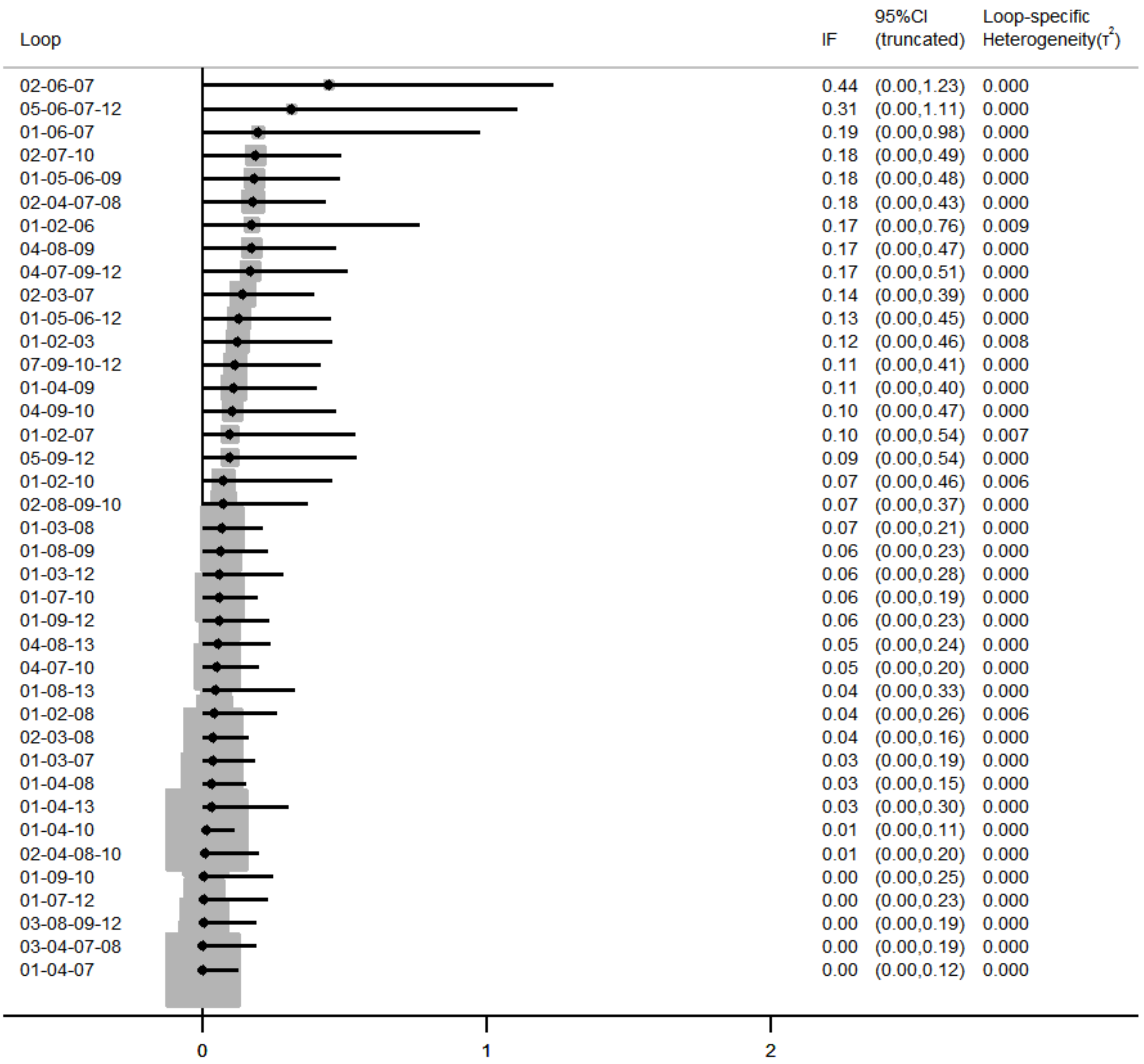

Supplemental Figure S10: Loop-specific approach for inconsistency for triacylglycerols

[1=Olive oil, 2=Sunflower oil, 3=Rapeseed oil, 4=Soybean oil, 5=Safflower oil, 6=Flaxseed oil, 7=Corn oil, 8=Palm oil, 9=Coconut oil, 10=Butter, 11=Hempseed oil, 12=Beef fat, 13=Lard].

Side	Direct		Indirect		Difference		
	Coefficient	SE	Coefficient	SE	Coefficient	SE	p-value
01 02	-.0563865	.0672811	-.1236772	.0567887	.0672908	.0886215	0.448
01 03	-.1876418	.0851309	-.1040587	.1029725	-.0835831	.133116	0.530
01 04	-.1125267	.0971157	-.0307684	.0559252	-.0817584	.1120632	0.466
01 06	-.1612903	.2577541	-.0232793	.134419	-.138011	.2906985	0.635
01 07	-.1063635	.0634316	-.0765319	.0756783	-.0298316	.0989477	0.763
01 08	.0791713	.0667012	-.0033021	.0563206	.0824733	.0871175	0.344
01 09	.1152456	.1013413	.0721782	.1112525	.0430674	.1497116	0.774
01 10	.2011443	.0635913	.4134186	.0863143	-.2122743	.1084647	0.050
01 12	.1277558	.1535207	-.0354029	.1161521	.1631588	.184312	0.376
01 13	.1411765	.1685323	.2470356	.1445261	-.1058591	.2220156	0.633
02 03	-.1001755	.1245581	-.0394507	.0833675	-.0607248	.149353	0.684
02 04	.0373916	.1132261	.0461808	.0595343	-.0087891	.127928	0.945
02 06	.0158475	.1395467	.1034349	.2089282	-.0875874	.2512922	0.727
02 07	-.0499041	.0899698	.0332675	.0704872	-.0831717	.1143852	0.467
02 08	.1694329	.0528114	.061279	.06478	.1081538	.083279	0.194
02 10	.5199999	.148662	.3441958	.0664887	.1758041	.1628531	0.280
03 07	-.0013996	.1349999	.0867606	.0897352	-.0881602	.1619277	0.586
03 08	.05	.1362743	.2316134	.0807702	-.1816134	.1584125	0.252
03 12	.2719346	.1546266	.0916595	.1446164	.1802751	.2070054	0.384
04 07	.0713761	.0816338	-.1296666	.0700838	.2010427	.1071697	0.061
04 08	.0076626	.0580037	.1661853	.0636695	-.1585226	.0861619	0.066
04 09	-.2008534	.2159649	.2014144	.0844112	-.4022679	.2318752	0.083
04 10	.3352057	.0781568	.3225092	.0849174	.0126964	.1152233	0.912
04 13	.2897824	.165464	.2292949	.1375837	.0604875	.2037011	0.767
05 06	.8907421	.3762066	.0238135	.1635103	.8669286	.4102533	**0.035**
05 09	.3019908	.0921102	.3959759	.2932359	-.0939851	.3079377	0.760
05 12	.1702155	.1116331	.4750184	.2134059	-.3048029	.2408491	0.206
06 07	.9023685	.6653865	-.0754185	.1262889	.977787	.6772654	0.149
06 11	-.0244379	.1865398	.080973	200.7875	-.105411	200.7875	1.000
07 10	.479397	.0980302	.3039855	.077803	.1754115	.1254055	0.162
07 12	.2825151	.1520277	.0053328	.1260141	.2771823	.1904169	0.145
08 09	.0625479	.1221386	.065078	.103476	-.0025301	.1600673	0.987
08 13	.177706	.116884	.1441221	.2063672	.0335839	.2218365	0.880
09 10	.0687349	.1551582	.2265326	.0994255	-.1577977	.1821501	0.386
09 12	-.1624837	.1092771	.0781292	.1451464	-.2406128	.1816867	0.185

Supplemental Table S12: Side-splitting approach for inconsistency for total cholesterol

[1=Olive oil, 2=Sunflower oil, 3=Rapeseed oil, 4=Soybean oil, 5=Safflower oil, 6=Flaxseed oil, 7=Corn oil, 8=Palm oil, 9=Coconut oil, 10=Butter, 11=Hempseed oil, 12=Beef fat, 13=Lard].

Side	Direct		Indirect		Difference		
	Coefficient	SE	Coefficient	SE	Coefficient	SE	p-value
01 02	-.0481929	.0628082	-.1231825	.0572818	.0749895	.0851395	0.378
01 03	-.1457872	.0872241	-.0642647	.0984155	-.0815224	.1319029	0.537
01 04	-.083408	.0961299	-.0140464	.0549649	-.0693616	.1107005	0.531
01 06	-.1173021	.2279386	-.1098363	.126318	-.0074658	.2605998	0.977
01 07	-.1090954	.064981	-.0322926	.0727777	-.0768028	.0977631	0.432
01 08	.0836541	.0641472	-.0393086	.0551392	.1229627	.08447	0.145
01 09	.0365344	.1024961	.0022216	.1123611	.0343128	.1512998	0.821
01 10	.1757125	.0636905	.3903575	.084758	-.214645	.1072658	**0.045**
01 12	.0701107	.1632621	-.0804823	.1245505	.150593	.1924817	0.434
01 13	.1176471	.1382308	.225439	.1402024	-.1077919	.1968869	0.584
02 03	-.0421385	.1130288	-.008892	.0838783	-.0332465	.1401769	0.813
02 04	.0124467	.1109779	.0709988	.0588467	-.0585521	.1256147	0.641
02 06	-.0052882	.1251784	-.066448	.2010634	.0611598	.2368239	0.796
02 07	-.0303841	.0879405	.0426593	.0703248	-.0730434	.1126497	0.517
02 08	.1464184	.0569234	.0480644	.0631721	.0983541	.0845791	0.245
02 10	.4799999	.1506276	.3179188	.0653678	.1620811	.1641999	0.324
03 07	-.011794	.1323367	.0558729	.0888516	-.0676669	.1596949	0.672
03 08	.06	.1250527	.1503634	.0808386	-.0903634	.1489062	0.544
03 12	.1355351	.1604456	.0341597	.1525386	.1013754	.2119765	0.632
04 07	.0979292	.0750564	-.158285	.0672234	.2562142	.1005235	**0.011**
04 08	-.0561245	.055297	.1547312	.0601292	-.2108558	.0816272	**0.010**
04 09	-.3156269	.2110113	.1136061	.0853579	-.4292331	.2276219	0.059
04 10	.28716	.076894	.2844217	.0821654	.0027383	.1124231	0.981
04 13	.2747224	.1639334	.1654166	.1196946	.1093057	.1941103	0.573
05 06	.1379792	.4715461	.0420678	.1615175	.0959114	.4984412	0.847
05 09	.2096092	.1015203	-.067987	.3211828	.2775962	.3359721	0.409
05 12	.0985416	.1289159	.2558489	.2318728	-.1573073	.2653179	0.553
06 07	.630421	.659177	.0173121	.1173829	.6131089	.6695471	0.360
06 11	-.0325839	.2125228	.1898089	200.7061	-.2223928	200.7061	0.999
07 10	.4250857	.0939569	.2643224	.0773463	.1607632	.1216071	0.186
07 12	.166618	.1626585	-.0310908	.1346139	.1977088	.199765	0.322
08 09	.0305918	.1229572	-.0095603	.1032704	.0401521	.1599251	0.802
08 13	.1562227	.1054104	.1606229	.1899242	-.0044002	.2062741	0.983
09 10	.1961302	.147887	.2504876	.1000794	-.0543574	.1763012	0.758
09 12	-.1469525	.1246866	.1029429	.1562018	-.2498953	.1994758	0.210

Supplemental Table S13: Side-splitting approach for inconsistency for LDL-cholesterol

[1=Olive oil, 2=Sunflower oil, 3=Rapeseed oil, 4=Soybean oil, 5=Safflower oil, 6=Flaxseed oil, 7=Corn oil, 8=Palm oil, 9=Coconut oil, 10=Butter, 11=Hempseed oil, 12=Beef fat, 13=Lard].

Side	Direct		Indirect		Difference		
	Coefficient	SE	Coefficient	SE	Coefficient	SE	p-value
01 02	-8.28e-06	.0145317	.0064745	.01385	-.0064828	.0200871	0.747
01 03	-.0186144	.0223162	.032027	.0275837	-.0506414	.0350319	0.148
01 04	-.0366987	.0251828	-.031688	.0128872	-.0050106	.0283827	0.860
01 06	.0293255	.1026383	-.0077468	.0289962	.0370723	.1066555	0.728
01 07	-.0097487	.0150552	-.0260088	.0184171	.0162601	.0238569	0.496
01 08	.015667	.0155972	.0234374	.0136192	-.0077704	.0207213	0.708
01 09	.0650457	.0278675	.0155864	.0192597	.0494592	.0335116	0.140
01 10	-.0051372	.0156921	-.0224879	.0241878	.0173507	.0293917	0.555
01 12	-.0188949	.0499677	.0291996	.0249944	-.0480944	.0545011	0.378
01 13	.0117647	.0379354	.000205	.0239105	.0115597	.044842	0.797
02 03	.0289435	.0294024	-.0204814	.0228322	.049425	.0369502	0.181
02 04	.0178572	.0237648	-.0519529	.0129015	.0698101	.0270538	**0.010**
02 06	-.022606	.0296338	.0506454	.0602634	-.0732513	.0671339	0.275
02 07	-.0134637	.0206188	-.0238416	.0171534	.0103779	.0268837	0.699
02 08	.0046596	.0123557	.0341364	.0148085	-.0294768	.0193438	0.128
02 09	1.13e-09	.0364538	.0353828	.018527	-.0353828	.0408916	0.387
03 07	-.0003923	.045756	-.0214964	.0220251	.0211041	.0505877	0.677
03 08	1.47e-09	.0444634	.0227718	.0205236	-.0227718	.0489715	0.642
03 12	.01125	.0525365	.0217306	.031988	-.0104806	.0610887	0.864
04 07	-.0230177	.0203536	.0397372	.0157644	-.0627549	.0257713	**0.015**
04 08	.0766272	.0136221	.0234168	.0153222	.0532105	.0204815	**0.009**
04 09	.0663535	.0262506	.0629925	.0203593	.0033609	.0332204	0.919
04 10	.0186999	.017455	.0274426	.0207341	-.0087427	.0271621	0.748
04 13	.0311619	.0278653	.0423517	.0306906	-.0111898	.0427721	0.794
05 06	.1483965	.072515	.023114	.0381864	.1252825	.081955	0.126
05 09	.0754335	.0210324	.1957216	.0645504	-.1202881	.0676949	0.076
05 12	.0746243	.0220098	.0890976	.072917	-.0144733	.076091	0.849
06 07	.3522983	.2537984	-.0161515	.0293143	.3684498	.2554858	0.149
06 11	.0244379	.100727	.0354911	198.2115	-.0110531	198.2115	1.000
07 09	.0150001	.0363813	.0573626	.0196959	-.0423626	.0413706	0.306
07 10	.0091053	.022437	.0031237	.0198752	.0059816	.0301917	0.843
07 12	.0095921	.0499796	.0436712	.0265772	-.0340791	.0554328	0.539
08 09	.0279914	.0306289	.0050417	.019143	.0229498	.0358284	0.522
08 13	-.0163707	.0217976	-.0173664	.045384	.0009957	.0514195	0.985
09 10	-.1431779	.0593081	-.0315291	.0192616	-.1116488	.0621736	0.073
09 12	-1.24e-09	.0209481	-.0620588	.045013	.0620588	.0496487	0.211

Supplemental Table S14: Side-splitting approach for inconsistency for HDL-cholesterol

[1=Olive oil, 2=Sunflower oil, 3=Rapeseed oil, 4=Soybean oil, 5=Safflower oil, 6=Flaxseed oil, 7=Corn oil, 8=Palm oil, 9=Coconut oil, 10=Butter, 11=Hempseed oil, 12=Beef fat, 13=Lard].

Side	Direct		Indirect		Difference		
	Coefficient	SE	Coefficient	SE	Coefficient	SE	p-value
01 02	-.0221634	.017449	-.044034	.0340381	.0218706	.0384473	0.569
01 03	-.0511356	.0334821	.0098058	.0386715	-.0609413	.0534127	0.254
01 04	-.0397589	.0138831	-.0247204	.0307582	-.0150386	.0337463	0.656
01 06	-.1173021	.1078027	-.0043386	.048676	-.1129634	.1182826	0.340
01 07	-.0226202	.0319109	-.0502051	.0403935	.0275849	.0515087	0.592
01 08	-.0237222	.0487899	-.0437461	.0229595	.0200239	.0539859	0.711
01 09	.0110174	.027375	-.0104487	.0463844	.0214661	.0630847	0.734
01 10	.0144807	.01334	.0539577	.0454486	-.039477	.0476457	0.407
01 12	.0362053	.0733073	.0490172	.0311386	-.0128119	.0774372	0.869
01 13	-.0014663	.1095331	-.0238077	.0578993	.0223413	.1284393	0.862
02 03	.0014174	.0350962	.0027634	.0337787	-.001346	.0486922	0.978
02 06	.0153936	.0534448	-.0198124	.0739352	.035206	.0912291	0.700
02 07	-.1344691	.1097378	.0027331	.0295369	-.1372022	.1135562	0.227
02 08	.0035383	.0311083	-.027383	.0284113	.0309212	.0421299	0.463
02 10	.1187975	.0831782	.0405122	.0197951	.0782853	.0853526	0.359
03 07	-.0083616	.0587183	-.0086478	.0390245	.0002861	.0704339	0.997
03 08	-.0391304	.0417127	-.0004469	.033178	-.0386835	.0532985	0.468
03 12	.0453925	.0699241	.0803482	.0397551	-.0349557	.0796763	0.661
04 07	.0166667	.0525287	-.0002045	.0302131	.0168711	.0605978	0.781
04 08	-.0164985	.0343965	.0059739	.0277486	-.0224725	.0442083	0.611
04 09	.1779749	.1439294	.0384478	.0207156	.1395271	.1454125	0.337
04 10	.0546314	.0138841	.0585721	.0419716	-.0039407	.0442084	0.929
04 13	-.0124458	.0631748	.0610081	.0730208	-.0734539	.0941292	0.435
05 06	.066878	.0960515	-.0071099	.0587104	.0739879	.1125735	0.511
05 09	.0326141	.0295298	.113838	.0895671	-.0812239	.0942689	0.389
05 12	.0847621	.0272043	.0660282	.1137865	.0187339	.1166412	0.872
06 07	.2855364	.3847966	-.0148867	.0507258	.300423	.3881256	0.439
06 11	-.02	.0768231	.0283261	198.9513	-.0483261	198.9513	1.000
07 10	.0979395	.0513084	.0327349	.0315575	.0652046	.0610714	0.286
07 12	.0488407	.0756605	.0903915	.0423991	-.0415508	.0864166	0.631
08 09	.037767	.0249195	.0620167	.0502423	-.0242497	.0634885	0.702
08 13	.047685	.0575486	-.0611503	.1071656	.1088353	.1252658	0.385
09 10	-.0293997	.0672345	.0178652	.02189	-.0472648	.0699697	0.499
09 12	.0495974	.0270124	.0041669	.0675893	.0454305	.0729871	0.534

Supplemental Table S15: Side-splitting approach for inconsistency for triacgylcerols

[1=Olive oil, 2=Sunflower oil, 3=Rapeseed oil, 4=Soybean oil, 5=Safflower oil, 6=Flaxseed oil, 7=Corn oil, 8=Palm oil, 9=Coconut oil, 10=Butter, 11=Hempseed oil, 12=Beef fat, 13=Lard].

Supplemental Table S16: Low risk of bias sensitivity analysis for total-cholesterol

Safflower oil												
-0.35<br>(-1.33, 0.64)	Sunflower											
-0.32<br>(-1.31, 0.67)	0.02<br>(-0.11, 0.16)	Rapeseed oil										
-0.34<br>(-1.35, 0.67)	0.00<br>(-0.43, 0.44)	-0.02<br>(-0.48, 0.43)	Hempseed oil									
-0.37<br>(-1.32, 0.58)	-0.02<br>(-0.28, 0.23)	-0.05<br>(-0.33, 0.24)	-0.02<br>(-0.38, 0.33)	Flaxseed oil								
-0.36<br>(-1.35, 0.63)	-0.01<br>(-0.12, 0.10)	-0.04<br>(-0.18, 0.10)	-0.01<br>(-0.46, 0.43)	0.01<br>(-0.27, 0.29)	Corn oil							
-0.49<br>(-1.48, 0.50)	-0.14<br>(-0.24, -0.05)	-0.17<br>(-0.29, -0.04)	-0.15<br>(-0.59, 0.30)	-0.12<br>(-0.40, 0.15)	-0.13<br>(-0.23, -0.03)	Olive oil						
-0.41<br>(-1.40, 0.58)	-0.06<br>(-0.17, 0.05)	-0.09<br>(-0.23, 0.06)	-0.06<br>(-0.51, 0.38)	-0.04<br>(-0.32, 0.24)	-0.05<br>(-0.16, 0.06)	0.08<br>(-0.02, 0.19)	Soybean oil					
-0.46<br>(-1.45, 0.53)	-0.12<br>(-0.21, -0.02)	-0.14<br>(-0.28, 0.00)	-0.12<br>(-0.56, 0.33)	-0.09<br>(-0.37, 0.18)	-0.10<br>(-0.23, 0.02)	0.03<br>(-0.08, 0.14)	-0.05<br>(-0.17, 0.07)	Palm oil				
-0.21<br>(-1.28, 0.86)	0.14<br>(-0.29, 0.57)	0.11<br>(-0.32, 0.55)	0.14<br>(-0.47, 0.75)	0.16<br>(-0.34, 0.66)	0.15<br>(-0.28, 0.58)	0.28<br>(-0.14, 0.71)	0.20<br>(-0.21, 0.61)	0.25<br>(-0.18, 0.68)	Coconut oil			
-0.65<br>(-1.66, 0.37)	-0.30<br>(-0.57, -0.03)	-0.32<br>(-0.59, -0.06)	-0.30<br>(-0.81, 0.21)	-0.28<br>(-0.65, 0.09)	-0.29<br>(-0.55, -0.03)	-0.16<br>(-0.41, 0.10)	-0.24<br>(-0.51, 0.03)	-0.18<br>(-0.46, 0.09)	-0.44<br>(-0.93, 0.06)	Beef fat		
-0.55<br>(-1.57, 0.48)	-0.20<br>(-0.48, 0.08)	-0.22<br>(-0.52, 0.07)	-0.20<br>(-0.72, 0.32)	-0.18<br>(-0.56, 0.20)	-0.19<br>(-0.48, 0.10)	-0.06<br>(-0.33, 0.22)	-0.14<br>(-0.43, 0.15)	-0.08<br>(-0.36, 0.19)	-0.34<br>(-0.84, 0.17)	0.10<br>(-0.28, 0.47)	Lard	
-0.76<br>(-1.75, 0.23)	-0.41<br>(-0.54, -0.29)	-0.44<br>(-0.59, -0.28)	-0.41<br>(-0.87, 0.04)	-0.39<br>(-0.67, -0.10)	-0.40<br>(-0.52, -0.28)	-0.27<br>(-0.38, -0.15)	-0.35<br>(-0.46, -0.24)	-0.30<br>(-0.43, -0.16)	-0.55<br>(-0.98, -0.12)	-0.11<br>(-0.38, 0.16)	-0.21<br>(-0.50, 0.08)	Butter

The value below the oils/ solid fats corresponds to the difference in mean in total cholesterol (mmol/l) between the column and the row (eg, the mean difference in average total cholesterol between safflower oil and butter is -0.76 mmol/l).

Supplemental Table S17: Low risk of bias sensitivity analysis for LDL-cholesterol

Safflower oil												
-0.14<br>(-1.10, 0.81)	Sunflower oil											
-0.14<br>(-1.11, 0.82)	-0.00<br>(-0.13, 0.13)	Rapeseed oil										
-0.11<br>(-1.12, 0.91)	0.04<br>(-0.44, 0.52)	0.04<br>(-0.46, 0.54)	Hempseed oil									
-0.14<br>(-1.06, 0.79)	0.00<br>(-0.24, 0.25)	0.00<br>(-0.27, 0.28)	-0.03<br>(-0.45, 0.38)	Flaxseed oil								
-0.17<br>(-1.13, 0.79)	-0.03<br>(-0.14, 0.09)	-0.03<br>(-0.17, 0.12)	-0.06<br>(-0.56, 0.43)	-0.03<br>(-0.30, 0.24)	Corn oil							
-0.26<br>(-1.22, 0.70)	-0.12<br>(-0.22, -0.02)	-0.12<br>(-0.25, 0.02)	-0.15<br>(-0.64, 0.34)	-0.12<br>(-0.38, 0.14)	-0.09<br>(-0.19, 0.02)	Olive oil						
-0.21<br>(-1.17, 0.75)	-0.07<br>(-0.18, 0.05)	-0.06<br>(-0.22, 0.09)	-0.10<br>(-0.60, 0.39)	-0.07<br>(-0.34, 0.20)	-0.04<br>(-0.15, 0.08)	0.05<br>(-0.06, 0.16)	Soyben oil					
-0.25<br>(-1.21, 0.71)	-0.11<br>(-0.21, -0.01)	-0.11<br>(-0.25, 0.03)	-0.15<br>(-0.64, 0.35)	-0.11<br>(-0.38, 0.15)	-0.08<br>(-0.21, 0.05)	0.01<br>(-0.11, 0.12)	-0.04<br>(-0.17, 0.08)	Palm oil				
0.11<br>(-0.94, 1.16)	0.25<br>(-0.18, 0.68)	0.25<br>(-0.19, 0.69)	0.21<br>(-0.43, 0.86)	0.25<br>(-0.25, 0.74)	0.28<br>(-0.15, 0.71)	0.37<br>(-0.06, 0.80)	0.32<br>(-0.10, 0.73)	0.36<br>(-0.07, 0.79)	Coconut oil			
-0.35<br>(-1.35, 0.65)	-0.20<br>(-0.50, 0.10)	-0.20<br>(-0.50, 0.09)	-0.24<br>(-0.81, 0.33)	-0.21<br>(-0.59, 0.18)	-0.18<br>(-0.47, 0.12)	-0.09<br>(-0.38, 0.20)	-0.14<br>(-0.44, 0.17)	-0.10<br>(-0.40, 0.21)	-0.45<br>(-0.97, 0.06)	Beef fat		
-0.31<br>(-1.29, 0.68)	-0.17<br>(-0.41, 0.08)	-0.16<br>(-0.43, 0.10)	-0.20<br>(-0.74, 0.34)	-0.17<br>(-0.51, 0.18)	-0.14<br>(-0.39,0.12)	-0.05<br>(-0.29, 0.19)	-0.10<br>(-0.35, 0.15)	-0.06<br>(-0.29, 0.18)	-0.42<br>(-0.90, 0.07)	0.04<br>(-0.33, 0.41)	Lard	
-0.51<br>(-1.48, 0.45)	-0.37<br>(-0.50, -0.24)	-0.37<br>(-0.53, -0.21)	-0.41<br>(-0.91, 0.09)	-0.38<br>(-0.65, -0.10)	-0.34<br>(-0.47, -0.22)	-0.25<br>(-0.38, -0.13)	-0.31<br>(-0.42, -0.19)	-0.26<br>(-0.41, -0.12)	-0.62<br>(-1.05, -0.19)	-0.17<br>(-0.48, 0.14)	-0.21<br>(-0.47, 0.05)	Butter

The value below the oils/ solid fats corresponds to the difference in mean in LDL-cholesterol (mmol/l) between the column and the row (eg, the mean difference in average LDL-cholesterol between safflower oil and butter is -0.51 mmol/l).

Supplemental Table S18: Low risk of bias sensitivity analysis for HDL-cholesterol												
Safflower oil												
-0.15<br>(-0.35, 0.05)	Sunflower oil											
-0.16<br>(-0.36, 0.05)	-0.00<br>(-0.04, 0.03)	Rapeseed oil										
-0.15<br>(-0.43, 0.12)	-0.00<br>(-0.21, 0.20)	0.00<br>(-0.21, 0.21)	Hempseed oil									
-0.13<br>(-0.32, 0.06)	0.02<br>(-0.04, 0.08)	0.03<br>(-0.04, 0.10)	0.02<br>(-0.17, 0.22)	Flaxseed oil								
-0.14<br>(-0.34, 0.07)	0.01<br>(-0.02, 0.04)	0.02<br>(-0.02, 0.06)	0.01<br>(-0.19, 0.22)	-0.01<br>(-0.08, 0.06)	Corn oil							
-0.16<br>(-0.36, 0.04)	-0.01<br>(-0.03, 0.02)	-0.00<br>(-0.04, 0.03)	-0.01<br>(-0.21, 0.20)	-0.03<br>(-0.10, 0.03)	-0.02<br>(-0.05, 0.00)	Olive oil						
-0.13<br>(-0.33, 0.08)	0.03<br>(-0.00, 0.05)	0.03<br>(-0.01, 0.07)	0.03<br>(-0.18, 0.24)	0.00<br>(-0.06, 0.07)	0.01<br>(-0.01, 0.04)	0.04<br>(0.01, 0.06)	Soybean oil					
-0.18<br>(-0.38, 0.02)	-0.03<br>(-0.05, -0.00)	-0.02<br>(-0.06, 0.02)	-0.03<br>(-0.23, 0.18)	-0.05<br>(-0.11, 0.01)	-0.04<br>(-0.07, -0.01)	-0.02<br>(-0.05, 0.01)	-0.05<br>(-0.08 ,-0.02)	Palm oil				
-0.18<br>(-0.38, 0.03)	-0.02<br>(-0.07, 0.02)	-0.02<br>(-0.07, 0.04)	-0.02<br>(-0.23, 0.19)	-0.05<br>(-0.12, 0.03)	-0.04<br>(-0.08, 0.01)	-0.02<br>(-0.06, 0.03)	-0.05<br>(-0.09, -0.01)	0.00<br>(-0.04, 0.05)	Coconut oil			
-0.15<br>(-0.37, 0.07)	0.00<br>(-0.09, 0.09)	0.01<br>(-0.08, 0.10)	0.01<br>(-0.22, 0.23)	-0.02<br>(-0.13, 0.09)	-0.01<br>(-0.10, 0.08)	0.01<br>(-0.08, 0.10)	-0.02<br>(-0.11, 0.07)	0.03<br>(-0.06, 0.12)	0.03<br>(-0.07 ,0.13)	Beef fat		
-0.17<br>(-0.39, 0.04)	-0.02<br>(-0.09, 0.05)	-0.02<br>(-0.09, 0.06)	-0.02<br>(-0.24, 0.20)	-0.04<br>(-0.13, 0.05)	-0.03<br>(-0.10, 0.04)	-0.01<br>(-0.08, 0.05)	-0.05<br>(-0.12, 0.02)	0.01<br>(-0.06, 0.07)	0.00<br>(-0.08, 0.08)	-0.03<br>(-0.14, 0.09)	Lard	
-0.15<br>(-0.35, 0.06)	0.00<br>(-0.03, 0.03)	0.01<br>(-0.04, 0.05)	0.00<br>(-0.21, 0.21)	-0.02<br>(-0.09, 0.05)	-0.01<br>(-0.04, 0.02)	0.01<br>(-0.02, 0.04)	-0.02<br>(-0.05, 0.00)	0.03<br>(-0.01, 0.07)	0.03<br>(-0.02, 0.07)	-0.00<br>(-0.09, 0.09)	0.02<br>(-0.05, 0.09)	Butter

The value below the oils/ solid fats corresponds to the difference in mean in HDL-cholesterol (mmol/l) between the column and the row (eg, the mean difference in average HDL-cholesterol between safflower oil and butter is -0.15 mmol/l).

Supplemental Table S19: Low risk of bias sensitivity analysis for triacylglycerols

Safflower oil												
-0.02<br>(-0.28, 0.23)	Sunflower oil											
-0.03<br>(-0.29, 0.23)	-0.00<br>(-0.05, 0.05)	Rapeseed oil										
-0.02<br>(-0.30, 0.26)	0.00<br>(-0.18, 0.19)	0.01<br>(-0.18, 0.20)	Hempseed oil									
-0.04<br>(-0.27, 0.19)	-0.02<br>(-0.12, 0.09)	-0.01<br>(-0.13, 0.11)	-0.02<br>(-0.17, 0.13)	Flaxseed oil								
-0.03<br>(-0.29, 0.24)	-0.00<br>(-0.07, 0.06)	0.00<br>(-0.07, 0.07)	-0.01<br>(-0.20, 0.19)	0.01<br>(-0.11, 0.13)	Corn oil							
-0.06<br>(-0.32, 0.20)	-0.04<br>(-0.08, 0.00)	-0.03<br>(-0.08, 0.02)	-0.04<br>(-0.23, 0.15)	-0.02<br>(-0.13, 0.09)	-0.03<br>(-0.08, 0.02)	Olive oil						
-0.02<br>(-0.28, 0.24)	0.00<br>(-0.04, 0.05)	0.01<br>(-0.05, 0.06)	-0.00<br>(-0.19, 0.19)	0.02<br>(-0.10, 0.13)	0.01<br>(-0.05, 0.06)	0.04<br>(0.01, 0.06)	Soybean oil					
-0.01<br>(-0.27, 0.25)	0.01<br>(-0.04, 0.06)	0.02<br>(-0.04, 0.07)	0.01<br>(-0.18, 0.20)	0.03<br>(-0.09, 0.14)	0.02<br>(-0.06, 0.09)	0.05<br>(-0.01, 0.10)	0.01<br>(-0.05, 0.07)	Palm oil				
-0.20<br>(-0.58, 0.18)	-0.18<br>(-0.46, 0.11)	-0.17<br>(-0.46, 0.12)	-0.18<br>(-0.52, 0.16)	-0.16<br>(-0.46, 0.14)	-0.17<br>(-0.46, 0.12)	-0.14<br>(-0.42, 0.14)	-0.18<br>(-0.46, 0.10)	-0.19<br>(-0.48, 0.10)	Coconut oil			
-0.07<br>(-0.37, 0.22)	-0.05<br>(-0.19, 0.09)	-0.05<br>(-0.18, 0.09)	-0.06<br>(-0.29, 0.17)	-0.04<br>(-0.21, 0.14)	-0.05<br>(-0.19, 0.09)	-0.01<br>(-0.15, 0.12)	-0.05<br>(-0.19, 0.08)	-0.06<br>(-0.21, 0.08)	0.12<br>(-0.19, 0.44)	Beef fat		
-0.06<br>(-0.37, 0.25)	-0.03<br>(-0.21, 0.14)	-0.03<br>(-0.20, 0.15)	-0.04<br>(-0.29, 0.21)	-0.02<br>(-0.22, 0.18)	-0.03<br>(-0.21, 0.15)	0.00<br>(-0.17, 0.17)	-0.04<br>(-0.21, 0.14)	-0.05<br>(-0.22, 0.12)	0.14<br>(-0.19, 0.47)	0.02<br>(-0.20, 0.23)	Lard	
-0.08<br>(-0.34, 0.18)	-0.05<br>(-0.10, -0.01)	-0.05<br>(-0.11, 0.01)	-0.06<br>(-0.25, 0.13)	-0.04<br>(-0.15, 0.08)	-0.05<br>(-0.10, 0.00)	-0.02<br>(-0.04, 0.01)	-0.06<br>(-0.08, -0.03)	-0.07<br>(-0.13, -0.01)	0.12<br>(-0.16, 0.40)	-0.00<br>(-0.14, 0.13)	-0.02<br>(-0.19, 0.15)	Butter

The value below the oils/ solid fats corresponds to the difference in mean in triacyglycerols (mmol/l) between the column and the row (eg, the mean difference in average triacylglycerols between safflower oil and butter is -0.08 mmol/l).

Supplemental Table S20: Sensitivity analysis for total cholesterol including only trials were oils/solid fats were provided by investigators

Safflower oil												
-0.11<br>(-0.32, 0.09)	Sunflower oil											
-0.05<br>(-0.27, 0.17)	0.06<br>(-0.06, 0.19)	Rapeseed oil										
-0.16<br>(-0.69, 0.37)	-0.04<br>(-0.56, 0.48)	-0.11<br>(-0.63, 0.42)	Hempseed oil									
-0.18<br>(-0.58, 0.22)	-0.07<br>(-0.45, 0.31)	-0.13<br>(-0.52, 0.26)	-0.02<br>(-0.38, 0.33)	Flaxseed oil								
-0.10<br>(-0.30, 0.11)	0.02<br>(-0.08, 0.12)	-0.05<br>(-0.18, 0.09)	0.06<br>(-0.46, 0.58)	0.09<br>(-0.30, 0.47)	Corn oil							
-0.21<br>(-0.40, -0.02)	-0.10<br>(-0.18, -0.02)	-0.16<br>(-0.28, -0.04)	-0.05<br>(-0.57, 0.46)	-0.03<br>(-0.40, 0.35)	-0.11<br>(-0.20, -0.02)	Olive oil						
-0.17<br>(-0.37, 0.03)	-0.06<br>(-0.15, 0.04)	-0.12<br>(-0.26, 0.02)	-0.01<br>(-0.53, 0.51)	0.01<br>(-0.37, 0.39)	-0.07<br>(-0.18, 0.03)	0.04<br>(-0.05, 0.13)	Soybean oil					
-0.21<br>(-0.41, -0.02)	-0.10<br>(-0.18, -0.02)	-0.16<br>(-0.29, -0.03)	-0.06<br>(-0.57, 0.46)	-0.03<br>(-0.41, 0.35)	-0.12<br>(-0.22, -0.01)	-0.00<br>(-0.08, 0.08)	-0.04<br>(-0.13, 0.05)	Palm oil				
-0.31<br>(-0.46, -0.15)	-0.19<br>(-0.34, -0.05)	-0.26<br>(-0.43, -0.09)	-0.15<br>(-0.67, 0.37)	-0.13<br>(-0.51, 0.26)	-0.21<br>(-0.36, -0.06)	-0.10<br>(-0.23, 0.04)	-0.14<br>(-0.28, 0.01)	-0.09<br>(-0.24, 0.05)	Coconut oil			
-0.22<br>(-0.40, -0.05)	-0.11<br>(-0.29, 0.07)	-0.17<br>(-0.37, 0.02)	-0.07<br>(-0.60, 0.47)	-0.04<br>(-0.44, 0.36)	-0.13<br>(-0.31, 0.06)	-0.01<br>(-0.19, 0.16)	-0.05<br>(-0.24, 0.13)	-0.01<br>(-0.19, 0.17)	0.08<br>(-0.07, 0.24)	Beef fat		
-0.40<br>(-0.67, -0.13)	-0.29<br>(-0.49, -0.08)	-0.35<br>(-0.58, -0.12)	-0.24<br>(-0.79, 0.31)	-0.22<br>(-0.64, 0.21)	-0.30<br>(-0.52, -0.09)	-0.19<br>(-0.39, 0.02)	-0.23<br>(-0.43, -0.02)	-0.19<br>(-0.39, 0.01)	-0.09<br>(-0.33, 0.15)	-0.18<br>(-0.44, 0.09)	Lard	
-0.49<br>(-0.69, -0.28)	-0.37<br>(-0.48, -0.26)	-0.44<br>(-0.58, -0.29)	-0.33<br>(-0.85, 0.19)	-0.30<br>(-0.69, 0.08)	-0.39<br>(-0.50, -0.28)	-0.28<br>(-0.37, -0.18)	-0.31<br>(-0.42, -0.21)	-0.27<br>(-0.38, -0.16)	-0.18<br>(-0.33, -0.03)	-0.26<br>(-0.45, -0.07)	-0.09<br>(-0.30, 0.13)	Butter

The value below the oils/ solid fats corresponds to the difference in mean in total-cholesterol (mmol/l) between the column and the row (eg, the mean difference in average total-cholesterol between safflower oil and butter is -0.49 mmol/l).

Supplemental Table S21: Sensitivity analysis for LDL-cholesterol including only trials were oils/ solid fats were provided by investigators

Safflower oil												
-0.08 (-0.30, 0.15)	Sunflower oil											
-0.05 (-0.29, 0.19)	0.02 (-0.10, 0.15)	Rapeseed oil										
0.04 (-0.53, 0.62)	0.12 (-0.43, 0.67)	0.10 (-0.46, 0.65)	Hempseed oil									
0.01 (-0.39, 0.41)	0.09 (-0.27, 0.45)	0.06 (-0.31, 0.44)	-0.03 (-0.44, 0.38)	Flaxseed oil								
-0.09 (-0.32, 0.14)	-0.02 (-0.12, 0.09)	-0.04 (-0.18, 0.10)	-0.14 (-0.69, 0.41)	-0.10 (-0.47, 0.26)	Corn oil							
-0.16 (-0.38, 0.05)	-0.09 (-0.17, -0.01)	-0.11 (-0.23, 0.01)	-0.21 (-0.75, 0.34)	-0.17 (-0.53, 0.18)	-0.07 (-0.16, 0.02)	Olive oil						
-0.15 (-0.38, 0.07)	-0.08 (-0.17, 0.02)	-0.10 (-0.24, 0.04)	-0.20 (-0.75, 0.35)	-0.16 (-0.53, 0.20)	-0.06 (-0.16, 0.04)	0.01 (-0.08, 0.10)	Soybean oil					
-0.17 (-0.39, 0.06)	-0.09 (-0.17, -0.01)	-0.11 (-0.24, 0.02)	-0.21 (-0.76, 0.34)	-0.18 (-0.54, 0.19)	-0.07 (-0.18, 0.03)	-0.00 (-0.08, 0.08)	-0.01 (-0.10, 0.08)	Palm oil				
-0.19 (-0.37, -0.00)	-0.11 (-0.26, 0.04)	-0.14 (-0.31, 0.04)	-0.23 (-0.79, 0.33)	-0.20 (-0.58, 0.18)	-0.10 (-0.25, 0.06)	-0.02 (-0.17, 0.12)	-0.04 (-0.19, 0.12)	-0.02 (-0.17, 0.13)	Coconut oil			
-0.14 (-0.35, 0.08)	-0.06 (-0.26, 0.15)	-0.08 (-0.30, 0.14)	-0.18 (-0.75, 0.39)	-0.15 (-0.55, 0.25)	-0.04 (-0.25, 0.17)	0.03 (-0.17, 0.23)	0.02 (-0.19, 0.23)	0.03 (-0.17, 0.23)	0.05 (-0.13, 0.24)	Beef fat		
-0.33 (-0.61, -0.05)	-0.25 (-0.44, -0.06)	-0.28 (-0.49, -0.06)	-0.37 (-0.95, 0.20)	-0.34 (-0.74, 0.06)	-0.24 (-0.44, -0.04)	-0.17 (-0.35, 0.02)	-0.18 (-0.37, 0.01)	-0.16 (-0.35, 0.02)	-0.14 (-0.37, 0.09)	-0.19 (-0.46, 0.07)	Lard	
-0.43 (-0.66, -0.20)	-0.35 (-0.46, -0.23)	-0.37 (-0.52, -0.22)	-0.47 (-1.02, 0.08)	-0.44 (-0.80, -0.07)	-0.33 (-0.45, -0.22)	-0.26 (-0.36, -0.16)	-0.27 (-0.38, -0.17)	-0.26 (-0.37, -0.15)	-0.24 (-0.39, -0.08)	-0.29 (-0.50, -0.08)	-0.10 (-0.30, 0.11)	butter

The value below the oils/ solid fats corresponds to the difference in mean in LDL-cholesterol (mmol/l) between the column and the row (eg, the mean difference in average LDL-cholesterol between safflower oil and butter is -0.43 mmol/l).

Supplemental Table S22: Sensitivity analysis for HDL-cholesterol including only trials were oils/ solid fats were provided by investigators

Safflower oil												
-0.05<br>(-0.10, -0.00)	Sunflower oil											
-0.05<br>(-0.11, 0.01)	0.00<br>(-0.03, 0.04)	Rapeseed oil										
-0.12<br>(-0.34, 0.11)	-0.06<br>(-0.29, 0.16)	-0.06<br>(-0.29, 0.16)	Hempseed oil									
-0.09<br>(-0.20, 0.02)	-0.04<br>(-0.15, 0.07)	-0.04<br>(-0.15, 0.07)	0.02<br>(-0.17, 0.22)	Flaxseed oil								
-0.03<br>(-0.08, 0.02)	0.02<br>(-0.01, 0.04)	0.02<br>(-0.02, 0.06)	0.08<br>(-0.14, 0.31)	0.06<br>(-0.05, 0.17)	Corn oil							
-0.05<br>(-0.10, -0.00)	0.00<br>(-0.02, 0.02)	0.00<br>(-0.03, 0.04)	0.06<br>(-0.16, 0.29)	0.04<br>(-0.07, 0.15)	-0.02<br>(-0.04, 0.01)	Olive oil						
-0.02<br>(-0.07, 0.03)	0.03<br>(0.01, 0.06)	0.03<br>(-0.01, 0.07)	0.10<br>(-0.13, 0.32)	0.07<br>(-0.04, 0.18)	0.02<br>(-0.01, 0.04)	0.03<br>(0.01, 0.05)	Soybean oil					
-0.07<br>(-0.12, -0.02)	-0.02<br>(-0.04, 0.00)	-0.02<br>(-0.05, 0.02)	0.05<br>(-0.18, 0.27)	0.02<br>(-0.09, 0.13)	-0.04<br>(-0.06, -0.01)	-0.02<br>(-0.04, 0.00)	-0.05<br>(-0.07, -0.03)	Palm oil				
-0.08<br>(-0.12, -0.04)	-0.03<br>(-0.06, 0.00)	-0.03<br>(-0.08, 0.01)	0.03<br>(-0.19, 0.26)	0.01<br>(-0.10, 0.11)	-0.05<br>(-0.08, -0.02)	-0.03<br>(-0.06, -0.00)	-0.06<br>(-0.10, -0.03)	-0.01<br>(-0.05, 0.02)	Coconut oil			
-0.07<br>(-0.11, -0.03)	-0.02<br>(-0.07, 0.03)	-0.02<br>(-0.08, 0.03)	0.04<br>(-0.18, 0.27)	0.02<br>(-0.09, 0.13)	-0.04<br>(-0.09, 0.01)	-0.02<br>(-0.07, 0.02)	-0.05<br>(-0.10, -0.01)	-0.00<br>(-0.05, 0.04)	0.01<br>(-0.03, 0.05)	Beef fat		
-0.05<br>(-0.11, 0.01)	-0.00<br>(-0.04, 0.04)	-0.00<br>(-0.05, 0.05)	0.06<br>(-0.17, 0.29)	0.04<br>(-0.08, 0.15)	-0.02<br>(-0.06, 0.02)	-0.00<br>(-0.04, 0.04)	-0.04<br>(-0.07, 0.00)	0.02<br>(-0.02, 0.05)	0.03<br>(-0.02, 0.08)	0.02<br>(-0.04, 0.08)	Lard	
-0.04<br>(-0.09, 0.01)	0.01<br>(-0.02, 0.04)	0.01<br>(-0.03, 0.05)	0.07<br>(-0.15, 0.30)	0.05<br>(-0.06, 0.16)	-0.01<br>(-0.04, 0.02)	0.01<br>(-0.02, 0.04)	-0.02<br>(-0.05, 0.00)	0.03<br>(0.00, 0.06)	0.04<br>(0.01, 0.08)	0.03<br>(-0.02, 0.08)	0.01<br>(-0.03, 0.06)	Butter

The value below the oils/ solid fats corresponds to the difference in mean in HDL-cholesterol (mmol/l) between the column and the row (eg, the mean difference in average HDL-cholesterol between safflower oil and butter is -0.04 mmol/l).

Supplemental Table S23: Sensitivity analysis for triacylglycerols including only trials were oils/ solid fats were provided by investigators												
Safflower oil												
-0.01<br>(-0.08, 0.06)	Sunflower oil											
-0.01<br>(-0.09, 0.06)	-0.00<br>(-0.05, 0.04)	Rapeseed oil										
0.02<br>(-0.16, 0.20)	0.03<br>(-0.15, 0.21)	0.03<br>(-0.15, 0.22)	Hempseed oil									
-0.00<br>(-0.11, 0.10)	0.01<br>(-0.09, 0.10)	0.01<br>(-0.09, 0.12)	-0.02<br>(-0.17, 0.13)	Flaxseed oil								
-0.00<br>(-0.08, 0.08)	0.01<br>(-0.05, 0.06)	0.01<br>(-0.05, 0.07)	-0.02<br>(-0.21, 0.16)	-0.00<br>(-0.11, 0.11)	Corn oil							
-0.04<br>(-0.10, 0.03)	-0.03<br>(-0.06, 0.00)	-0.02<br>(-0.07, 0.02)	-0.06<br>(-0.24, 0.12)	-0.04<br>(-0.13, 0.06)	-0.03<br>(-0.08, 0.01)	Olive oil						
0.00<br>(-0.07, 0.07)	0.01<br>(-0.03, 0.05)	0.01<br>(-0.04, 0.06)	-0.02<br>(-0.20, 0.16)	0.00<br>(-0.10, 0.10)	0.00<br>(-0.05, 0.05)	0.04<br>(0.01, 0.06)	Soybean oil					
0.00<br>(-0.06, 0.07)	0.01<br>(-0.03, 0.05)	0.01<br>(-0.04, 0.06)	-0.02<br>(-0.20, 0.16)	0.00<br>(-0.10, 0.10)	0.00<br>(-0.06, 0.07)	0.04<br>(-0.01, 0.08)	0.00<br>(-0.04, 0.05)	Palm oil				
-0.04<br>(-0.10, 0.01)	-0.03<br>(-0.07, 0.01)	-0.03<br>(-0.08, 0.02)	-0.06<br>(-0.24, 0.12)	-0.04<br>(-0.14, 0.06)	-0.04<br>(-0.10, 0.02)	-0.01<br>(-0.04, 0.03)	-0.04<br>(-0.08, -0.00)	-0.04<br>(-0.08, -0.01)	Coconut oil			
-0.08<br>(-0.14, -0.03)	-0.07<br>(-0.14, -0.01)	-0.07<br>(-0.14, -0.00)	-0.10<br>(-0.29, 0.08)	-0.08<br>(-0.19, 0.02)	-0.08<br>(-0.16, -0.01)	-0.05<br>(-0.11, 0.01)	-0.08<br>(-0.15, -0.02)	-0.09<br>(-0.14, -0.03)	-0.04<br>(-0.09, 0.01)	Beef fat		
-0.02<br>(-0.13, 0.09)	-0.01<br>(-0.11, 0.09)	-0.01<br>(-0.11, 0.10)	-0.04<br>(-0.24, 0.16)	-0.02<br>(-0.15, 0.12)	-0.02<br>(-0.12, 0.09)	0.02<br>(-0.08, 0.11)	-0.02<br>(-0.12, 0.08)	-0.02<br>(-0.12, 0.08)	0.02<br>(-0.08, 0.12)	0.06<br>(-0.04, 0.17)	Lard	
-0.05<br>(-0.12, 0.01)	-0.05<br>(-0.08, -0.01)	-0.04<br>(-0.09, 0.01)	-0.07<br>(-0.25, 0.11)	-0.05<br>(-0.15, 0.05)	-0.05<br>(-0.10, -0.00)	-0.02<br>(-0.04, 0.01)	-0.05<br>(-0.08, -0.03)	-0.06<br>(-0.10, -0.01)	-0.01<br>(-0.05, 0.03)	0.03<br>(-0.03, 0.09)	-0.04<br>(-0.13, 0.06)	Butter

The value below the oils/ solid fats corresponds to the difference in mean in triacyglycerols (mmol/l) between the column and the row (eg, the mean difference in average triacylglycerols between safflower oil and butter is -0.05 mmol/l).

Supplemental Table S24: Sensitivity analysis for total cholesterol including only trials with healthy participants

Safflower oil												
-0.06 (-0.29, 0.17)	Sunflower oil											
-0.06 (-0.34, 0.22)	-0.00 (-0.20, 0.20)	Rapessed oil										
0.10 (-0.52, 0.71)	0.15 (-0.43, 0.74)	0.15 (-0.45, 0.76)	Hempseed oil									
0.07 (-0.44, 0.58)	0.13 (-0.34, 0.60)	0.13 (-0.37, 0.63)	-0.02 (-0.37, 0.32)	Flaxseed oil								
-0.03 (-0.29, 0.23)	0.03 (-0.11, 0.17)	0.03 (-0.20, 0.26)	-0.13 (-0.72, 0.46)	-0.10 (-0.58, 0.38)	Corn oil							
-0.20 (-0.42, 0.01)	-0.15 (-0.26, -0.04)	-0.14 (-0.34, 0.05)	-0.30 (-0.87, 0.27)	-0.28 (-0.73, 0.18)	-0.17 (-0.33, -0.02)	Olive oil						
-0.16 (-0.39, 0.07)	-0.10 (-0.21, 0.01)	-0.10 (-0.30, 0.09)	-0.26 (-0.84, 0.33)	-0.23 (-0.70, 0.24)	-0.13 (-0.27, 0.01)	0.04 (-0.06, 0.14)	Soybean oil					
-0.17 (-0.38, 0.05)	-0.11 (-0.22, 0.00)	-0.11 (-0.29, 0.08)	-0.26 (-0.84, 0.32)	-0.24 (-0.70, 0.23)	-0.13 (-0.29, 0.02)	0.04 (-0.06, 0.13)	-0.01 (-0.09, 0.08)	Palm oil				
-0.31 (-0.46, -0.15)	-0.25 (-0.42, -0.07)	-0.25 (-0.48, -0.01)	-0.40 (-1.00, 0.19)	-0.38 (-0.86, 0.11)	-0.28 (-0.48, -0.07)	-0.10 (-0.26, 0.05)	-0.15 (-0.31, 0.02)	-0.14 (-0.30, 0.02)	Coconut oil			
-0.16 (-0.33, 0.02)	-0.10 (-0.34, 0.15)	-0.10 (-0.39, 0.19)	-0.25 (-0.87, 0.37)	-0.23 (-0.74, 0.28)	-0.13 (-0.39, 0.14)	0.05 (-0.18, 0.28)	0.00 (-0.24, 0.24)	0.01 (-0.23, 0.24)	0.15 (-0.02, 0.32)	Beef fat		
-0.37 (-0.66, -0.08)	-0.31 (-0.53, -0.10)	-0.31 (-0.58, -0.05)	-0.47 (-1.07, 0.14)	-0.44 (-0.94, 0.06)	-0.34 (-0.58, -0.10)	-0.17 (-0.37, 0.04)	-0.21 (-0.41, -0.01)	-0.20 (-0.40, -0.01)	-0.06 (-0.31, 0.18)	-0.21 (-0.51, 0.09)	Lard	
-0.47 (-0.70, -0.24)	-0.41 (-0.55, -0.28)	-0.41 (-0.63, -0.20)	-0.57 (-1.15, 0.02)	-0.54 (-1.02, -0.07)	-0.44 (-0.61, -0.28)	-0.27 (-0.39, -0.15)	-0.31 (-0.44, -0.19)	-0.31 (-0.44, -0.18)	-0.17 (-0.34, 0.01)	-0.32 (-0.56, -0.07)	-0.10 (-0.33, 0.12)	Butter

The value below the oils/ solid fats corresponds to the difference in mean in total-cholesterol (mmol/l) between the column and the row (eg, the mean difference in average total-cholesterol between safflower oil and butter is -0.47 mmol/l).

Supplemental Table S25: Sensitivity analysis for LDL-cholesterol including only trials with healthy participants

Safflower oil												
-0.04<br>(-0.35, 0.27)	Sunflower oil											
-0.07<br>(-0.45, 0.31)	-0.04<br>(-0.31, 0.24)	Rapeseed oil										
0.14<br>(-0.49, 0.77)	0.18<br>(-0.39, 0.74)	0.21<br>(-0.40, 0.83)	Hempseed oil									
0.11<br>(-0.37, 0.58)	0.14<br>(-0.24, 0.53)	0.18<br>(-0.28, 0.64)	-0.03<br>(-0.45, 0.38)	Flaxseed oil								
-0.02<br>(-0.34, 0.29)	0.01<br>(-0.13, 0.16)	0.05<br>(-0.24, 0.34)	-0.16<br>(-0.74, 0.41)	-0.13<br>(-0.52, 0.26)	Corn oil							
-0.14<br>(-0.44, 0.15)	-0.11<br>(-0.24, 0.02)	-0.07<br>(-0.34, 0.19)	-0.28<br>(-0.84, 0.28)	-0.25<br>(-0.63, 0.12)	-0.12<br>(-0.26, 0.02)	Olive oil						
-0.16<br>(-0.46, 0.15)	-0.12<br>(-0.25, 0.01)	-0.08<br>(-0.35, 0.18)	-0.30<br>(-0.87, 0.27)	-0.26<br>(-0.65, 0.12)	-0.13<br>(-0.28, 0.02)	-0.01<br>(-0.13, 0.11)	Soybean oil					
-0.13<br>(-0.42, 0.16)	-0.10<br>(-0.23, 0.03)	-0.06<br>(-0.30, 0.18)	-0.27<br>(-0.84, 0.30)	-0.24<br>(-0.63, 0.15)	-0.11<br>(-0.26, 0.05)	0.01<br>(-0.10, 0.12)	0.02<br>(-0.08, 0.13)	Palm oil				
-0.21<br>(-0.41, -0.01)	-0.17<br>(-0.41, 0.07)	-0.14<br>(-0.46, 0.19)	-0.35<br>(-0.95, 0.25)	-0.32<br>(-0.75, 0.11)	-0.18<br>(-0.43, 0.06)	-0.06<br>(-0.28, 0.15)	-0.05<br>(-0.28, 0.18)	-0.08<br>(-0.29, 0.14)	Coconut oil			
-0.08<br>(-0.32, 0.16)	-0.04<br>(-0.38, 0.29)	-0.01<br>(-0.41, 0.39)	-0.22<br>(-0.86, 0.42)	-0.19<br>(-0.68, 0.30)	-0.06<br>(-0.40, 0.29)	0.06<br>(-0.25, 0.38)	0.08<br>(-0.25, 0.41)	0.05<br>(-0.26, 0.37)	0.13<br>(-0.10, 0.36)	Beef fat		
-0.31<br>(-0.65, 0.03)	-0.27<br>(-0.49, -0.05)	-0.24<br>(-0.54, 0.07)	-0.45<br>(-1.04, 0.14)	-0.42<br>(-0.84, 0.00)	-0.29<br>(-0.52, -0.05)	-0.17<br>(-0.36, 0.03)	-0.15<br>(-0.35, 0.05)	-0.18<br>(-0.37, 0.01)	-0.10<br>(-0.38, 0.18)	-0.23<br>(-0.59, 0.13)	Lard	
-0.44<br>(-0.76, -0.11)	-0.40<br>(-0.57, -0.23)	-0.37<br>(-0.66, -0.07)	-0.58<br>(-1.16, -0.00)	-0.55<br>(-0.95, -0.14)	-0.42<br>(-0.59, -0.24)	-0.29<br>(-0.45, -0.14)	-0.28<br>(-0.44, -0.12)	-0.31<br>(-0.48, -0.14)	-0.23<br>(-0.49, 0.03)	-0.36<br>(-0.71, -0.01)	-0.13<br>(-0.37, 0.11)	Butter

The value below the oils/ solid fats corresponds to the difference in mean in LDL-cholesterol (mmol/l) between the column and the row (eg, the mean difference in average LDL-cholesterol between safflower oil and butter is -0.44 mmol/l).

Supplemental Table S26: Sensitivity analysis for HDL-cholesterol including only trials with healthy participants

Safflower oil												
-0.02 (-0.09, 0.04)	Sunflower oil											
-0.06 (-0.14, 0.02)	-0.04 (-0.10, 0.03)	Rapeseed oil										
-0.03 (-0.31, 0.24)	-0.01 (-0.28, 0.26)	0.03 (-0.25, 0.30)	Hempseed oil									
-0.01 (-0.21, 0.19)	0.01 (-0.18, 0.20)	0.05 (-0.15, 0.25)	0.02 (-0.17, 0.22)	Flaxseed oil								
-0.01 (-0.08, 0.06)	0.01 (-0.03, 0.05)	0.05 (-0.02, 0.12)	0.02 (-0.25, 0.30)	-0.00 (-0.19, 0.19)	Corn oil							
-0.04 (-0.10, 0.02)	-0.01 (-0.04, 0.01)	0.02 (-0.04, 0.08)	-0.00 (-0.28, 0.27)	-0.03 (-0.22, 0.16)	-0.03 (-0.07, 0.02)	Olive oil						
-0.01 (-0.07, 0.06)	0.02 (-0.02, 0.05)	0.05 (-0.01, 0.11)	0.03 (-0.25, 0.30)	0.00 (-0.19, 0.19)	0.00 (-0.04, 0.04)	0.03 (0.00, 0.06)	Soybean oil					
-0.06 (-0.13, -0.00)	-0.04 (-0.07, -0.01)	-0.01 (-0.06, 0.05)	-0.03 (-0.30, 0.24)	-0.06 (-0.24, 0.13)	-0.05 (-0.10, -0.01)	-0.03 (-0.05, -0.00)	-0.06 (-0.08, -0.03)	Palm oil				
-0.08 (-0.12, -0.03)	-0.05 (-0.10, -0.01)	-0.02 (-0.09, 0.05)	-0.04 (-0.32, 0.23)	-0.07 (-0.26, 0.13)	-0.06 (-0.11, -0.02)	-0.04 (-0.08, 0.00)	-0.07 (-0.11, -0.02)	-0.01 (-0.05,0.03)	Coconut oil			
-0.07 (-0.12, -0.03)	-0.05 (-0.11, 0.01)	-0.02 (-0.10, 0.07)	-0.04 (-0.32, 0.24)	-0.07 (-0.26, 0.13)	-0.06 (-0.13, 0.00)	-0.04 (-0.10, 0.02)	-0.07 (-0.13, -0.00)	-0.01 (-0.07,0.05)	0.00 (-0.04, 0.04)	Beef fat		
-0.05 (-0.12, 0.03)	-0.02 (-0.07, 0.02)	0.01 (-0.06, 0.08)	-0.01 (-0.29, 0.26)	-0.04 (-0.23, 0.16)	-0.04 (-0.09, 0.02)	-0.01 (-0.05, 0.03)	-0.04 (-0.08, 0.00)	0.02 (-0.02,0.06)	0.03 (-0.03, 0.09)	0.03 (-0.04, 0.10)	Lard	
-0.01 (-0.08, 0.06)	0.02 (-0.03, 0.06)	0.05 (-0.02, 0.12)	0.03 (-0.25, 0.30)	0.00 (-0.19, 0.19)	0.00 (-0.05, 0.06)	0.03 (-0.01, 0.07)	0.00 (-0.04, 0.04)	0.06 (0.01,0.10)	0.07 (0.01, 0.12)	0.07 (-0.00, 0.14)	0.04 (-0.02, 0.10)	Butter

The value below the oils/ solid fats corresponds to the difference in mean in HDL-cholesterol (mmol/l) between the column and the row (eg, the mean difference in average HDL-cholesterol between safflower oil and butter is -0.01 mmol/l).

Supplemental Table S27: Sensitivity analysis for triacylglycerols including only trials with healthy participants

Safflower oil												
0.01<br>(-0.07, 0.08)	Sunflower oil											
0.00<br>(-0.08, 0.09)	-0.01<br>(-0.06, 0.05)	Rapeseed oil										
0.14<br>(-0.13, 0.40)	0.13<br>(-0.13, 0.39)	0.14<br>(-0.13, 0.40)	Hempseed oil									
0.12<br>(-0.10, 0.33)	0.11<br>(-0.10, 0.32)	0.12<br>(-0.10, 0.33)	-0.02<br>(-0.17, 0.13)	Flaxseed oil								
0.15<br>(-0.03, 0.34)	0.15<br>(-0.02, 0.32)	0.15<br>(-0.02, 0.33)	0.02<br>(-0.28, 0.32)	0.04<br>(-0.22, 0.29)	Corn oil							
-0.03<br>(-0.10, 0.04)	-0.03<br>(-0.08, 0.01)	-0.03<br>(-0.09, 0.03)	-0.16<br>(-0.42, 0.09)	-0.14<br>(-0.35, 0.06)	-0.18<br>(-0.35, -0.01)	Olive oil						
0.01<br>(-0.06, 0.08)	0.00<br>(-0.04, 0.05)	0.01<br>(-0.05, 0.07)	-0.12<br>(-0.38, 0.13)	-0.10<br>(-0.31, 0.10)	-0.14<br>(-0.31, 0.03)	0.04<br>(0.01, 0.06)	Soybean oil					
0.01<br>(-0.06, 0.08)	0.00<br>(-0.04, 0.05)	0.01<br>(-0.05, 0.07)	-0.12<br>(-0.38, 0.13)	-0.10<br>(-0.31, 0.11)	-0.14<br>(-0.32, 0.03)	0.04<br>(-0.01, 0.09)	0.00<br>(-0.05, 0.05)	Palm oil				
-0.03<br>(-0.09, 0.03)	-0.04<br>(-0.09, 0.01)	-0.03<br>(-0.10, 0.03)	-0.17<br>(-0.43, 0.09)	-0.15<br>(-0.36, 0.06)	-0.19<br>(-0.36, -0.02)	-0.01<br>(-0.05, 0.03)	-0.04<br>(-0.09, -0.00)	-0.04<br>(-0.08, -0.01)	Coconut oil			
-0.08<br>(-0.14, -0.03)	-0.09<br>(-0.16, -0.02)	-0.08<br>(-0.17, -0.00)	-0.22<br>(-0.48, 0.04)	-0.20<br>(-0.41, 0.01)	-0.24<br>(-0.42, -0.06)	-0.06<br>(-0.12, 0.01)	-0.10<br>(-0.16, -0.03)	-0.10<br>(-0.16, -0.03)	-0.05<br>(-0.10, 0.00)	Beef fat		
-0.01<br>(-0.12, 0.11)	-0.02<br>(-0.12, 0.09)	-0.01<br>(-0.12, 0.10)	-0.14<br>(-0.42, 0.13)	-0.12<br>(-0.35, 0.10)	-0.16<br>(-0.36, 0.03)	0.02<br>(-0.08, 0.12)	-0.02<br>(-0.12, 0.08)	-0.02<br>(-0.12, 0.08)	0.02<br>(-0.07, 0.12)	0.07<br>(-0.04, 0.19)	Lard	
-0.04<br>(-0.12, 0.03)	-0.05<br>(-0.10, -0.00)	-0.04<br>(-0.11, 0.02)	-0.18<br>(-0.43, 0.08)	-0.16<br>(-0.36, 0.05)	-0.20<br>(-0.36, -0.03)	-0.02<br>(-0.04, 0.01)	-0.05<br>(-0.08, -0.03)	-0.06<br>(-0.11, -0.00)	-0.01<br>(-0.06, 0.03)	0.04<br>(-0.03, 0.11)	-0.03<br>(-0.13, 0.06)	Butter

The value below the oils/ solid fats corresponds to the difference in mean in triacyglycerols (mmol/l) between the column and the row (eg, the mean difference in average triacylglycerols between safflower oil and butter is -0.04 mmol/l).

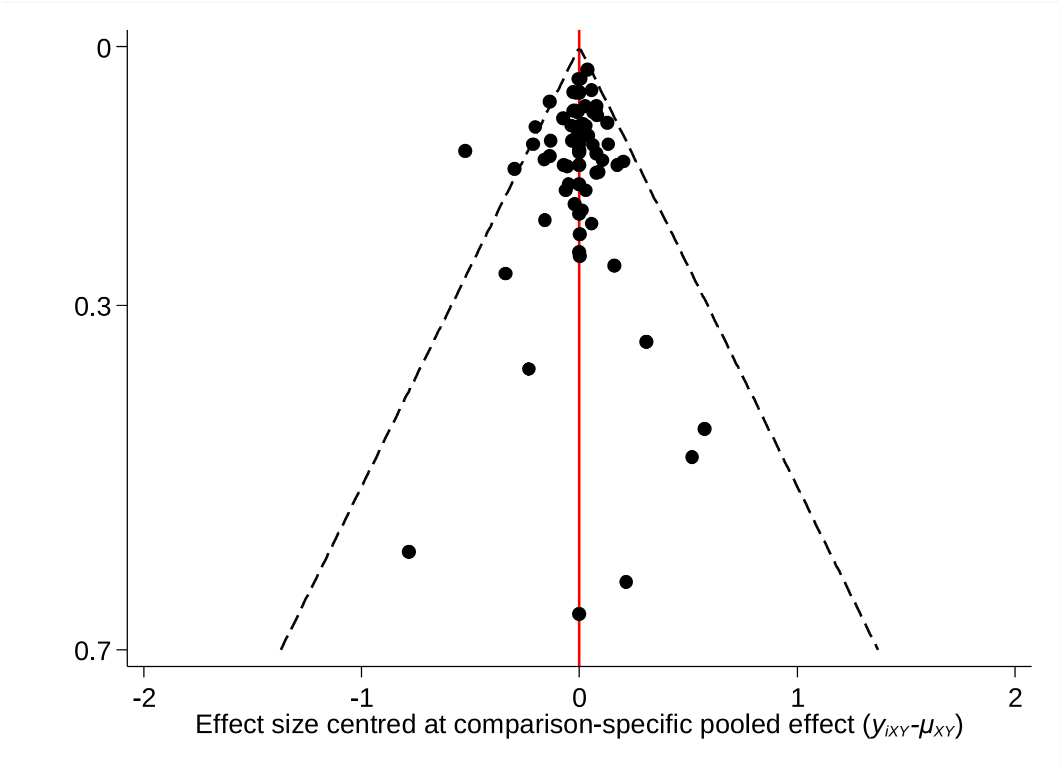

Supplemental Figure S11: Comparison-adjusted funnel plot for total-cholesterol including all studies.

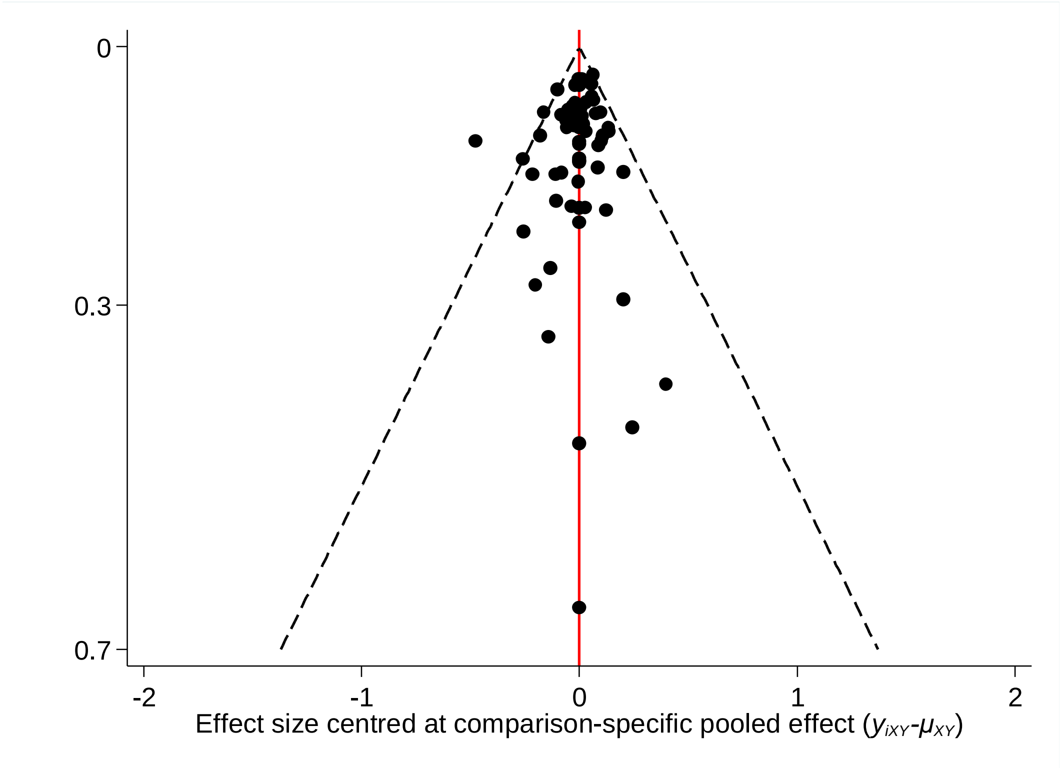

Supplemental Figure S12: Comparison-adjusted funnel plot for LDL cholesterol including all studies.

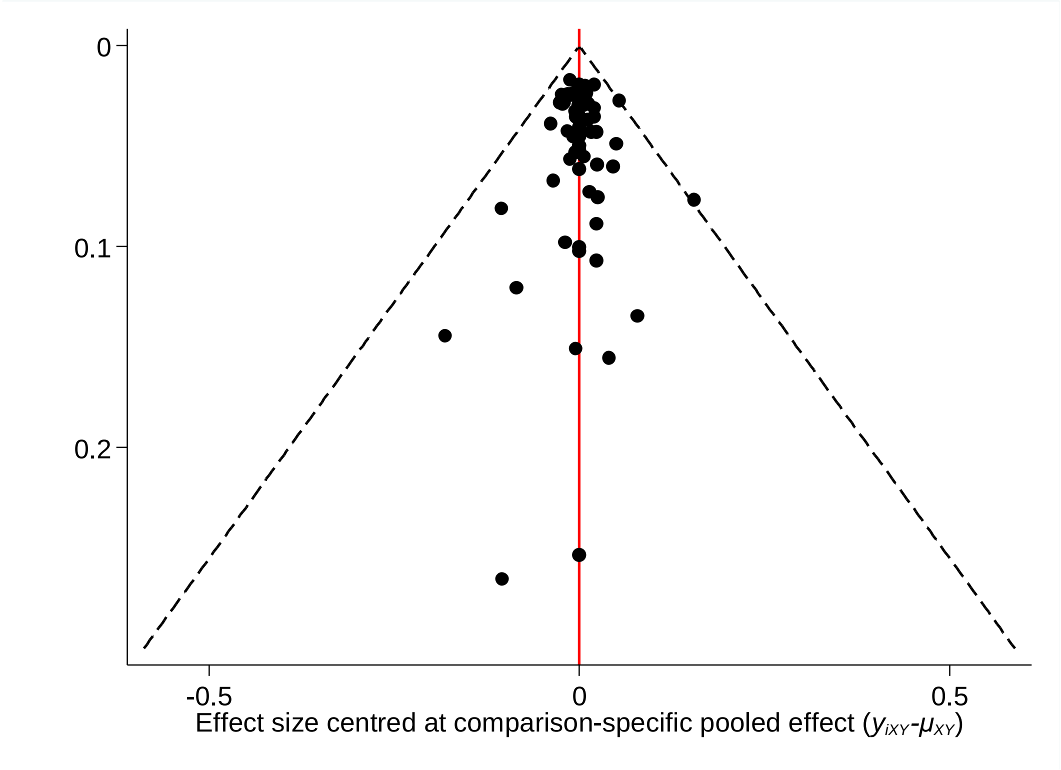

Supplemental Figure S13: Comparison-adjusted funnel plot for HDL-cholesterol including all studies.

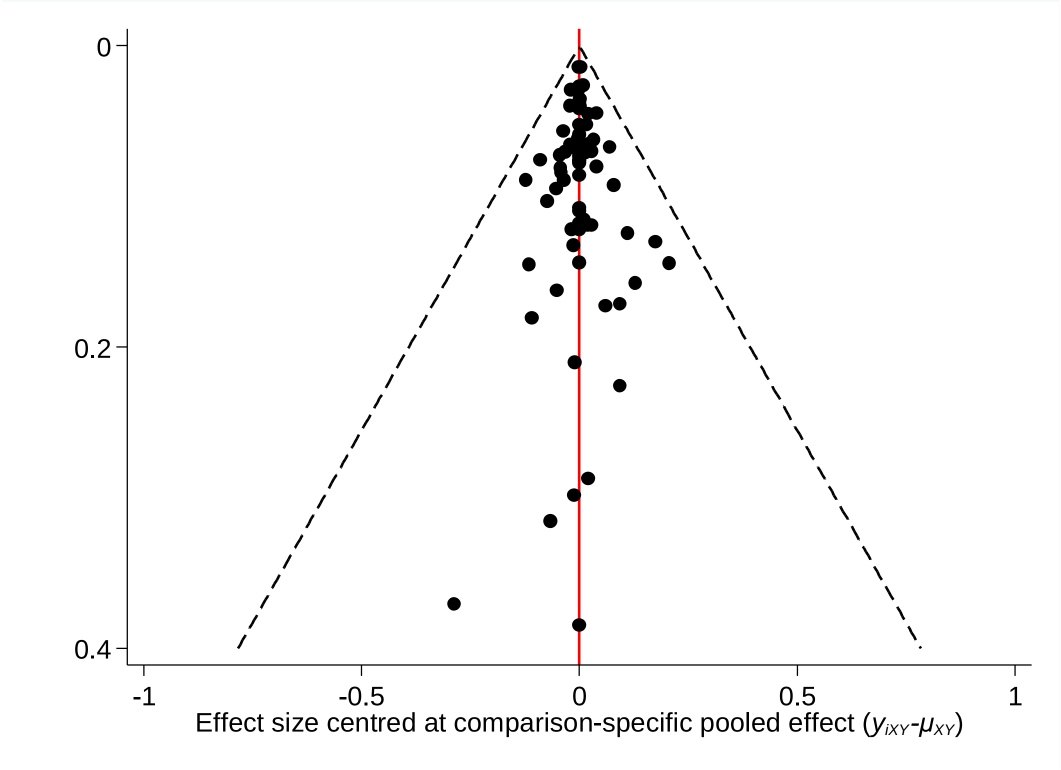

Supplemental Figure S14: Comparison-adjusted funnel plot for triacylglycerols including all studies.

Comparison	Number of studies	Study Limitations	Imprecision	Heterogeneity	Incoherence	Indirectness	Rating
**Mixed evidence**							
**1 vs 2**	5	No concerns	No concerns	Some concerns	No concerns	No concerns	Moderate
**1 vs 3**	4	No concerns	Some concerns	No concerns	No concerns	No concerns	Moderate
**1 vs 4**	2	Some concerns	No concerns	Some concerns	No concerns	No concerns	Moderate
**1 vs 6**	1	Some concerns	Some concerns	No concerns	No concerns	No concerns	Moderate
**1 vs 7**	6	No concerns	No concerns	Some concerns	No concerns	No concerns	Moderate
**1 vs 8**	5	Some concerns	No concerns	Some concerns	No concerns	No concerns	Moderate
**1 vs 9**	2	Some concerns	No concerns	Some concerns	No concerns	No concerns	Moderate
**1 vs 10**	5	Some concerns	Some concerns	No concerns	Some concerns	No concerns	Low
**1 vs 12**	1	Some concerns	No concerns	Major concerns	No concerns	No concerns	Very low
**1 vs 13**	1	Some concerns	Some concerns	No concerns	No concerns	No concerns	Moderate
**2 vs 3**	3	No concerns	No concerns	Some concerns	No concerns	No concerns	Moderate
**2 vs 4**	1	No concerns	No concerns	Some concerns	No concerns	No concerns	Moderate
**2 vs 6**	2	No concerns	No concerns	Major concerns	No concerns	No concerns	Low
**2 vs 7**	2	No concerns	No concerns	Some concerns	No concerns	No concerns	Moderate
**2 vs 8**	7	No concerns	No concerns	Some concerns	No concerns	No concerns	Moderate
**3 vs 7**	1	No concerns	No concerns	Some concerns	No concerns	No concerns	Moderate
**3 vs 8**	1	No concerns	Some concerns	No concerns	No concerns	No concerns	Moderate
**4 vs 7**	2	No concerns	No concerns	Some concerns	No concerns	No concerns	Moderate
**4 vs 8**	4	Some concerns	No concerns	Some concerns	Some concerns	No concerns	Moderate
**4 vs 9**	1	Some concerns	Some concerns	No concerns	Some concerns	No concerns	Low
**5 vs 6**	2	Some concerns	Some concerns	No concerns	No concerns	No concerns	Moderate
**5 vs 9**	2	Some concerns	Some concerns	No concerns	No concerns	No concerns	Moderate
**6 vs 7**	1	Some concerns	No concerns	Major concerns	No concerns	No concerns	Very low
**8 vs 9**	2	Some concerns	No concerns	Some concerns	No concerns	No concerns	Moderate
**2 vs 10**	1	No concerns	Some concerns	No concerns	No concerns	No concerns	Moderate
**4 vs 10**	3	No concerns	Some concerns	No concerns	No concerns	No concerns	Moderate
**7 vs 10**	2	No concerns	Some concerns	No concerns	No concerns	No concerns	Moderate
**9 vs 10**	1	Some concerns	Some concerns	No concerns	No concerns	No concerns	Moderate
**6 vs 11**	1	No concerns	Major concerns	No concerns	No concerns	No concerns	Low

**3 vs 12**	1	No concerns	Some concerns	No concerns	No concerns	No concerns	Moderate
**5 vs 12**	1	Some concerns	Some concerns	No concerns	No concerns	No concerns	Moderate
**7 vs 12**	1	No concerns	Some concerns	No concerns	Some concerns	No concerns	Moderate
**9 vs 12**	1	Some concerns	No concerns	Some concerns	No concerns	No concerns	Moderate
**4 vs 13**	1	Some concerns	Some concerns	No concerns	No concerns	No concerns	Moderate
**8 vs 13**	2	Some concerns	Some concerns	No concerns	No concerns	No concerns	Moderate
Indirect evidence							
**1 vs 5**	--	Some concerns	Some concerns	No concerns	No concerns	Some concerns	Low
**1 vs 11**	--	No concerns	Some concerns	Some concerns	No concerns	Some concerns	Low
**2 vs 5**	--	Some concerns	Some concerns	No concerns	No concerns	Some concerns	Low
**2 vs 9**	--	Some concerns	Some concerns	No concerns	No concerns	Some concerns	Low
**2 vs 11**	--	No concerns	Major concerns	No concerns	No concerns	Some concerns	Very low
**2 vs 12**	--	No concerns	Some concerns	No concerns	No concerns	Some concerns	Moderate
**2 vs 13**	--	No concerns	Some concerns	No concerns	No concerns	Some concerns	Moderate
**3 vs 4**	--	No concerns	No concerns	Some concerns	No concerns	Some concerns	Moderate
**3 vs 5**	--	Some concerns	Some concerns	No concerns	No concerns	Some concerns	Low
**3 vs 6**	--	No concerns	No concerns	Some concerns	No concerns	Some concerns	Moderate
**3 vs 9**	--	Some concerns	Some concerns	No concerns	No concerns	Some concerns	Low
**3 vs 10**	--	No concerns	No concerns	Some concerns	No concerns	Some concerns	Moderate
**3 vs 11**	--	No concerns	Major concerns	No concerns	No concerns	Some concerns	Very low
**3 vs 13**	--	No concerns	Some concerns	No concerns	No concerns	Some concerns	Moderate
**4 vs 5**	--	Some concerns	Some concerns	No concerns	No concerns	Some concerns	Low
**4 vs 6**	--	Some concerns	No concerns	Major concerns	No concerns	Some concerns	Very low
**4 vs 11**	--	No concerns	Major concerns	No concerns	No concerns	Some concerns	Very low
**4 vs 12**	--	Some concerns	Some concerns	No concerns	No concerns	Some concerns	Low
**5 vs 7**	--	Some concerns	Some concerns	No concerns	No concerns	Some concerns	Low
**5 vs 8**	--	Some concerns	Some concerns	No concerns	No concerns	Some concerns	Low
**5 vs 10**	--	Some concerns	No concerns	Some concerns	No concerns	Some concerns	Low
**5 vs 11**	--	Some concerns	Some concerns	Some concerns	No concerns	Some concerns	Very low
**5 vs 13**	--	Some concerns	Some concerns	No concerns	No concerns	Some concerns	Low
**6 vs 8**	--	Some concerns	Some concerns	No concerns	No concerns	Some concerns	Low

**6 vs 9**	--	Some concerns	Some concerns	No concerns	No concerns	Some concerns	Low
**6 vs 10**	--	Some concerns	Some concerns	No concerns	No concerns	Some concerns	Low
**6 vs 12**	--	Some concerns	Some concerns	No concerns	No concerns	Some concerns	Low
**6 vs 13**	--	Some concerns	Some concerns	No concerns	No concerns	Some concerns	Low
**7 vs 8**	--	No concerns	No concerns	Some concerns	No concerns	Some concerns	Moderate
**7 vs 9**	--	Some concerns	Some concerns	No concerns	No concerns	Some concerns	Low
**7 vs 11**	--	No concerns	Major concerns	No concerns	No concerns	Some concerns	Very low
**7 vs 13**	--	No concerns	Some concerns	No concerns	No concerns	Some concerns	Moderate
**8 vs 10**	--	Some concerns	Some concerns	No concerns	No concerns	Some concerns	Low
**8 vs 11**	--	No concerns	Some concerns	Some concerns	No concerns	Some concerns	Low
**8 vs 12**	--	Some concerns	No concerns	Major concerns	No concerns	Some concerns	Very low
**9 vs 11**	--	Some concerns	Some concerns	No concerns	No concerns	Some concerns	Low
**9 vs 13**	--	Some concerns	Some concerns	No concerns	No concerns	Some concerns	Low
**10 vs 11**	--	No concerns	Some concerns	No concerns	No concerns	Some concerns	Moderate
**10 vs 12**	--	Some concerns	Some concerns	No concerns	No concerns	Some concerns	Low
**10 vs 13**	--	Some concerns	Some concerns	No concerns	No concerns	Some concerns	Low
**11 vs 12**	--	No concerns	Some concerns	Some concerns	No concerns	Some concerns	Very low
**11 vs 13**	--	No concerns	Some concerns	No concerns	No concerns	Some concerns	Moderate
**12 vs 13**	--	Some concerns	Some concerns	No concerns	No concerns	Some concerns	Low

Supplemental Table S28: GRADE evaluation for total-cholesterol and all comparisons between the different oils/solid fats.

[1=Olive oil, 2=Sunflower oil, 3=Rapeseed oil, 4=Soybean oil, 5=Safflower oil, 6=Flaxseed oil, 7=Corn oil, 8=Palm oil, 9=Coconut oil, 10=Butter, 11=Hempseed oil, 12=Beef fat, 13=Lard].

Comparison	Number of studies	Study Limitations	Imprecision	Heterogeneity	Incoherence	Indirectness	Rating
**Mixed Evidence**							
**1 vs 2**	5	No concerns	Some concerns	Some concerns	No concerns	No concerns	Moderate
**1 vs 3**	3	No concerns	Some concerns	Some concerns	No concerns	No concerns	Moderate
**1 vs 4**	2	Some concerns	Some concerns	Some concerns	No concerns	No concerns	Low
**1 vs 6**	1	Some concerns	Some concerns	Some concerns	No concerns	No concerns	Low
**1 vs 7**	5	No concerns	Some concerns	Some concerns	No concerns	No concerns	Moderate
**1 vs 8**	5	Some concerns	No concerns	Major concerns	No concerns	No concerns	Very low
**1 vs 9**	2	Some concerns	Major concerns	No concerns	No concerns	No concerns	Very low
**1 vs 10**	5	Some concerns	No concerns	Some concerns	Some concerns	No concerns	Low
**1 vs 12**	1	No concerns	Major concerns	No concerns	No concerns	No concerns	Low
**1 vs 13**	1	Some concerns	Some concerns	Some concerns	No concerns	No concerns	Low
**2 vs 3**	3	No concerns	Major concerns	No concerns	No concerns	No concerns	Low
**2 vs 4**	1	No concerns	Some concerns	Some concerns	No concerns	No concerns	Moderate
**2 vs 6**	2	No concerns	Major concerns	No concerns	No concerns	No concerns	Low
**2 vs 7**	2	No concerns	Some concerns	Some concerns	No concerns	No concerns	Moderate
**2 vs 8**	6	No concerns	Some concerns	Some concerns	No concerns	No concerns	Moderate
**3 vs 7**	1	No concerns	Some concerns	Some concerns	No concerns	No concerns	Moderate
**3 vs 8**	1	No concerns	Some concerns	Some concerns	No concerns	No concerns	Moderate
**4 vs 7**	2	No concerns	Some concerns	Some concerns	Some concerns	No concerns	Low
**4 vs 8**	4	Some concerns	Some concerns	Some concerns	Some concerns	No concerns	Low
**4 vs 9**	1	Some concerns	Some concerns	Some concerns	Some concerns	No concerns	Low
**5 vs 6**	1	No concerns	Major concerns	No concerns	No concerns	No concerns	Low
**5 vs 9**	2	Some concerns	Some concerns	Some concerns	No concerns	No concerns	Low
**6 vs 7**	1	No concerns	Major concerns	No concerns	No concerns	No concerns	Low
**8 vs 9**	2	Some concerns	Major concerns	No concerns	No concerns	No concerns	Very low
**2 vs 10**	1	No concerns	No concerns	Some concerns	No concerns	No concerns	Moderate
**4 vs 10**	3	No concerns	No concerns	Some concerns	No concerns	No concerns	Moderate
**7 vs 10**	2	No concerns	No concerns	Some concerns	No concerns	No concerns	Moderate
**9 vs 10**	1	Some concerns	Some concerns	No concerns	No concerns	No concerns	Moderate
**6 vs 11**	1	No concerns	Major concerns	No concerns	No concerns	No concerns	Low

**3 vs 12**	1	No concerns	Major concerns	No concerns	No concerns	No concerns	Low
**5 vs 12**	1	Some concerns	Some concerns	Some concerns	No concerns	No concerns	Low
**7 vs 12**	1	No concerns	Major concerns	No concerns	No concerns	No concerns	Low
**9 vs 12**	1	Some concerns	Major concerns	No concerns	No concerns	No concerns	Very low
**4 vs 13**	1	Some concerns	Some concerns	No concerns	No concerns	No concerns	Moderate
**8 vs 13**	2	Some concerns	Some concerns	Some concerns	No concerns	No concerns	Low
**Indirect Evidence**							
**1 vs 5**	--	Some concerns	Some concerns	Some concerns	No concerns	Some concerns	Very low
**1 vs 11**	--	No concerns	Major concerns	No concerns	No concerns	Some concerns	Very low
**2 vs 5**	--	Some concerns	Major concerns	No concerns	No concerns	Some concerns	Very low
**2 vs 9**	--	Some concerns	Some concerns	Some concerns	No concerns	Some concerns	Very low
**2 vs 11**	--	No concerns	Major concerns	No concerns	No concerns	Some concerns	Very low
**2 vs 12**	--	No concerns	Major concerns	No concerns	No concerns	Some concerns	Very low
**2 vs 13**	--	No concerns	Some concerns	No concerns	No concerns	Some concerns	Moderate
**3 vs 4**	--	No concerns	Some concerns	Some concerns	No concerns	Some concerns	Low
**3 vs 5**	--	Some concerns	Major concerns	No concerns	No concerns	Some concerns	Very low
**3 vs 6**	--	No concerns	Major concerns	No concerns	No concerns	Some concerns	Very low
**3 vs 9**	--	Some concerns	Some concerns	Some concerns	No concerns	Some concerns	Very low
**3 vs 10**	--	No concerns	No concerns	Some concerns	No concerns	Some concerns	Moderate
**3 vs 12**	--	No concerns	Major concerns	No concerns	No concerns	Some concerns	Very low
**3 vs 13**	--	No concerns	Some concerns	No concerns	No concerns	Some concerns	Moderate
**4 vs 5**	--	Some concerns	Some concerns	Some concerns	No concerns	Some concerns	Very low
**4 vs 6**	--	Some concerns	Some concerns	Some concerns	No concerns	Some concerns	Very low
**4 vs 11**	--	No concerns	Major concerns	No concerns	No concerns	Some concerns	Very low
**4 vs 12**	--	No concerns	Major concerns	No concerns	No concerns	Some concerns	Very low
**5 vs 7**	--	Some concerns	Major concerns	No concerns	No concerns	Some concerns	Very low
**5 vs 8**	--	Some concerns	Some concerns	Some concerns	No concerns	Some concerns	Very low
**5 vs 10**	--	Some concerns	No concerns	No concerns	No concerns	Some concerns	Moderate
**5 vs 11**	--	No concerns	Major concerns	No concerns	No concerns	Some concerns	Very low
**5 vs 13**	--	Some concerns	Some concerns	No concerns	No concerns	Some concerns	Low
**6 vs 8**	--	Some concerns	Some concerns	Some concerns	No concerns	Some concerns	Very low
**6 vs 9**	--	Some concerns	Some concerns	Some concerns	No concerns	Some concerns	Very low

**6 vs 10**	--	Some concerns	No concerns	Some concerns	No concerns	Some concerns	Low
**6 vs 12**	--	No concerns	Major concerns	No concerns	No concerns	Some concerns	Very low
**6 vs 13**	--	Some concerns	Some concerns	No concerns	No concerns	Some concerns	Low
**7 vs 8**	--	No concerns	Some concerns	Some concerns	No concerns	Some concerns	Low
**7 vs 9**	--	Some concerns	Some concerns	Some concerns	No concerns	Some concerns	Very low
**7 vs 11**	--	No concerns	Major concerns	No concerns	No concerns	Some concerns	Very low
**7 vs 13**	--	No concerns	Some concerns	No concerns	No concerns	Some concerns	Moderate
**8 vs 10**	--	Some concerns	No concerns	Some concerns	No concerns	Some concerns	Low
**8 vs 11**	--	No concerns	Major concerns	No concerns	No concerns	Some concerns	Very low
**8 vs 12**	--	Some concerns	Major concerns	No concerns	No concerns	Some concerns	Very low
**9 vs 11**	--	Some concerns	Major concerns	No concerns	No concerns	Some concerns	Very low
**9 vs 13**	--	Some concerns	Some concerns	Some concerns	No concerns	Some concerns	Very low
**10 vs 11**	--	No concerns	Some concerns	No concerns	No concerns	Some concerns	Moderate
**10 vs 12**	--	Some concerns	Some concerns	No concerns	No concerns	Some concerns	Low
**10 vs 13**	--	Some concerns	Major concerns	No concerns	No concerns	Some concerns	Very low
**11 vs 12**	--	No concerns	Major concerns	No concerns	No concerns	Some concerns	Very low
**11 vs 13**	--	No concerns	Some concerns	Some concerns	No concerns	Some concerns	Low
**12 vs 13**	--	Some concerns	Some concerns	Some concerns	No concerns	Some concerns	Very low

Supplemental Table S29: GRADE evaluation for LDL-cholesterol and all comparisons between the different oils/solid fats.

[1=Olive oil, 2=Sunflower oil, 3=Rapeseed oil, 4=Soybean oil, 5=Safflower oil, 6=Flaxseed oil, 7=Corn oil, 8=Palm oil, 9=Coconut oil, 10=Butter, 11=Hempseed oil, 12=Beef fat, 13=Lard].

Comparison	Number of studies	Study Limitations	Imprecision	Heterogeneity	Incoherence	Indirectness	Rating
**Mixed Evidence**							
**1 vs 2**	5	No concerns	No concerns	No concerns	No concerns	No concerns	High
**1 vs 3**	4	No concerns	No concerns	Some concerns	No concerns	No concerns	Moderate
**1 vs 4**	2	Some concerns	Some concerns	No concerns	No concerns	No concerns	Moderate
**1 vs 6**	1	Some concerns	Some concerns	No concerns	No concerns	No concerns	Moderate
**1 vs 7**	5	No concerns	Some concerns	No concerns	No concerns	No concerns	Moderate
**1 vs 8**	5	Some concerns	Some concerns	No concerns	No concerns	No concerns	Moderate
**1 vs 9**	2	Some concerns	Some concerns	No concerns	Some concerns	No concerns	Low
**1 vs 10**	5	Some concerns	No concerns	Some concerns	No concerns	No concerns	Moderate
**1 vs 12**	1	Some concerns	Some concerns	No concerns	No concerns	No concerns	Moderate
**1 vs 13**	1	Some concerns	Major concerns	No concerns	No concerns	No concerns	Very low
**2 vs 3**	3	No concerns	Major concerns	No concerns	No concerns	No concerns	Low
**2 vs 4**	1	No concerns	Some concerns	No concerns	Some concerns	No concerns	Moderate
**2 vs 6**	2	No concerns	Some concerns	No concerns	No concerns	No concerns	Moderate
**2 vs 7**	2	No concerns	Some concerns	No concerns	No concerns	No concerns	Moderate
**2 vs 8**	6	No concerns	Some concerns	No concerns	No concerns	No concerns	Moderate
**2 vs 9**	1	No concerns	Some concerns	No concerns	No concerns	No concerns	Moderate
**3 vs 7**	1	No concerns	Some concerns	No concerns	No concerns	No concerns	Moderate
**3 vs 8**	1	No concerns	Some concerns	No concerns	No concerns	No concerns	Moderate
**4 vs 7**	2	No concerns	Some concerns	No concerns	Some concerns	No concerns	Moderate
**4 vs 8**	4	Some concerns	No concerns	No concerns	Some concerns	No concerns	Moderate
**4 vs 9**	1	No concerns	No concerns	No concerns	No concerns	No concerns	High
**5 vs 6**	2	Some concerns	Some concerns	No concerns	No concerns	No concerns	Moderate
**5 vs 9**	2	Some concerns	No concerns	No concerns	No concerns	No concerns	Moderate
**6 vs 7**	1	No concerns	Major concerns	No concerns	No concerns	No concerns	Low
**7 vs 9**	1	No concerns	Some concerns	No concerns	No concerns	No concerns	Moderate
**8 vs 9**	2	Some concerns	Major concerns	No concerns	No concerns	No concerns	Very low
**4 vs 10**	3	No concerns	Some concerns	No concerns	No concerns	No concerns	Moderate
**7 vs 10**	1	No concerns	Some concerns	No concerns	No concerns	No concerns	Moderate
**9 vs 10**	1	Some concerns	Some concerns	No concerns	Some concerns	No concerns	Low

**6 vs 11**	1	No concerns	Major concerns	No concerns	No concerns	No concerns	Low
**3 vs 12**	1	No concerns	Major concerns	No concerns	No concerns	No concerns	Low
**5 vs 12**	1	Some concerns	No concerns	No concerns	No concerns	No concerns	Moderate
**7 vs 12**	1	No concerns	Some concerns	No concerns	No concerns	No concerns	Moderate
**9 vs 12**	1	Some concerns	Major concerns	No concerns	No concerns	No concerns	Very low
**4 vs 13**	1	Some concerns	Some concerns	No concerns	No concerns	No concerns	Moderate
**8 vs 13**	2	Some concerns	Some concerns	No concerns	No concerns	No concerns	Moderate
**Indirect Evidence**							
**1 vs 5**	--	Some concerns	Some concerns	No concerns	No concerns	Some concerns	Low
**1 vs 11**	--	No concerns	Major concerns	No concerns	No concerns	Some concerns	Very low
**2 vs 5**	--	Some concerns	Some concerns	No concerns	No concerns	Some concerns	Low
**2 vs 10**	--	No concerns	Some concerns	No concerns	No concerns	Some concerns	Moderate
**2 vs 11**	--	No concerns	Major concerns	No concerns	No concerns	Some concerns	Very low
**2 vs 12**	--	No concerns	Some concerns	Some concerns	No concerns	Some concerns	Low
**2 vs 13**	--	No concerns	Major concerns	No concerns	No concerns	Some concerns	Very low
**3 vs 4**	--	No concerns	Some concerns	No concerns	No concerns	Some concerns	Moderate
**3 vs 5**	--	Some concerns	Some concerns	No concerns	No concerns	Some concerns	Low
**3 vs 6**	--	No concerns	Some concerns	No concerns	No concerns	Some concerns	Moderate
**3 vs 9**	--	No concerns	Some concerns	No concerns	No concerns	Some concerns	Moderate
**3 vs 10**	--	No concerns	Major concerns	No concerns	No concerns	Some concerns	Very low
**3 vs 11**	--	No concerns	Major concerns	No concerns	No concerns	Some concerns	Very low
**3 vs 12**	--	No concerns	Major concerns	No concerns	No concerns	Some concerns	Very low
**4 vs 5**	--	Some concerns	Some concerns	No concerns	No concerns	Some concerns	Low
**4 vs 6**	--	Some concerns	Major concerns	No concerns	No concerns	Some concerns	Very low
**4 vs 11**	--	No concerns	Major concerns	No concerns	No concerns	Some concerns	Very low
**4 vs 12**	--	Some concerns	Some concerns	No concerns	No concerns	Some concerns	Low
**5 vs 7**	--	Some concerns	Some concerns	No concerns	No concerns	Some concerns	Low
**5 vs 8**	--	Some concerns	No concerns	No concerns	No concerns	Some concerns	Moderate
**5 vs 10**	--	Some concerns	Some concerns	No concerns	No concerns	Some concerns	Low
**5 vs 11**	--	Some concerns	Major concerns	No concerns	No concerns	Some concerns	Very low
**5 vs 12**	--	Some concerns	Some concerns	No concerns	No concerns	Some concerns	Low

**6 vs 8**	--	Some concerns	Some concerns	No concerns	No concerns	Some concerns	Low
**6 vs 9**	--	Some concerns	Some concerns	No concerns	No concerns	Some concerns	Low
**6 vs 11**	--	Some concerns	Some concerns	Some concerns	No concerns	Some concerns	Very low
**6 vs 12**	--	Some concerns	Some concerns	No concerns	No concerns	Some concerns	Low
**6 vs 13**	--	Some concerns	Some concerns	No concerns	No concerns	Some concerns	Low
**7 vs 8**	--	No concerns	Some concerns	No concerns	No concerns	Some concerns	Low
**7 vs 11**	--	No concerns	Major concerns	No concerns	No concerns	Some concerns	Very low
**7 vs 13**	--	No concerns	Some concerns	No concerns	No concerns	Some concerns	Moderate
**8 vs 10**	--	Some concerns	Some concerns	No concerns	No concerns	Some concerns	Low
**8 vs 11**	--	No concerns	Major concerns	No concerns	No concerns	Some concerns	Very low
**8 vs 12**	--	Some concerns	Major concerns	No concerns	No concerns	Some concerns	Very low
**9 vs 11**	--	Some concerns	Major concerns	No concerns	No concerns	Some concerns	Very low
**9 vs 13**	--	Some concerns	Some concerns	No concerns	No concerns	Some concerns	Low
**10 vs 11**	--	No concerns	Major concerns	No concerns	No concerns	Some concerns	Very low
**10 vs 12**	--	Some concerns	Some concerns	No concerns	No concerns	Some concerns	Low
**10 vs 13**	--	Some concerns	Major concerns	No concerns	No concerns	Some concerns	Very low
**11 vs 12**	--	Some concerns	Major concerns	No concerns	No concerns	Some concerns	Very low
**11 vs 13**	--	No concerns	Major concerns	No concerns	No concerns	Some concerns	Very low
**12 vs 13**	--	Some concerns	Major concerns	No concerns	No concerns	Some concerns	Very low

Supplemental Table S30: GRADE evaluation for HDL-cholesterol and all comparisons between the different oils/solid fats.

[1=Olive oil, 2=Sunflower oil, 3=Rapeseed oil, 4=Soybean oil, 5=Safflower oil, 6=Flaxseed oil, 7=Corn oil, 8=Palm oil, 9=Coconut oil, 10=Butter, 11=Hempseed oil, 12=Beef fat, 13=Lard].

Comparison	Number of studies	Study Limitations	Imprecision	Heterogeneity	Incoherence	Indirectness	Rating
**Mixed Evidence**							
**1 vs 2**	5	No concerns	No concerns	No concerns	No concerns	No concerns	High
**1 vs 3**	4	No concerns	No concerns	No concerns	No concerns	No concerns	Moderate
**1 vs 4**	2	Some concerns	No concerns	No concerns	No concerns	No concerns	Moderate
**1 vs 6**	1	Some concerns	No concerns	No concerns	No concerns	No concerns	High
**1 vs 7**	5	No concerns	No concerns	No concerns	No concerns	No concerns	Moderate
**1 vs 8**	5	Some concerns	No concerns	No concerns	No concerns	No concerns	Moderate
**1 vs 9**	2	Some concerns	No concerns	No concerns	No concerns	No concerns	Moderate
**1 vs 10**	5	Some concerns	No concerns	No concerns	No concerns	No concerns	Moderate
**1 vs 12**	1	Some concerns	No concerns	No concerns	No concerns	No concerns	Moderate
**1 vs 13**	1	Some concerns	No concerns	No concerns	No concerns	No concerns	Moderate
**2 vs 3**	3	No concerns	No concerns	No concerns	No concerns	No concerns	High
**2 vs 6**	2	No concerns	No concerns	No concerns	No concerns	No concerns	High
**2 vs 7**	1	No concerns	No concerns	No concerns	No concerns	No concerns	High
**2 vs 8**	7	No concerns	No concerns	No concerns	No concerns	No concerns	High
**3 vs 7**	1	No concerns	No concerns	No concerns	No concerns	No concerns	High
**3 vs 8**	1	No concerns	No concerns	No concerns	No concerns	No concerns	High
**4 vs 7**	1	No concerns	No concerns	No concerns	No concerns	No concerns	High
**4 vs 8**	4	Some concerns	No concerns	No concerns	No concerns	No concerns	Moderate
**4 vs 9**	1	Some concerns	No concerns	No concerns	No concerns	No concerns	Moderate
**5 vs 6**	2	Some concerns	No concerns	No concerns	No concerns	No concerns	Moderate
**5 vs 9**	2	Some concerns	No concerns	No concerns	No concerns	No concerns	Moderate
**6 vs 7**	1	Some concerns	No concerns	No concerns	No concerns	No concerns	Moderate
**8 vs 9**	2	Some concerns	No concerns	No concerns	No concerns	No concerns	Moderate
**2 vs 10**	1	No concerns	No concerns	No concerns	No concerns	No concerns	High
**4 vs 10**	3	No concerns	No concerns	No concerns	No concerns	No concerns	High
**7 vs 10**	2	No concerns	No concerns	No concerns	No concerns	No concerns	High
**9 vs 10**	1	Some concerns	No concerns	No concerns	No concerns	No concerns	Moderate
**6 vs 11**	1	No concerns	No concerns	No concerns	No concerns	No concerns	High
**3 vs 12**	1	No concerns	No concerns	No concerns	No concerns	No concerns	High

**5 vs 12**	1	Some concerns	No concerns	No concerns	No concerns	No concerns	Moderate
**7 vs 12**	1	No concerns	No concerns	No concerns	No concerns	No concerns	High
**9 vs 12**	1	Some concerns	No concerns	No concerns	No concerns	No concerns	Moderate
**4 vs 13**	1	Some concerns	No concerns	No concerns	No concerns	No concerns	Moderate
**8 vs 13**	2	Some concerns	No concerns	No concerns	No concerns	No concerns	Moderate
**Indirect Evidence**							
**1 vs 5**	--	Some concerns	No concerns	No concerns	No concerns	Some concerns	Moderate
**1 vs 11**	--	No concerns	No concerns	No concerns	No concerns	Some concerns	Moderate
**2 vs 4**	--	No concerns	No concerns	No concerns	No concerns	Some concerns	Moderate
**2 vs 5**	--	Some concerns	No concerns	No concerns	No concerns	Some concerns	Moderate
**2 vs 9**	--	Some concerns	No concerns	No concerns	No concerns	Some concerns	Moderate
**2 vs 11**	--	No concerns	No concerns	No concerns	No concerns	Some concerns	Moderate
**2 vs 12**	--	Some concerns	No concerns	No concerns	No concerns	Some concerns	Moderate
**2 vs 13**	--	No concerns	No concerns	No concerns	No concerns	Some concerns	Moderate
**3 vs 4**	--	No concerns	No concerns	No concerns	No concerns	Some concerns	Moderate
**3 vs 5**	--	Some concerns	No concerns	No concerns	No concerns	Some concerns	Moderate
**3 vs 6**	--	No concerns	No concerns	No concerns	No concerns	Some concerns	Moderate
**3 vs 9**	--	Some concerns	No concerns	No concerns	No concerns	Some concerns	Moderate
**3 vs 10**	--	No concerns	No concerns	No concerns	No concerns	Some concerns	Moderate
**3 vs 11**	--	No concerns	No concerns	No concerns	No concerns	Some concerns	Moderate
**3 vs 13**	--	No concerns	No concerns	No concerns	No concerns	Some concerns	Moderate
**4 vs 5**	--	Some concerns	No concerns	No concerns	No concerns	Some concerns	Moderate
**4 vs 6**	--	Some concerns	No concerns	No concerns	No concerns	Some concerns	Moderate
**4 vs 11**	--	Some concerns	No concerns	No concerns	No concerns	Some concerns	Moderate
**4 vs 12**	--	Some concerns	No concerns	No concerns	No concerns	Some concerns	Moderate
**5 vs 7**	--	Some concerns	No concerns	No concerns	No concerns	Some concerns	Moderate
**5 vs 8**	--	Some concerns	No concerns	No concerns	No concerns	Some concerns	Moderate
**5 vs 10**	--	Some concerns	No concerns	No concerns	No concerns	Some concerns	Moderate
**5 vs 11**	--	Some concerns	No concerns	No concerns	No concerns	Some concerns	Moderate
**5 vs 13**	--	Some concerns	No concerns	No concerns	No concerns	Some concerns	Moderate
**6 vs 8**	--	Some concerns	No concerns	No concerns	No concerns	Some concerns	Moderate

**6 vs 9**	--	Some concerns	No concerns	No concerns	No concerns	Some concerns	Moderate
**6 vs 10**	--	Some concerns	No concerns	No concerns	No concerns	Some concerns	Moderate
**6 vs 12**	--	Some concerns	No concerns	No concerns	No concerns	Some concerns	Moderate
**6 vs 13**	--	Some concerns	No concerns	No concerns	No concerns	Some concerns	Moderate
**7 vs 8**	--	No concerns	No concerns	No concerns	No concerns	Some concerns	Moderate
**7 vs 9**	--	Some concerns	No concerns	No concerns	No concerns	Some concerns	Moderate
**7 vs 11**	--	No concerns	No concerns	No concerns	No concerns	Some concerns	Moderate
**7 vs 13**	--	No concerns	No concerns	No concerns	No concerns	Some concerns	Moderate
**8 vs 10**	--	Some concerns	No concerns	No concerns	No concerns	Some concerns	Moderate
**8 vs 11**	--	Some concerns	No concerns	No concerns	No concerns	Some concerns	Moderate
**8 vs 12**	--	Some concerns	No concerns	No concerns	No concerns	Some concerns	Moderate
**9 vs 11**	--	Some concerns	No concerns	No concerns	No concerns	Some concerns	Moderate
**9 vs 13**	--	Some concerns	No concerns	No concerns	No concerns	Some concerns	Moderate
**10 vs 11**	--	Some concerns	No concerns	No concerns	No concerns	Some concerns	Moderate
**10 vs 12**	--	Some concerns	No concerns	No concerns	No concerns	Some concerns	Moderate
**10 vs 13**	--	Some concerns	No concerns	No concerns	No concerns	Some concerns	Moderate
**11 vs 12**	--	Some concerns	Some concerns	No concerns	No concerns	Some concerns	Low
**11 vs 13**	--	Some concerns	No concerns	No concerns	No concerns	Some concerns	Moderate
**12 vs 13**	--	Some concerns	No concerns	No concerns	No concerns	Some concerns	Moderate

Supplemental Table S31: GRADE evaluation for triacyglycerols and all comparisons between the different oils/solid fats.

[1=Olive oil, 2=Sunflower oil, 3=Rapeseed oil, 4=Soybean oil, 5=Safflower oil, 6=Flaxseed oil, 7=Corn oil, 8=Palm oil, 9=Coconut oil, 10=Butter, 11=Hempseed oil, 12=Beef fat, 13=Lard].
